# A citizen science approach estimating titanium dioxide released from personal care products

**DOI:** 10.1371/journal.pone.0235988

**Published:** 2020-07-29

**Authors:** Fan Wu, Matt Seib, Samantha Mauel, Sydney Klinzing, Andrea L. Hicks

**Affiliations:** 1 School of Environment, Guangdong Key Laboratory of Environmental Pollution and Health, Jinan University, Guangzhou, China; 2 Department of Civil and Environmental Engineering, University of Wisconsin-Madison, Madison, WI, United States of America; 3 Madison Metropolitan Sewerage District, Madison, WI, United States of America; VIT University, INDIA

## Abstract

Titanium dioxide (TiO_2_) is a common component in personal care products (PCP), which through use enters the wastewater treatment plant (WWTP) and ultimately the environment. A citizen science approach is utilized here to inform the prevalence and usage of TiO_2_ containing PCP on a household scale, which generates information as to the quantity of TiO_2_ entering the WWTP, and the portion ultimately discharged to the environment. Meanwhile, citizen science sourced inventories were generated to estimate the quantity of TiO_2_, and potentially nanoscale TiO_2_ entering the WWTP from consumer products and to determine which products had the greatest contribution. The estimated values were compared with water samples from the WWTP which quantified the amount of total titanium present using ICP-AES. These values were at a similar level with other top-down estimation approaches and suggest that a citizen science approach is valid to estimate the loading of TiO_2_, and potentially other emerging contaminants, while at the same time engaging with community stakeholders.

## 1. Introduction

The triple bottom line of sustainability is comprised of three aspects: environment, economy, and society. The social aspect is often considered the most challenging to quantify, but at the same time provides a great deal of opportunity to understand the role of human behavior and its relation to sustainability [1]. With more public participation in scientific projects, it is flourishing globally as part of projects labeled “citizen science” [2]. This work seeks to utilize the social aspect of sustainability through a citizen science-based approach to quantify a potential contaminant entering the environment through human use of personal care products (PCP).

Titanium dioxide (TiO_2_) is a naturally occurring metal oxide. The global production of TiO_2_ was approximately 6.1 million metric tons in 2016 and is projected to reach 7.8 million tons by 2022; furthermore, the global TiO_2_ market currently valued at $13.3 billion is expected to grow at 8.9% annually through 2025 [3]. TiO_2_ found in PCPs is largely formulated at the nanoscale (one or more dimensions equals or less than 100 nm) to achieve specific applications such as blocking ultraviolet radiation (UV)” and adding texture and brightness to the products [4, 5]. Previous research not only indicates a potential for TiO_2_ nanoparticles (NPs) to cause human health risks via inhalation, they also suggest engineered TiO_2_ NP could pose potential environmental health concerns due to characteristics such as reactive oxygen species generation, cell membrane disruption, and easy attachment to intracellular organelles and biological macromolecules [6, 7]. Specific usage of TiO_2_ NPs is not clearly regulated, especially in PCPs; additionally, knowledge on the potential human releases of TiO_2_ NPs to the environment is limited. Although a European Commission report has recently reduced the upper allowable limit from 25% to 5.5% for nano-form TiO_2_ as UV-filters in sunscreens and personal care spray products in Europe [8], it remains as one of the top five engineered nanomaterials commonly used in consumer products, paints, and pharmaceutical preparations worldwide [6, 9].

Studies found that up to 36% of TiO_2_ used in certain PCPs can be present at the nanoscale [10–14]. Due to their wide applications in food and PCPs, the majority of TiO_2_ NPs disposed from households pass through the local sewage systems [14–17].Wastewater treatment plants (WWTPs) are often considered as an important point source for the discharge of engineered nanomaterials to natural surface water [18]. Meanwhile, nanoparticles can eventually enter other environmental compartments (surface water, soil, etc.) through various pathways [19, 20]. Although TiO_2_ NPs are relatively stable compared to other metal and metal oxides (such as Ag, ZnO, CuO NPs) due to their low solubility in the aqueous environment, they can aggregate and accumulate in activated sludge [21]. Some studies indicate TiO_2_ NPs have no effect on the wastewater treatment process efficiency, however, their removal efficiency for NPs can vary dramatically depending on the location and technology used in the WWTP, such as whether sewage undergoes secondary or higher treatment, the performance of WWTPs and NP physicochemical characteristics, etc. [22–25]. Taken together, the uncertainty around TiO_2_ NP fate and transport along with perceived ubiquity makes it urgent to understand the quantity released from human daily activities.

The flow of NPs, including TiO_2_, throughout life cycles has been investigated in literature from a top down macroscale perspective [17, 26, 27, 28]. However, a data gap still exists at the individual household and regional levels. Compared to a top-down approach, a bottom-up study gathers detailed information at the household level. This data can be extrapolated to regional and national levels while simultaneously accounting for the heterogeneity of the households and their PCP usage. Previous study has used the same citizen science approach to investigate the human exposure to TiO_2_ from the daily use of PCP [29]. The present work utilizes a bottom-up citizen science approach with data from the sewerage district serving the Madison, Wisconsin metropolitan area in the United States to estimate the quantity of TiO_2_ entering the WWTP from PCP usage. Furthermore, a citizen scientist roundtable was held at the University of Wisconsin-Madison campus to present the findings and gain insights from the citizen participants with respect to their concerns and future research regarding this work. However, due to the limited information on whether the PCP contains nano or non-nano TiO_2_, data were gathered from a series of publications to estimate the upper and lower range of TiO_2_ (potentially nano-TiO_2_) released from different types of household PCPs. This project seeks to collect household level data from citizens in order to quantify and refine the estimates of TiO_2_ (potentially nano-TiO_2_) entering the WWTP as a function of PCPs usage; meanwhile, validating the implementation of the citizen science approach to estimate the inflow of other emerging contaminants. Moreover, the current study engages with the broader community through this “two-way flow” of information to generate and share scientific insights is a unique method to conduct an analysis of TiO_2_ sources at the WWTP.

## 2. Methods

### 2.1. Survey distribution and collection

Summary explanation of the demographical information collected from the survey is presented in the S1 File. Details of the methodology are illustrated in S1 Fig in S1 File. This study employed a social survey to estimate TiO_2_ release to the WWTP from household PCPs usage and disposal. The survey was broken into several sections by PCP category including: toothpaste, shampoo, conditioner, lotion/skin cream, sunblock/sunscreen, deodorant/antiperspirant, shaving cream, other products, and demographic section. The disposal of PCP should theoretically go through the drain and collected in local wastewater input stream. This is supported by other studies, where 96–98% of the PCP material flows pass through the WWTPs [17]. The demographics section asked for the respondent’s gender, age, race, number of residences in the household, and approximate household income. The Madison metropolitan household income data (2016) was used to correct the surveyed populations and predict the overall Ti concentration emitted to the WWTP (S2 Fig in S1 File). The full survey, details on its administration and the validation criteria are provided in the S1 File.

### 2.2 Wastewater sampling and Ti quantification

Weekly flow proportional composite wastewater samples were collected from April 29 to August 26, 2018. Sample digestion was conducted using EPA method 3050b and analyzed using Induced Coupled Plasma-atomic emission spectroscopy (ICP-AES, JY, Ultima 2), where Ti has a recovery rate up to 96% [30].

Multiple studies have surveyed the frequency and quantity consumers apply PCPs in their daily routines [31–36], which are summarized in S1 Table in S1 File. Eqs (1) and (2) are used to calculate the Ti released from individual household.

$$Factor=\frac{\#of\;PCPs\;contain\;TiO_{2}}{\#of\;PCPs\;in\;household}$$ (1)

$$Household\;Ti\;release\;from\;each\;PCP\;(mg)=Factor\times Amount\;PCPs\;used\;(g)\times Residents\;in\;household\times Con.\;of\;TiO_{2}\;in\;PCPs\;\left(\frac{mg\;Ti}{g}\right)$$ (2)

### 2.3 Roundtable meeting and outreach

A roundtable meeting was hosted on University of Wisconsin-Madison campus on Jan. 24^th^, 2019. Eight participants responded and participated the roundtable meeting. The data set is drawn from feedback from eight participants over a two-hour roundtable discussion session and includes 10 pages of researcher notes. All participants were citizen scientists who had taken the social survey and were invested in learning the outcomes of the study and contributing to future research. Participants were given several prompts to respond to during the roundtable discussion session. The common themes of their answers included feelings about TiO_2_, NPs in PCPs, and possible next steps in research.

The roundtable had three main components: a hands-on activity, a presentation of results from the study, and a group facilitated discussion. The hands-on activity compared the properties and characteristics between nano and non-nano formulated PCPs in order to achieve educational purposes. Following the small group activities, the results of the study were presented to the group. Participants were encouraged to ask questions about the findings after the conclusion of the presentation. After informing the participants of the results, the group began a facilitated discussion of the results and possible next steps. The discussion was led by one facilitator, and all participants were encouraged to share their thoughts. Participants were also encouraged to share any questions they had throughout the roundtable discussion. Each of the facilitators recorded the discussion using either handwritten or typed notes.

### 2.4 Ethics statement

Research survey utilized in this study was approved by the Institutional Review Board at University of Wisconsin-Madison (IRB 2017–0883). All individuals have provided consent for the use of their information in each survey.

## 3. Results and discussion

### 3.1 Survey responses

**Fig 1:**
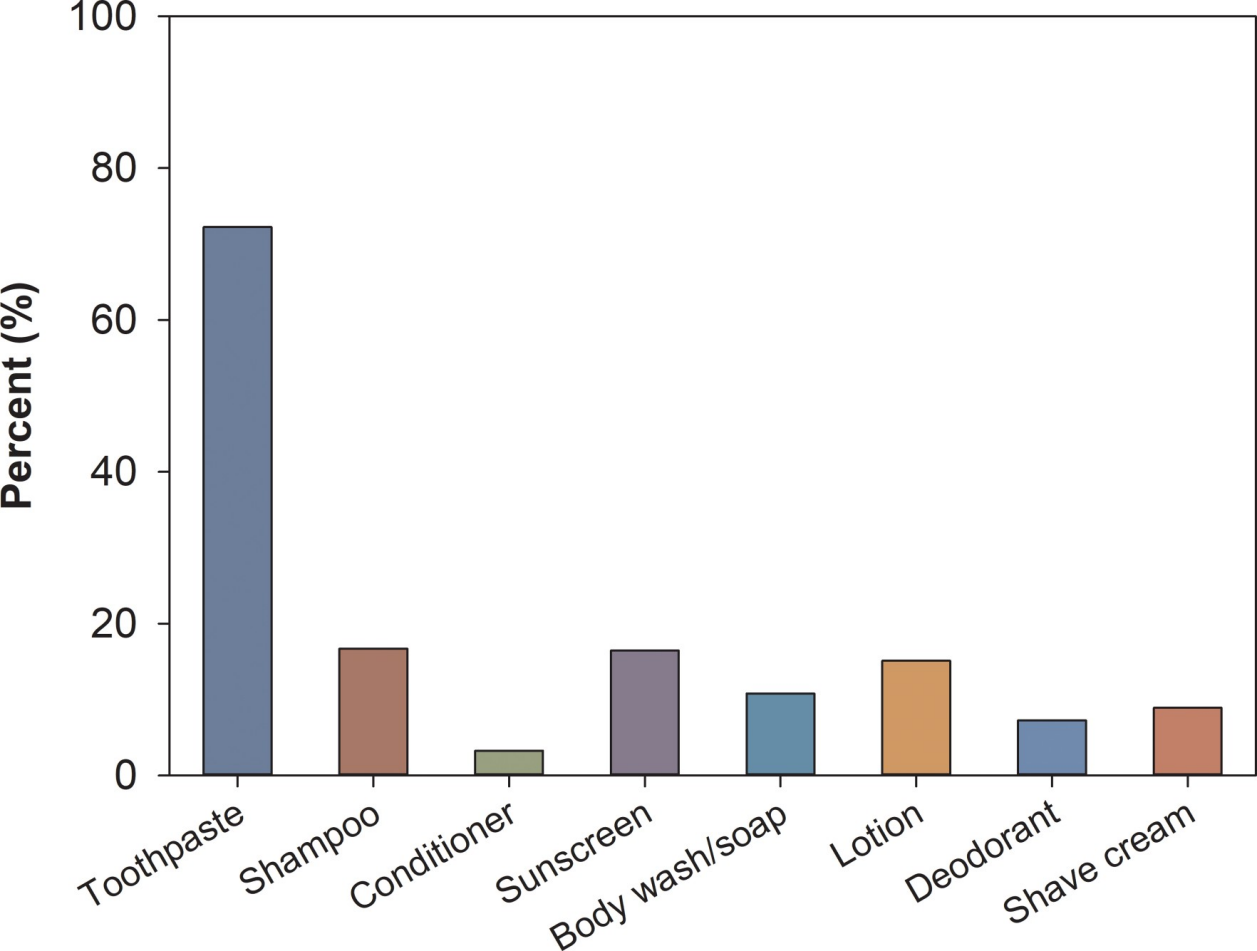
Percent of household products surveyed that listed TiO_2_ as an ingredient.

A survey of citizen scientists living within the WWTP service area was utilized to build an inventory of PCPs (Approved by the Institutional Review Board at UW-Madison, IRB 2017–0883). Demographical information collected from the survey is presented in the SI (Survey results) 1. Based on the identification by consumers, and through evaluating the listed ingredients on the products, the percentage of PCPs in various products that contain TiO_2_ were identified (Fig 1). The majority of the toothpaste was identified to contain TiO_2_ in their ingredients (72.2%), following by shampoo (16.7%), sunscreen (16.4%), lotion (15.1%), and bodywash/soap (10.8%). Products in the remaining categories contained TiO_2_ at a lesser frequency, such as shave cream (8.9%), deodorant (7.3%), and conditioner (3.2%). Using the survey responses, an inventory including the brand name, ingredient list, and corresponding product link was developed (SI 2). This first community sourced TiO_2_ containing PCP inventory includes a total of 213 products (67 toothpaste, 27 shampoo, 5 conditioner, 32 lotion/skin care, 32 sunscreen, 1 deodorant, 9 shaving cream, 18 shower gel/soap, 22 others) that identified TiO_2_ used as an ingredient. The assertion as to whether the product does or does not contain TiO_2_ was confirmed through checking the product ingredient lists.

### 3.2 Household release of TiO_2_

**Fig 2:**
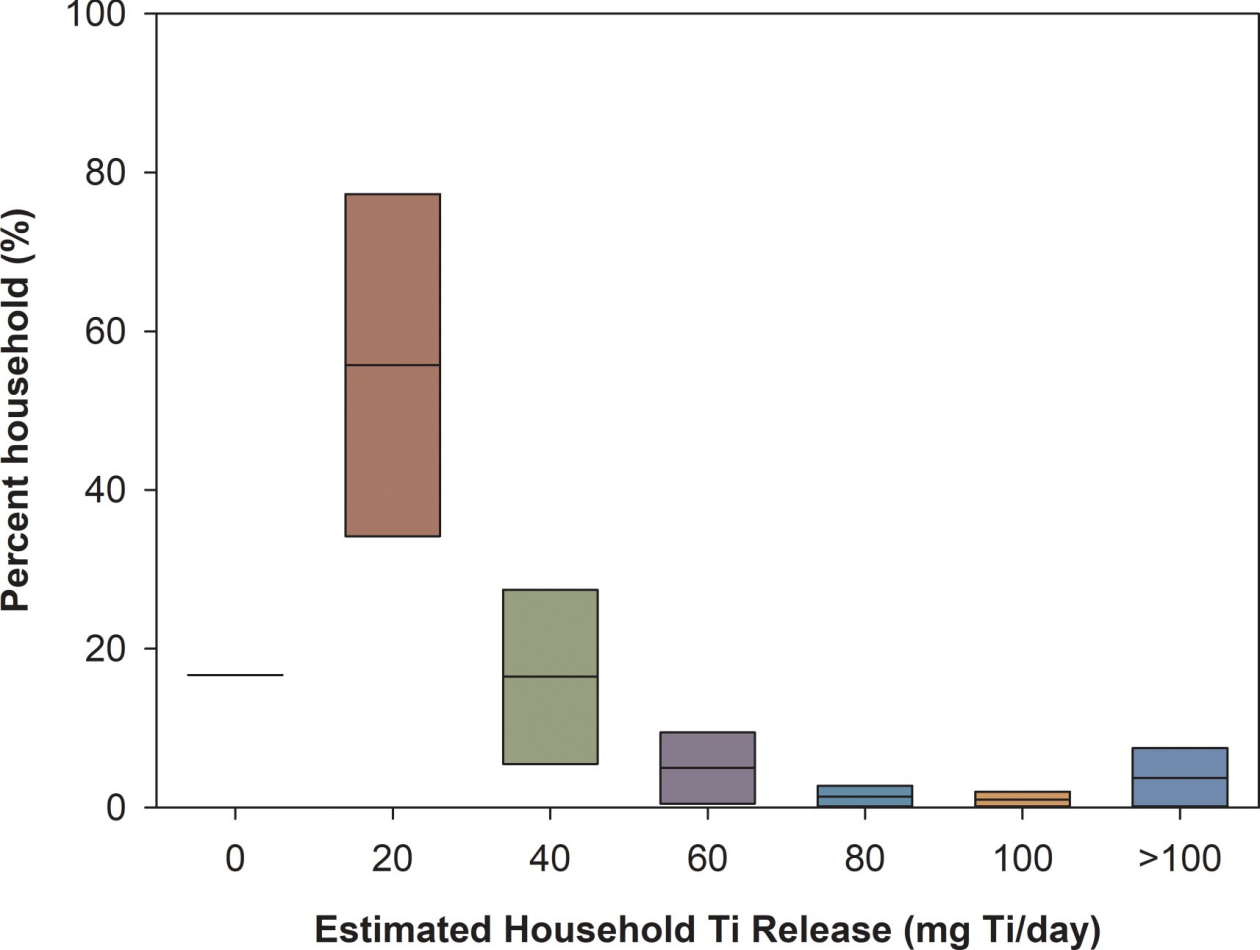
Distribution of daily TiO_2_-Ti release profile from the household PCPs. This is based on the estimated lower and upper Ti concentrations quantified in PCPs. The concentrations in the x-axis represents range values (For instance, 20 mg Ti/day indicates the predicted household release rages within 0–20 mg Ti/day). More detailed figure is presented in S3 Fig in S1 File.

By incorporating the range of TiO_2_ concentrations in PCPs and the usage pattern from literature (summarized in S1 Table in S1 File), the overall household daily TiO_2_-Ti (TiO_2_ release quantified as Ti) release distributions under lower and upper bound conditions (considering the range of TiO_2_ commonly found in each type of PCP) were estimated (Fig 2). Among all, over 84% (337 out of 401) of households have estimated releases of TiO_2_ through the use of PCPs. The majority of households TiO_2_-Ti release is estimated in the range of 0 to 20 milligrams (mg) Ti per day in the lower bound concentrations. When upper bound concentrations were used, daily household estimated release profiles derived from the inventory are shifted to be more normally distributed (skewed towards the left, S3 Fig in S1 File). 7.5% of households are predicted to elicit over 100 mg Ti per day, with the maximum household emission at 336 mg Ti per day.

**Fig 3:**
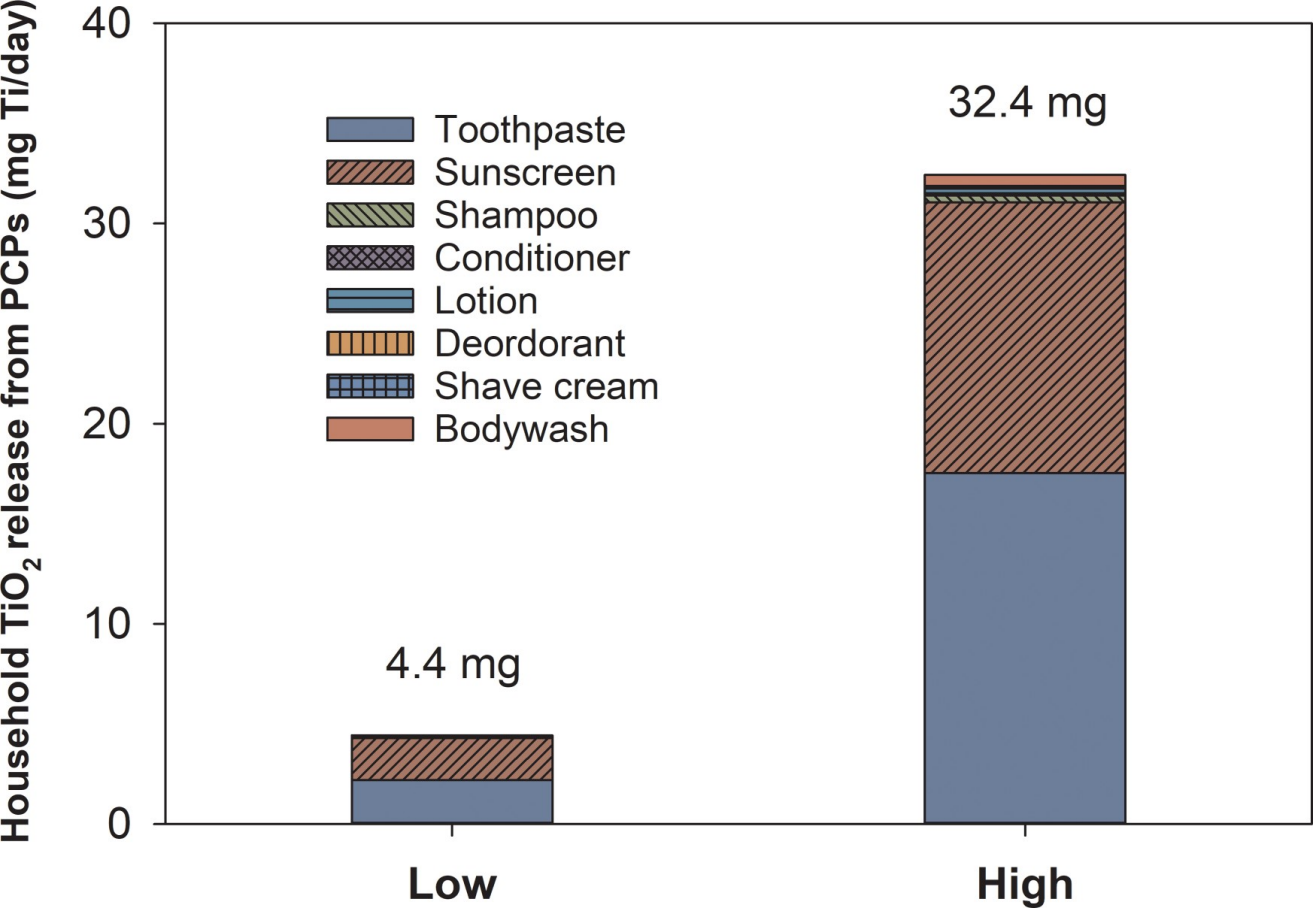
Predicted average concentrations (lower and upper bound) of TiO_2_-Ti released from households through PCP usage.

On average, the estimated daily household TiO_2_-Ti releases were 4.4 and 32.4 mg for lower and upper bound concentrations (Fig 3). Among all, the majority of TiO_2_-Ti was released from toothpaste and sunscreen, contributing 49% and 48% in the lower bound, 54% and 42% in the upper bound, respectively. The large contribution from sunscreen is due to the high concentration (previously quantified as 14–90 mg Ti/g of sunscreen [10]) of TiO_2_ in such products compared with other PCPs (S1 Table in S1 File). The other categories combined contributed an estimated 3% and 4% TiO_2_-Ti release in lower and upper bound, respectively.

### 3.3 Ti concentration quantified in WWTP samples

**Fig 4:**
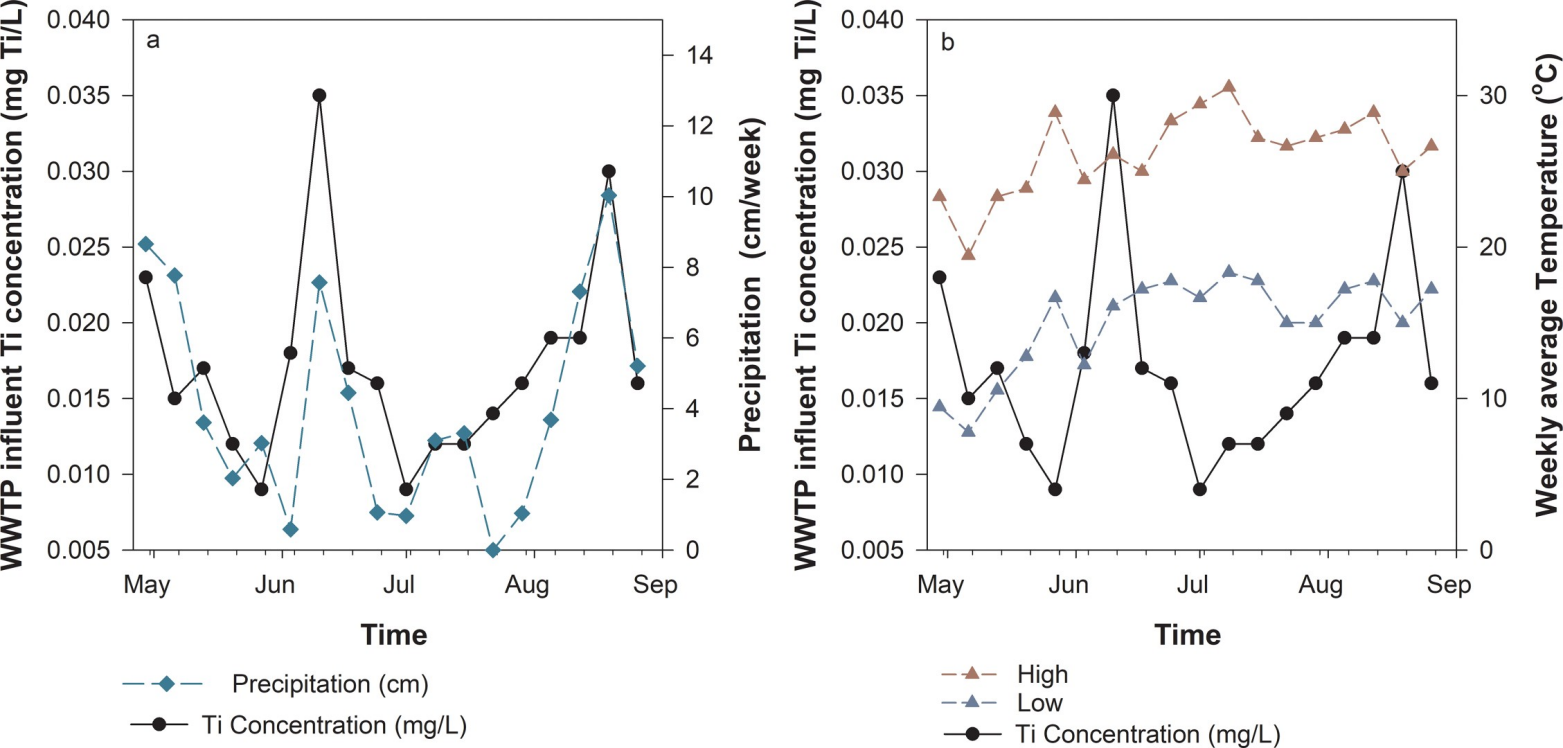
Ti concentration quantified in WWTP. (a) Ti concentrations versus the corresponding average precipitation and (b) low and high temperature collected from April 29^th^ to August 26^th^, 2018.

The total Ti concentrations in WWTP samples were quantified weekly from April 29^th^ to August 26^th^, 2018. The weekly average precipitation and temperature were plotted against the quantified Ti concentrations to show the potential correlations between these environmental parameters (Fig 4). The total quantified Ti in the WWTP influent water samples ranged from 0.009 to 0.035 mg Ti/L. The trend of Ti concentrations in wastewater follows the precipitation trend presented in Fig 4A. This suggests that urban run-off contributes greatly to the total Ti entering the WWTP. In addition, when the average temperatures were high (Fig 4B), relatively low and stable Ti concentrations in wastewater samples were observed in the month of July. Since sunscreen is estimated to be one of the major contributors for TiO_2_ emission, warm weather could lead to a potential wash-off of TiO_2_-Ti directly to lakes and rivers without going through the WWTP due to increased summer recreational activity in surface waters [37].

### 3.4 Materials flow through use and disposal

**Fig 5:**
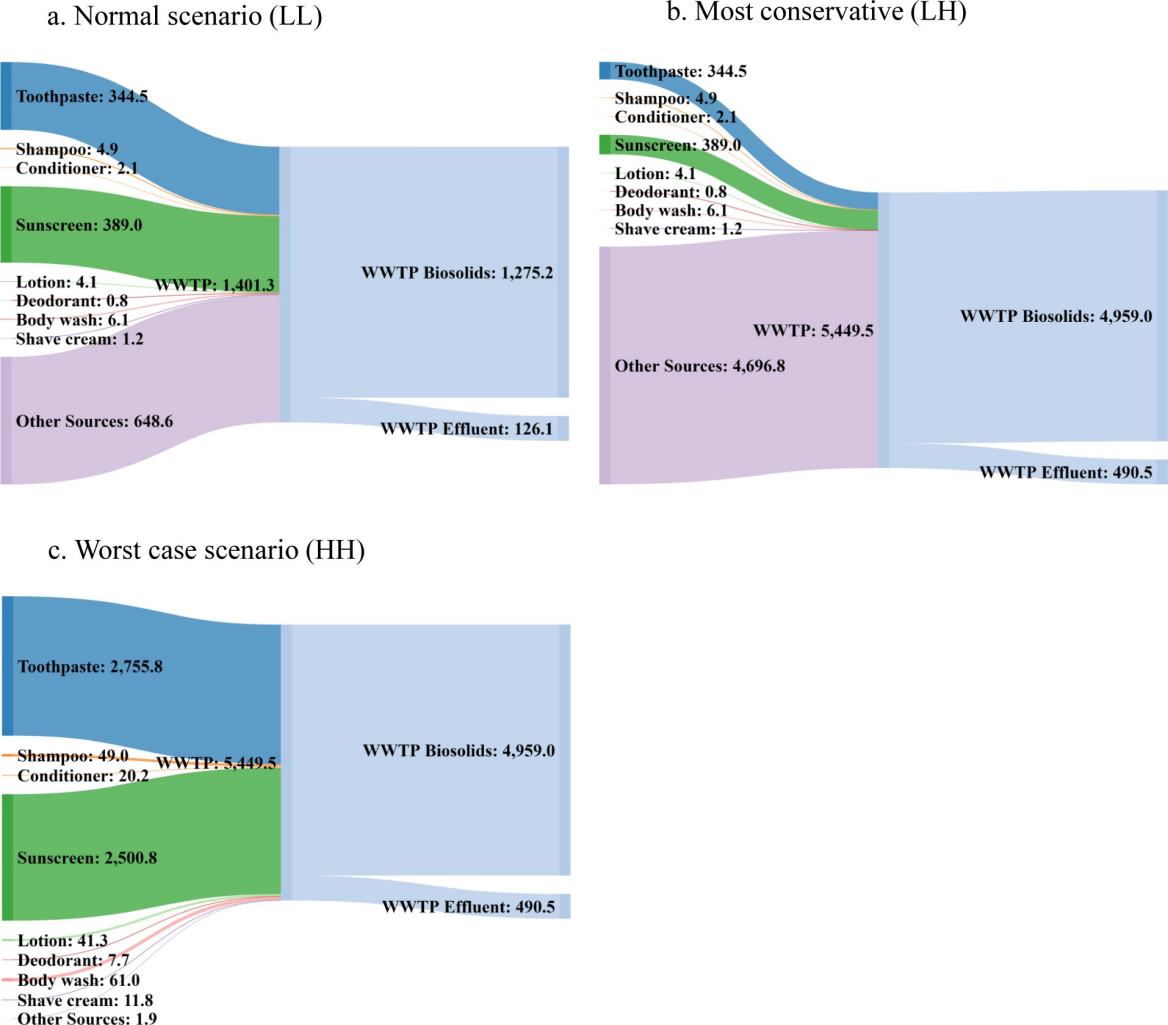
Estimated average material flow for TiO_2_-Ti (g/day) entering WWTP from each PCP category (demonstrated as g Ti/day). a) LL (normal scenario): estimated Ti released (lower bound) and lower end of detected total Ti in wastewater samples; b) LH (most conservative): estimated Ti released (lower bound) and higher end of detected Ti in wastewater samples; c) HH (worst case scenario): estimated Ti released (upper bound) and higher end of detected Ti in wastewater samples.

The regional WWTP considered in this study treats 41 million gallons (155 million liters) of wastewater and 45,000 pounds (22,412 kg) of biosolids daily on average. Based on the quantified total Ti in influent wastewater samples (Fig 4), the total average load to the WWTP ranges between 1401 and 5450 g Ti/day. Fig 5 provides a graphical representation of the estimated flow of Ti for PCPs from use, through the wastewater treatment process (WWTP), to release. Three different scenarios were considered: 1) the estimated lower bound values of Ti release from PCPs (shown in Fig 3) combined with the low end of detected total Ti concentration in wastewater samples (LL); b) the most conservative scenario where the estimated lower bound values of Ti release from PCPs paired with the high end of the detected Ti concentration in wastewater samples (LH); c) a worst case scenario where the estimated upper bound of Ti release from PCPs combined with the high end of the detected Ti concentration in wastewater samples (HH). Use of the upper bound Ti estimated concentrations from PCPs exceeded the value from the low end detected Ti concentration in wastewater samples, which was therefore neglected for further consideration. Results show the estimated lower and upper bound of Ti emitted to the WWTP from PCPs are in the range of 753 to 5448 g/day. Ti released through PCPs is estimated to account for 53.7% of total detected Ti in the influent wastewater in the LL scenario, whereas the LH and HH scenarios result in 13.8% and 99.9% contributions, respectively. Among the surveyed PCPs, toothpaste and sunscreen again accounted for the majority of the material flows, followed by bodywash, shampoo, lotion, and other products. By assuming the WWTP has a TiO_2_ removal efficiency of 91% [26], the average mass distribution of Ti to treated effluent and biosolids is 126 g and 1275 g, respectively, in the LL scenario. These estimated distributions change to 491 g and 4959 g of Ti to wastewater effluent and biosolid in the LH and HH scenarios.

### 3.5 Predicted TiO_2_ NP concentrations

**Fig 6:**
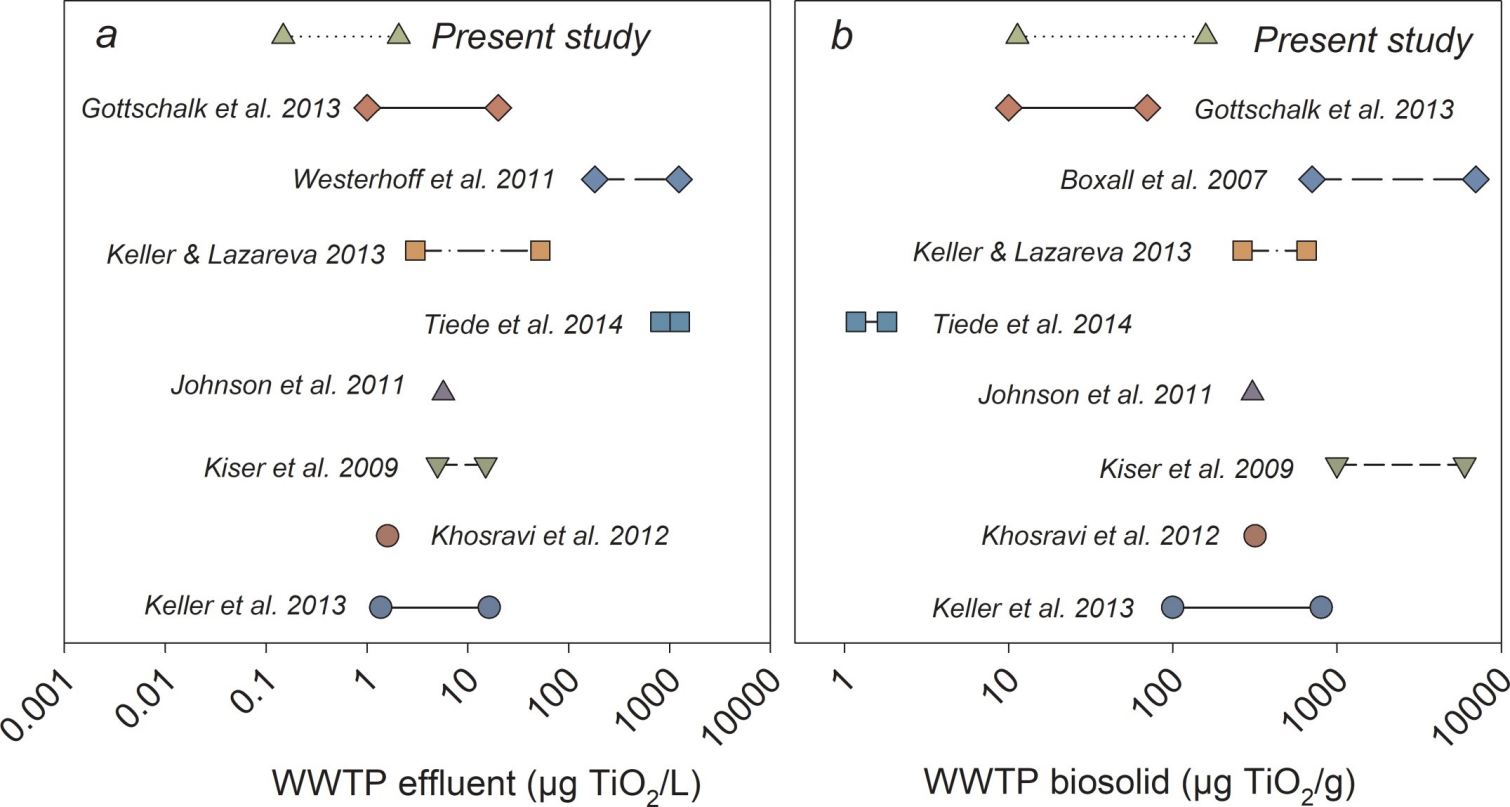
Comparison among current study with other published researches. The predicted TiO_2_ NP concentrations in the WWTP effluent (a) and biosolids (b) from present study and the literature [23, 24, 27, 28, 38–43]. Detailed values of the predicted data are presented in S3 Table in S1 File.

Literature suggests removal rates for NPs in WWTPs range from 75 to 97% if employing secondary treatment [26], and another study found a TiO_2_ NP removal rate nearly 96% in a simulated WWTP [23]. Therefore, two major assumptions were used to estimate the TiO_2_ NPs in the WWTP effluent and biosolids: 1) a 91% removal efficiency was assumed for the WWTP [26]; 2) 10% - 36% of the Ti quantified in the wastewater influent was assumed to come from TiO_2_ NPs [10–14]. Based on different scenarios presented in Fig 5, the estimated TiO_2_ NP concentrations range from 0.15 to 2.06 μg TiO_2_/L (0.09 to 1.24 μg Ti/L) in the WWTP effluent (Fig 6; S2 Table in S1 File). TiO_2_ NP in WWTP biosolids are estimated from 11.34 to 158.70 μg TiO_2_/g biosolids (6.8 to 95.2 μg Ti/g biosolids). Compared to other top-down analyses, the predicted TiO_2_ NP concentrations in this study are towards the lower end of reported ranges, mainly because only TiO_2_ NP released from PCPs were considered throughout the estimates.

### 3.6 Roundtable discussion

A roundtable discussion was held as part of this work to provide a two-way flow of information. Participants who completed the survey expressed interests and desires in learning more towards the use of NPs in PCPs. Common ideas that arose included the use of TiO_2_ in food products and the frequency with which it occurred. Participants identified other commonly used household items that could also contribute to TiO_2_ release, such as food, household cleaning supplies and paints. Participants also expressed interests about human risks of NP versus a non-NP formulated product. Their concern was whether TiO_2_ NP formulated PCPs would be harmful as it was absorbed dermally when used. In addition, hesitation was indicated towards purchasing NP formulation products because participants indicated limited information is currently available to the consumers. Another thought expressed was the use of “NP” as a possible buzz word. They expressed concern that labeling products as nano or non-nano may lead to consumers buying NP products because the word has a positive connotation and represents new, innovative technology, whereas the health and safety aspects are usually overlooked. In addition, participants contributed ideas for further research to quantify TiO_2_ released from consumers. One participant suggested an alternative method for modeling TiO_2_ release by using sales data based upon the amount of TiO_2_ products sold at stores within the area to identify the volume of TiO_2_ released into the WWTP. Participants also expressed a desire for a more complete and consumer accessible database detailing TiO_2_ quantities within products, which would help promote green consumerism by increasing public awareness of the TiO_2_ quantities within their everyday household items.

### 3.7 Environmental implications

Previously, research has utilized the TiO_2_ NP production volumes to generate a top down flow of TiO_2_ to different environmental compartments. This work provided a unique perspective in quantifying the amount of TiO_2_ entering the WWTP and the environment, utilizing a citizen science based bottom up approach. The TiO_2_ inventory was created by households from their adoption and usage of TiO_2_ containing PCPs (SI 2), which was then applied at a sewage shed level. This first community sourced inventory shows a wide range of applications for TiO_2_ being used in various PCPs. Overall, toothpaste and sunscreen are two primary sources of PCPs for TiO_2_ entering WWTPs. The majority of toothpaste contains TiO_2_ as an ingredient, whereas sunscreens generally have high TiO_2_ concentrations according to the literature but are used in lower total quantities than toothpaste. Even though more refined data could be achieved by quantitatively measuring TiO_2_ NPs presented in each of the PCP listed in the inventory, it is extremely labor, time, and cost intensive. This work also suggests that this approach could be valid for estimating other emerging contaminants in the waste water treatment stream.

The following discussion seeks to clearly state several major assumptions used in this study. The estimated TiO_2_ NP concentrations are largely relying on the literature data collected due to technical difficulties of quantifying NPs in all PCPs. In the study, TiO_2_ NP concentrations predicted in WWTP effluent and biosolid fit in the lower range compared to other top down analyses (Fig 5). This is mainly due to the fact that other sources of TiO_2_ NPs may play a role in the overall estimates. One study conducted by Keller et al. estimated TiO_2_ NP released per person ranges from 1.95−22.70 mg/day, depending on the location of the focus area [26]. In comparison, the estimated personal release through PCP usage containing both bulk and nanoscale ranges from 2.91 to 21.50 mg TiO_2_/day (1.75 to 12.90 mg Ti/day) in present study (S3 Fig in S1 File), but only up to 36% of the predicted values are estimated to be present as NPs (1.05 to 7.75 mg TiO_2_ NP/day), which is lower than what Keller et al. predicted. Depending on the measured total Ti in WWTP and estimated household releasing profile, Ti released from household PCPs contributes at least 14% total Ti measure at the WWTP (Fig 5), supporting the hypothesis that other sources (such as food and other household consumer products) contribute greatly to the total Ti released. Several studies showed that TiO_2_ NPs are commonly added as food additives in cheeses, sauces, and beverages [44], and their nano-form have been isolated from products such as chewing gum [45]. Additionally, Windler et al. identified small amount of TiO_2_ NP release from textile laundering other than food sources [46]. Furthermore, the trend of Ti concentration quantified in the WWTP influent correlates with temporal precipitation data (Fig 4A), where higher amounts of Ti were measured in wastewater samples when more precipitation occurred. This may potentially be explained by leaching from construction materials such as building blocks and outdoor windows, which may contain TiO_2_ NPs with photocatalytic reactivity properties to achieve “self-cleaning” features. A previous study demonstrated that the emission of TiO_2_ NP leaching from these products occurred by wear and weather 7 months after application [47]. This suggests that urban run-off is also a major contributor for Ti to be released to the WWTP. Therefore, in accordance with the roundtable participants’ interests and concerns, identifying the sources in food and other consumer products, route of releases, and related health impacts are major research questions that should be addressed in future work.

Research indicates the dominating emission pathway of TiO_2_ NP occurs via WWTP [27, 48], and the flow of TiO_2_ NP within and after WWTP is largely dependent on how the TiO_2_ partitions to the solid and liquid phases of wastewater. Up to 85% of total TiO_2_ NP emissions flow through WWTP, but only a small portion of TiO_2_ NPs (up to 33% of the influent wastewater) are emitted to the freshwater though WWTP effluent [49]. Several studies have found the high ionic strength of wastewater significantly enhances the aggregation tendency of NPs [50, 51], which forms larger particulates and increases the chance of being accumulated in the sewage sludge. Approximately 36% of inflow TiO_2_ NP accumulated in sewage sludge during wastewater treatment is ultimately applied onto soils in many countries, and a lower portion (30%) is deposited into landfills either by direct landfilling of biosolids or after biosolids incineration [49]. In addition, due to the relative inert nature compared to other soluble metal/metal oxide NPs, TiO_2_ NPs could also be recycled within the activated sludge at WWTPs, resulting in accumulation within biosolids. Research has suggested TiO_2_ NPs in WWTP sludge can reach up to 211 mg Ti/g [52], which can potentially impact the treatment efficiency, such as flocculation stability of activated sludge flocs [53], decrease nitrification and bacterial community shift [54], etc., leading to further problems in terms of treatment plant functions. Thus, understanding NP fate and estimating NP concentrations in a WWTP setting can also help identify and prevent potential drawbacks caused by NP accumulations.

One aspect not captured in this study is that TiO_2_ and TiO_2_ NPs could also be emitted from PCP to local freshwater systems without going through a WWTP. No strong correlation between the average temperature versus the Ti in wastewater samples was found in current research, however, sunscreen has been identified previously as a source for TiO_2_ NPs that can enter surface waters directly. A study conducted in Spain estimated that tourism activities during a summer day may release on the order of 4 kg of TiO_2_ NPs to Mediterranean coastal waters [37]. In another study, an increase of TiO_2_ particles released from sunscreens was identified during the summer season in year-round monitoring in a lake near Vienna, Austria [55]. Thus, release of TiO_2_ and TiO_2_ NP to freshwater ecosystems directly through swimming and other aquatic recreational activities, could possibly underestimate the TiO_2_ concentrations predicted in this study.

The current citizen science based analysis presents a unique interdisciplinary perspective on estimating the TiO_2_/TiO_2_ NP entering the environment at household level, while providing feedback to the community where the information was gathered, and gaining further insights as to the concerns of citizen scientists and direction of future research. This research also developed the first community sourced TiO_2_ containing PCP inventory to share with the public, regulators, and the industry. This study provides evidence that bottom up and citizen science based approaches may be a valid way to estimate the quantity of emerging contaminates in WWTPs.

## Supporting information

S1 File (DOCX)

S2 File (PDF)
