# Supplementary material for: A citizen science approach estimating titanium dioxide released from personal care products
Source: PLoS One. 2020 Jul 29;15(7):e0235988. doi: 10.1371/journal.pone.0235988 (PMC7390335; doi:10.1371/journal.pone.0235988)
Supplement: S1 File — (DOCX) [file pone.0235988.s001.docx]

**Supplemental Information 1**

**A citizen science approach estimating titanium dioxide released from personal care products**

*Fan Wu ^a,b^, Matt Seib ^c^, Samantha Mauel ^b^, Sydney Klinzing ^b^, Andrea L. Hicks ^b*^*

^a^ School of Environment and Guangdong Key Laboratory of Environmental Pollution and Health, Jinan University, Guangzhou, China

^b^ Department of Civil and Environmental Engineering, University of Wisconsin-Madison, Madison, WI, USA

^c^ Madison Metropolitan Sewerage District, Madison, WI, USA

^*^ Corresponding author. E-mail: [*Hicks5@wisc.edu*](mailto:Hicks5@wisc.edu)

**Survey Results**

An IRB approved social survey was distributed to the Madison Metropolitan community under the collaboration wastewater swage district service area to collect information on their daily used PCPs. In total, over a thousand survey responses were received from April 6, 2018 to December 21, 2018, whereas 401 valid survey responses within the service district was used and analyzed. The total valid survey consists nearly 0.27 % of the total Madison Metropolitan households. Among the valid responses, 279 respondents (69.6%) were female and 120 respondents (29.9%) were male (0.5% identified as other). The Dane county household income for 2016 was used to correct the final data collected to estimate the release of TiO_2_ entering WWTP (Figure S2).


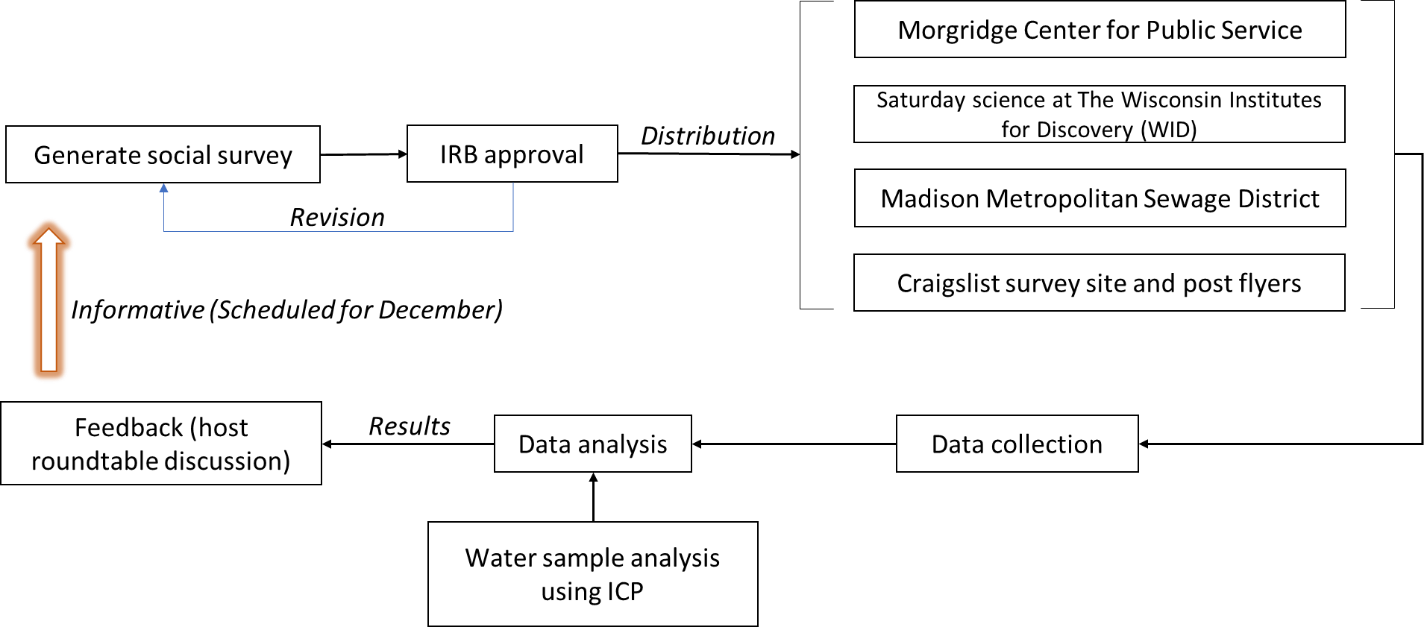


*Figure S1. Systematic flow of current research project.*


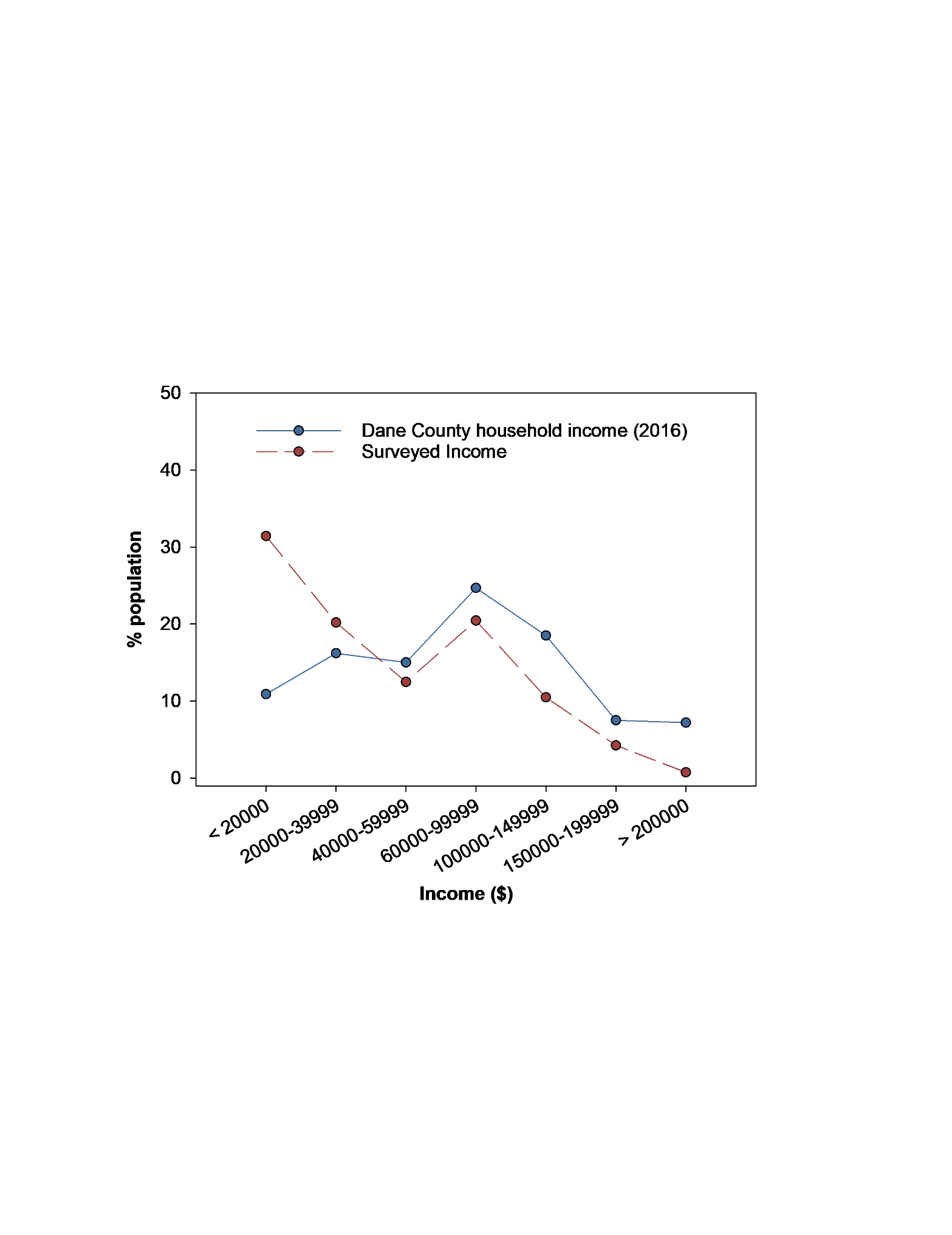


*Figure S2. Distributional comparison between the household income from collected surveys and the Dane county household income in 2016.*


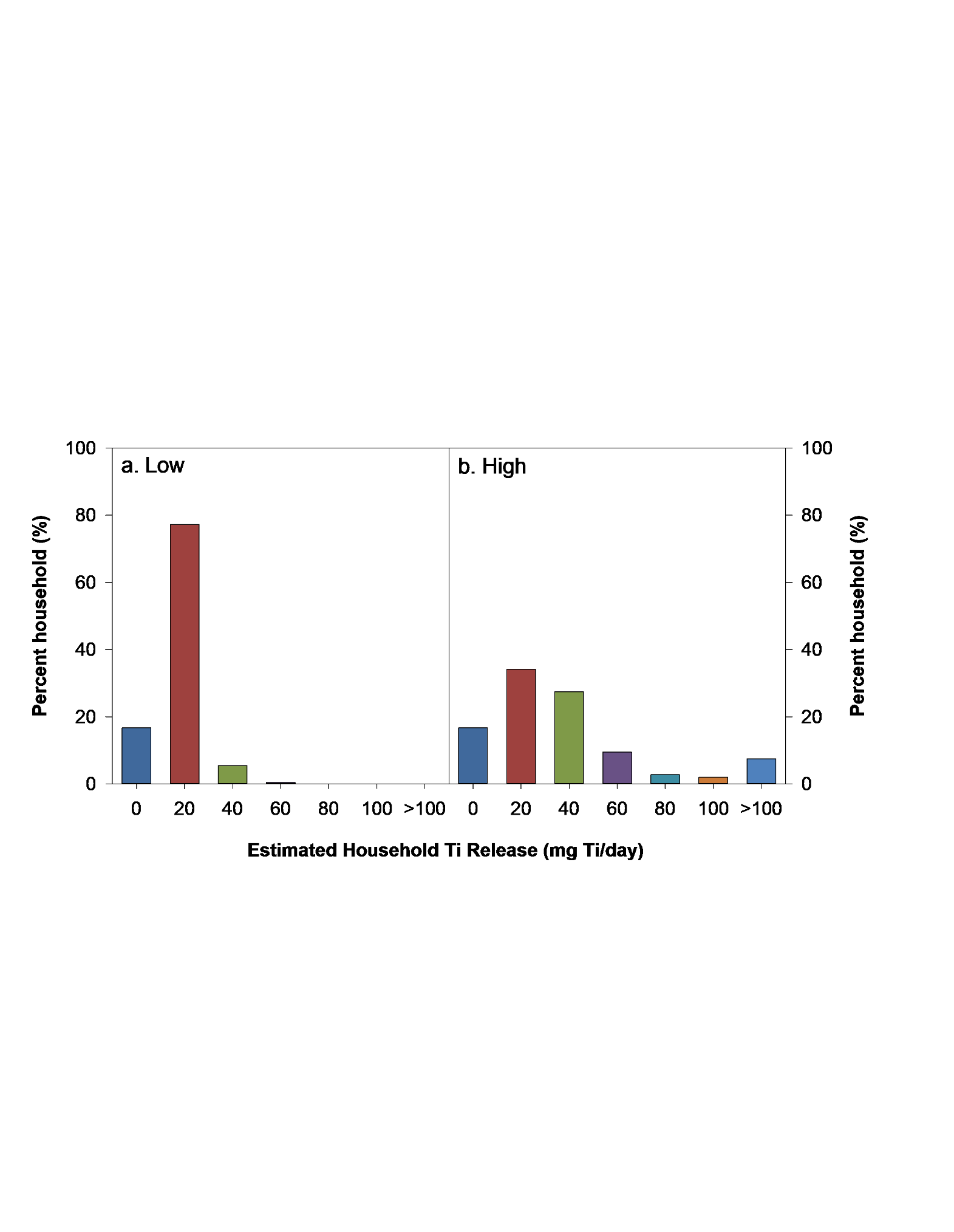


*Figure S3. Distribution of daily TiO_2_-Ti release profile from the household PCPs based on the estimated lower and upper Ti concentrations quantified in PCPs.*


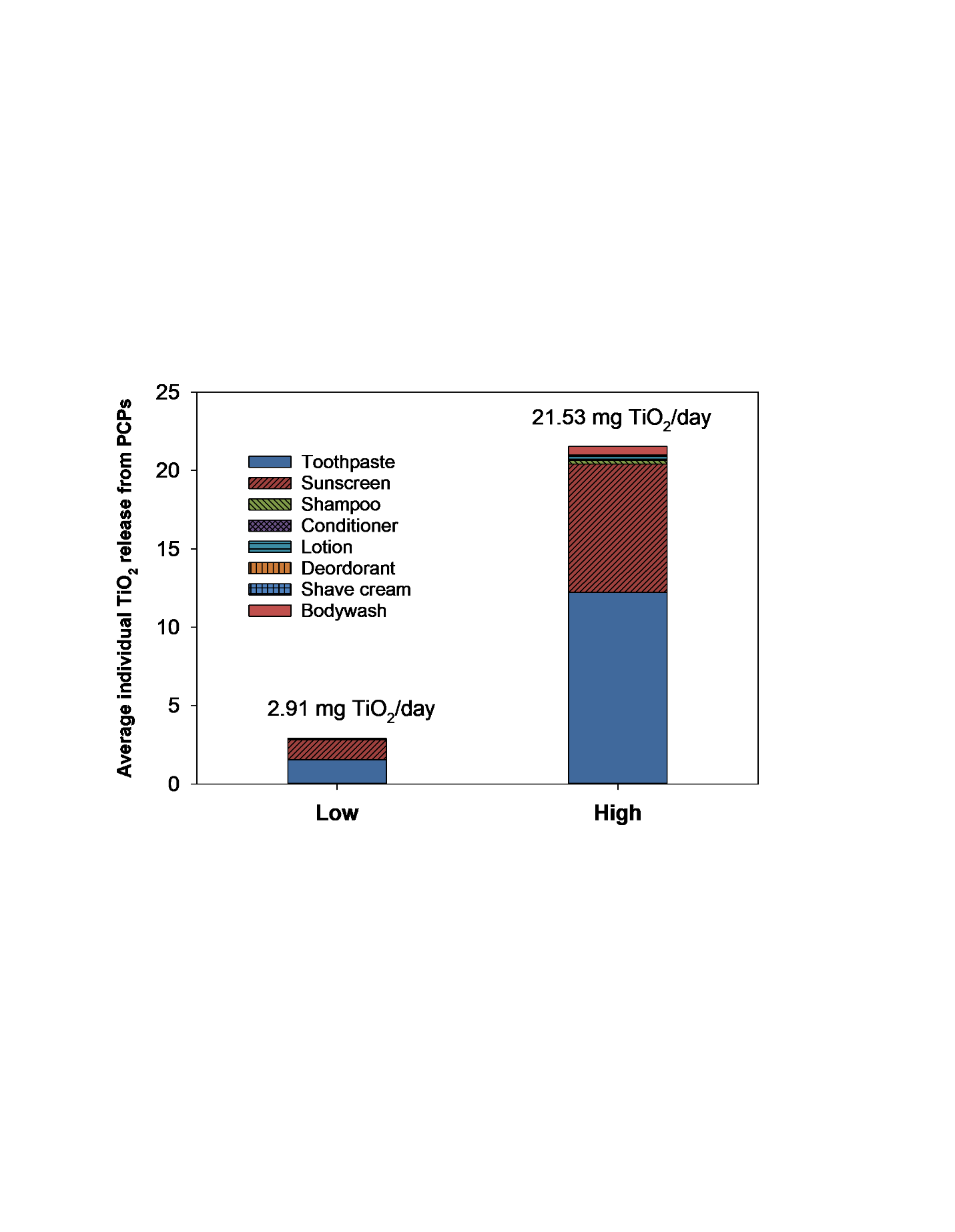


*Figure S4. Predicted average low and high concentrations of TiO_2_ released from individuals through using PCPs.*

Table S1. Data used to calculate the concentration of TiO_2_ used in each PCP category.


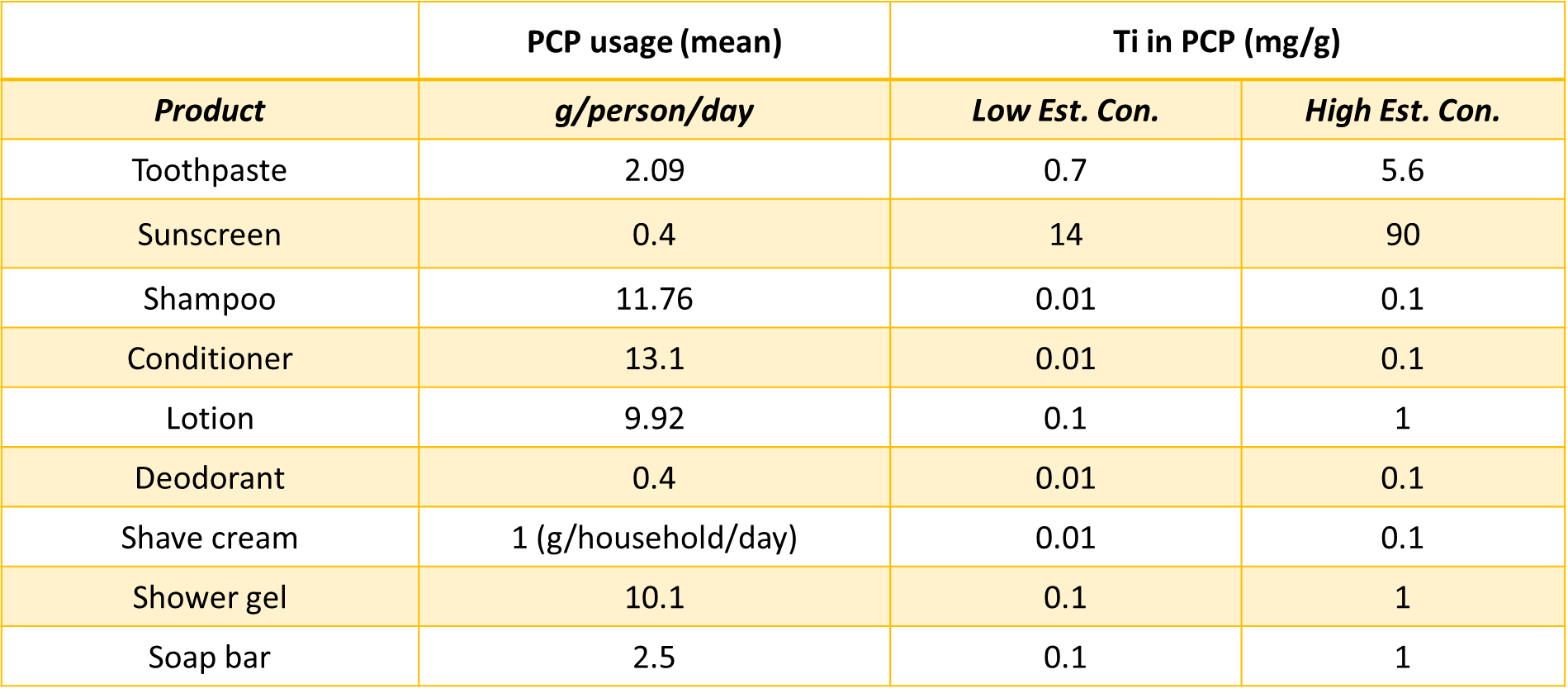


Table S1 summaries the concentrations of TiO_2_/TiO_2_ NP detected in various PCPs from a suite of research articles [1-5]. Toothpaste and sunscreen have high concentration ranges, whereas other products show a possible range, with some are identified in the range of 0.1% to 1%. Thus, lower and upper bound was estimated using the concentration compiled from multiple studies. In addition, research investigated the personal usage patterns of PCPs. The average usage patterns were used to perform calculations in the present study.

Table S2. The estimated TiO_2_ NP concentrations based on different scenarios.

| Assumptions | | WWTP effluent | Biosolid |
| --- | --- | --- | --- |
|  |  | µg TiO_2_/L | µg TiO_2_/g |
| 10% nano | Lower bound | 0.147 | 11.335 |
|  | Upper bound | 0.573 | 44.080 |
| 36% nano | Lower bound | 0.530 | 40.806 |
|  | Upper bound | 2.062 | 158.689 |

Table S3. Summarized TiO_2_ NP concentrations predicted in WWTP effluent and biosolids.

| Ref. | WWTP effluent (µg TiO_2_/L) | Biosolids  (µg TiO_2_/g) | Comments |
| --- | --- | --- | --- |
| (Keller, McFerran et al. 2013) [6] | 1.37-16.3 | 100 - 802 | 15% to 85% quantiles |
| (Keller and Lazareva 2013) [7] | ~ | 266 - 652 |  |
| (Musee 2011) [8] | 6.9×10-3 - 4.36 | ~ | Min to Max |
| (Westerhoff, Song et al. 2011) [9] | 181 - 1233 (median: 321) | ~ |  |
| (Khosravi, Hoque et al. 2012) [10] | 1.6 | 317.4 |  |
| (Kiser, Westerhoff et al. 2009) (*) [11] | <5 - 15 | 1000 - 6000 |  |
| (Johnson, Bowes et al. 2011) [12] | 5.7 | 305 | Measured avg. |
| (Tiede, Westerhoff et al. 2012) [13] | 809 - 1260 | 1.17 - 1.81 (assume 207.5 kg/m3 as sludge density) | (Est. from Sunscreen - all product, assuming 23% removal efficiency) |
| (Boxall, Chaudhry et al. 2007) [14] | ~ | 701-7007 |  |

**Validation criteria:**

1. Citizens reside outside the Madison Metropolitan Sewerage District are excluded from this study. The full list of the district includes: city of Madison, Fitchburg, Middleton, Monona, Verona; town of Dunn - Kegonsa, Dunn, Pleasant Springs, Verona - Marty Farms, Verona, Westport; village of Cottage Grove, Dane, DeForest, Maple Bluff, McFarland, Shorewood Hills, Waunakee, and Windsor.

2. Citizens who live in Madison Metropolitan Sewerage District but not provided with city sewerage service (e.g. having own septic tank) are excluded in the study.

3. Participants will have to ensure read the informed consent to be able to continue the survey, otherwise the survey will not be able to be completed.

4. Incomplete survey will not be included in final data analysis, and the GeoIP location was set to target the population at WWTP service area.

**Survey Instrument**

***Informed Consent***

**UNIVERSITY OF WISCONSIN-MADISON**
**Research Participant Information and Consent Form**

**Title of the Study:**Titanium dioxide (TiO_2_) down the drain - personal care products in the wastewater stream

**Principal Investigator:** Andrea L. Hicks (phone: 608-262-1262) (email: hicks5@wisc.edu)

DESCRIPTION OF THE RESEARCH
You are invited to participate in a research study about titanium dioxide in personal care products. You are eligible to participate if you reside in the service area of the Madison Metropolitan Sewerage District (MMSD) - although this work is not sponsored by the MMSD.

The purpose of the research is to create a citizen science inventory of the quantity of titanium dioxide entering the waste water treatment stream from consumer personal care products. This study will include a survey. The research will be conducted online and in hard copies of the survey instrument. 

WHAT WILL MY PARTICIPATION INVOLVE? 
If you decide to participate in this research you will be asked to complete a survey about your personal care products (i.e. toothpaste, sunscreen), and whether they contain titanium dioxide. You will be asked to complete 1 survey, and your participation will last approximately 15 minutes. 

ARE THERE ANY RISKS TO ME? 
No risk is expected from participation in this research. 

ARE THERE ANY BENEFITS TO ME? 
There are no direct benefits. However, you will be helping to further science.  

WILL I BE COMPENSATED FOR MY PARTICIPATION? 
You will receive $5 in the form of an Amazon.com gift code for participating in this study. If you do withdraw prior to the end of the study, you will receive $0. 

HOW WILL MY CONFIDENTIALITY BE PROTECTED? 
While there will probably be publications as a result of this study, your name will not be used. Only group characteristics will be published. No personally identifiable data will be shared with the Madison Metropolitan Sewerage District. 

WHOM SHOULD I CONTACT IF I HAVE QUESTIONS? 
You may ask any questions about the research at any time. If you have questions about the research after you leave today, you should contact the Principal Investigator Andrea L. Hicks at 608-262-1262. 

If you are not satisfied with response of research team, have more questions, or want to talk with someone about your rights as a research participant, you should contact the Education and Social/Behavioral Science IRB Office at 608-263- 2320. 

Your participation is completely voluntary. If you decide not to participate or to withdraw from the study, it will have no effect on any services you are currently receiving. 

By completing this survey, you indicate that you have read this consent, had an opportunity to ask any questions about your participation in this research, voluntarily consent to participate, and agree to be contacted for future phases of the research. You may save a copy of this form for your records.

- Yes, I have read the consent form and give my consent to participate in this study.

**Survey**

Introduction

Thank you for working with us as a citizen scientist. As a citizen scientist, we need your help to identify products you use that may contain titanium dioxide (also known as TiO_2_).

Titanium dioxide is a common ingredient in food and personal care products (such as shampoo and toothpaste). We are working to identify how much titanium dioxide is entering the wastewater treatment stream, at a household level. By answering the following survey, you are contributing to science, by telling us about the products in your home.

The survey is about products that will ultimately enter the wastewater stream and be treated by your local wastewater treatment plant. An example of a product with the ingredient label and titanium dioxide is presented below:


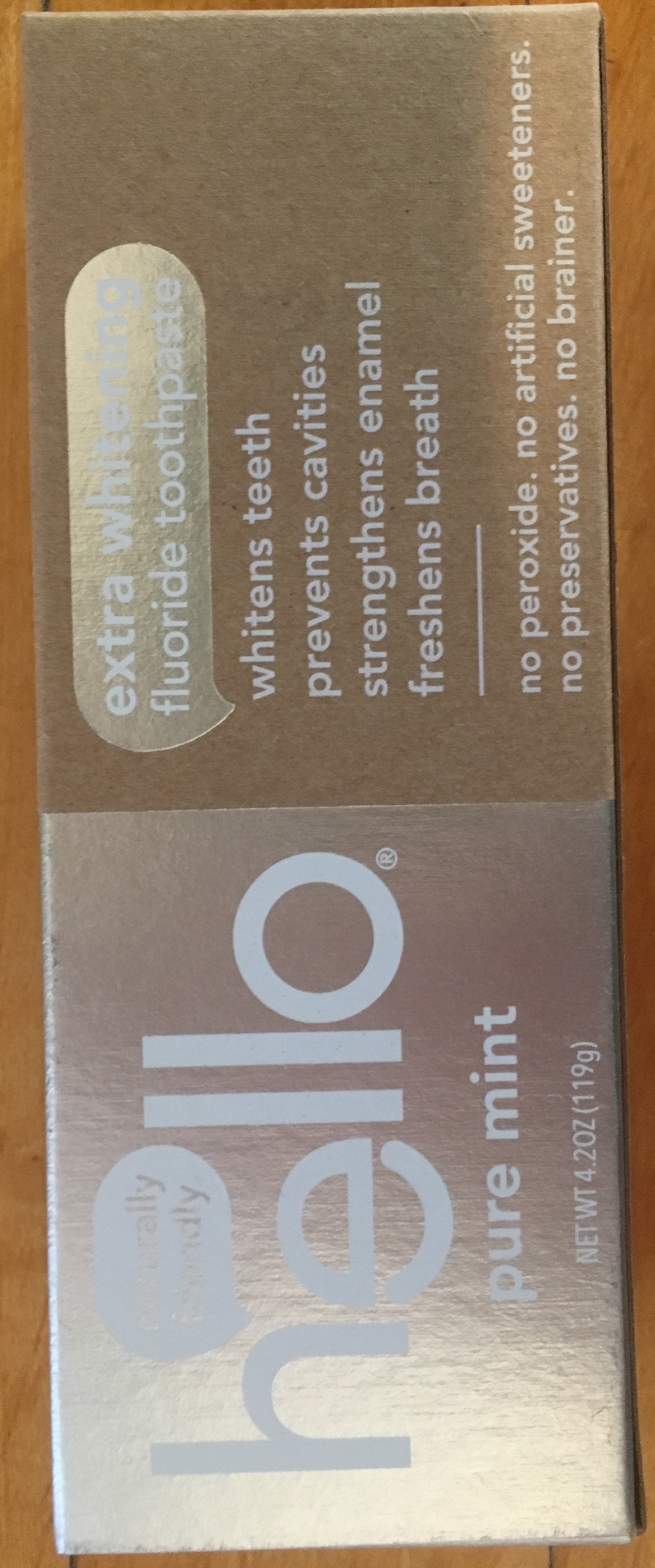

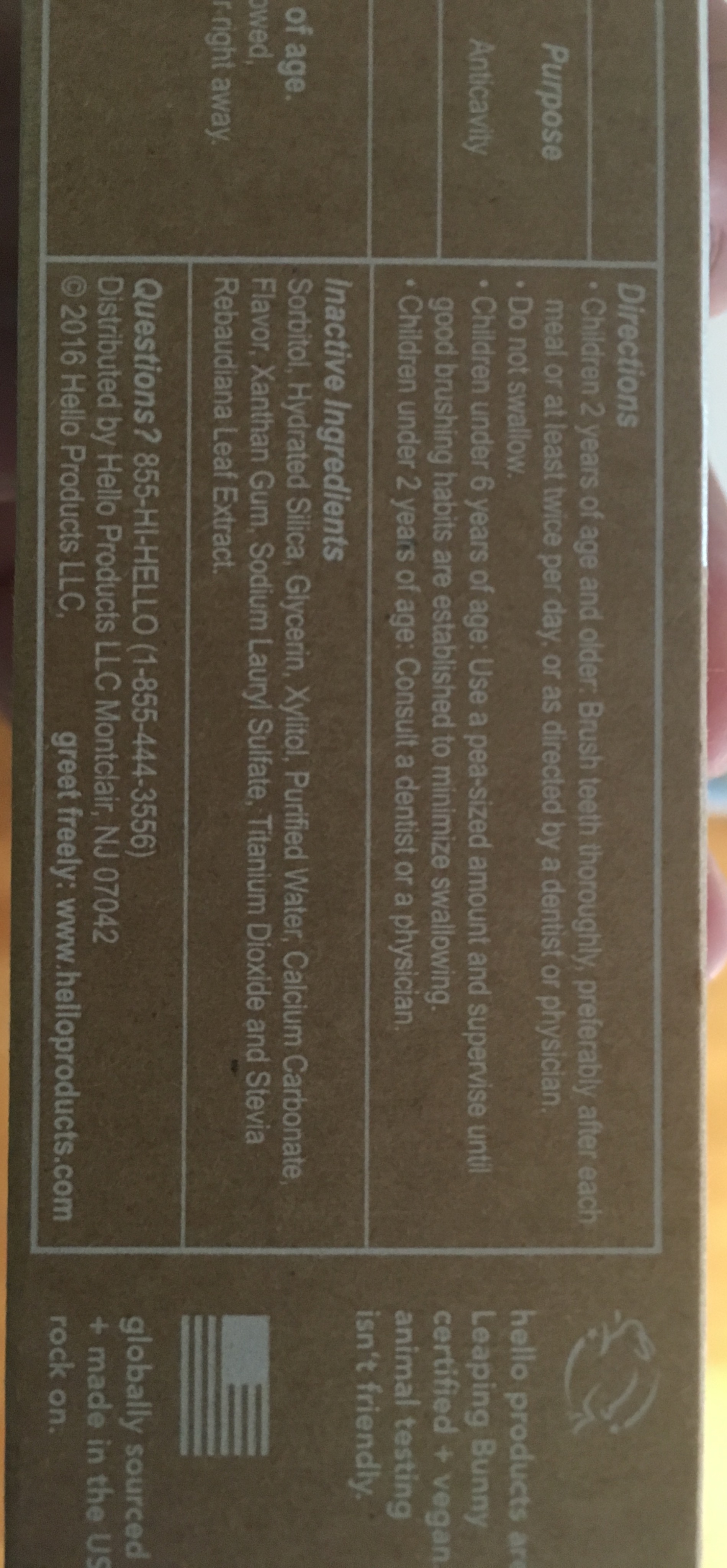


a)

b)

**Survey questions**

***Region***

Q1. Please select the city/town/village you are currently residing:

- City of Fitchburg
- City of Madison
- City of Middleton
- City of Monona
- City of Verona
- Town of Dunn - Kegonsa
- Town of Dunn
- Town of Pleasant Springs
- Town of Verona - Marty Farms
- Town of Verona
- Town of Westport
- Village of Cottage Grove
- Village of Dane
- Village of DeForest
- Village of Maple Bluff
- Village of McFarland
- Village of Shorewood Hills
- Village of Waunakee
- Village of Windsor
- I am not from any of the region listed above

Q2. Is your residence connected to sewer or a septic tank?

- Sewer
- Septic tank
- I don’t know

***Toothpaste***

Q3. Currently how many tubes of toothpaste is your household using? (Enter number)?

(numerical entry box)

Q4. What are the names and brands of the toothpaste?

1 (text entry box)

2 (text entry box)

3 (text entry box)

4 (text entry box)

5 (text entry box)

6 (text entry box)

Q5. Do any of the tubes list titanium dioxide on their ingredient list?

(select one)

- Yes
- No
- I don’t know

If yes

Q6. Place a check mark next to the products that contain titanium dioxide.

(list will be generated by Qualtrics based on answer to question 2)

***Shampoo***

Q7. Currently how many bottles/ containers of shampoo is your household using?

(numerical entry box)

Q8. What are the names and brands of the shampoo?

1 (text entry box)

2 (text entry box)

3 (text entry box)

4 (text entry box)

5 (text entry box)

6 (text entry box)

Q9. Do any of the containers list titanium dioxide on their ingredient list?

(select one)

- Yes
- No
- I don’t know

If yes

Q10. Place a check mark next to the products that contain titanium dioxide.

(list will be generated by Qualtrics based on answer to question 6)

***Conditioner***

Q11. Currently how many bottles/ containers of conditioner is your household using?

(numerical entry box)

Q12. What are the names and brands of the conditioner?

1 (text entry box)

2 (text entry box)

3 (text entry box)

4 (text entry box)

5 (text entry box)

6 (text entry box)

Q1311. Do any of the containers list titanium dioxide on their ingredient list?

(select one)

- Yes
- No
- I don’t know

If yes

Q14. Place a check mark next to the products that contain titanium dioxide.

(list will be generated by Qualtrics based on answer to question 10)

***Lotion / Skin cream***

Q15. Currently how many bottles/ containers of lotion or skin cream is your household using?

(numerical entry box)

Q16. What are the names and brands of the lotion or skin cream?

1 (text entry box)

2 (text entry box)

3 (text entry box)

4 (text entry box)

5 (text entry box)

6 (text entry box)

Q17. Do any of the containers list titanium dioxide on their ingredient list?

(select one)

- Yes
- No
- I don’t know

If yes

Q18. Place a check mark next to the products that contain titanium dioxide.

(list will be generated by Qualtrics based on answer to question 14)

***Sunblock/ Sunscreen***

Q19. Currently how many tubes/ bottles of sunblock or sunscreen is your household using?

(numerical entry box)

Q20. What are the names and brands of the sunblock or sunscreen?

1 (text entry box)

2 (text entry box)

3 (text entry box)

4 (text entry box)

5 (text entry box)

6 (text entry box)

Q21. Do any of the containers list titanium dioxide on their ingredient list?

(select one)

- Yes
- No
- I don’t know

If yes

Q22. Place a check mark next to the products that contain titanium dioxide.

(list will be generated by Qualtrics based on answer to question 18)

If yes (Q21)

Q23. Please select the season you apply sunblock/sunscreen products

- Summer only
- All seasons

***Deodorant/ Antiperspirant***

Q24. Currently how many containers of deodorant or antiperspirant is your household using?

(numerical entry box)

Q25. What are the names and brands of the deodorant or antiperspirant?

1 (text entry box)

2 (text entry box)

3 (text entry box)

4 (text entry box)

5 (text entry box)

6 (text entry box)

Q26. Do any of the containers list titanium dioxide on their ingredient list?

(select one)

- Yes
- No
- I don’t know

If yes

Q27. Place a check mark next to the products that contain titanium dioxide.

(list will be generated by Qualtrics based on answer to question 22)

***Shaving Cream***

Q28. Currently how many containers of shaving cream is your household using?

(numerical entry box)

Q29. What are the names and brands of the shaving cream?

1 (text entry box)

2 (text entry box)

3 (text entry box)

4 (text entry box)

5 (text entry box)

6 (text entry box)

Q30. Do any of the containers list titanium dioxide on their ingredient list?

(select one)

- Yes
- No
- I don’t know

If yes

Q31. Place a check mark next to the products that contain titanium dioxide.

(list will be generated by Qualtrics based on answer to question 26)

***Other***

Q32. This is chance to list other personal care products that your household uses regularly that we haven’t asked about. What else is your household currently using?

1 (text entry box)

2 (text entry box)

3 (text entry box)

4 (text entry box)

5 (text entry box)

6 (text entry box)

7 (text entry box)

8 (text entry box)

Q33. Do any of the containers list titanium dioxide on their ingredient list?

(select one)

- Yes
- No
- I don’t know

If yes

Q34. Place a check mark next to the products that contain titanium dioxide.

(list will be generated by Qualtrics based on answer to question 29)

***Demographic Questions***

Q35. Which of the following genders do you most identify with?

(select one)

- Male
- Female
- Other

Q36. Which of the following age ranges best describes you?

(select one)

- 18 to 24
- 25 to 34
- 35 to 44
- 45 to 54
- 55 to 64
- 65 years and over

Q37. Which range best describes your household income level?

(select one)

- Less than $20,000
- $20,000 to $39,999
- $40,000 to $59,999
- $60,000 to $99,999
- $100,000 to $149,999
- $150,000 to $199,999
- $200,000 or more
- Prefer not to answer

Q38. Which of the options below best describes your marital status?

(select one)

- Never married
- No married (except separated)
- Separated
- Widowed
- Divorced

Q39. Do you identify as Hispanic?

- Yes
- No

Q40. Which of the following races best describes you?

- White alone
- Black or African American alone
- American Indian or Alaskan native alone
- Asian alone
- Native Hawaiian or other Pacific Islander alone
- Some other race alone
- Two or more races

Q41. How many people currently reside in your household?

(select one)

- 1
- 2
- 3
- 4
- 5
- 6
- 7
- 8
- 9+

***Contact Information***

Q42. Would you like to be contacted in order to receive your survey incentive?

- Yes
- No

Q43. If yes, enter email address:

(text answer box)

Q44. Would you like to be contacted in the future to participate in a roundtable discussion regarding the findings of this work?

- Yes
- No

Q45. If yes, enter email address:

(text answer box)

**Reference**

1. Weir A, Westerhoff P, Fabricius L, Hristovski K, Von Goetz N. Titanium dioxide nanoparticles in food and personal care products. Environmental Science & Technology, 2012. 46(4). 2242-2250.

2. Peters RJ, van Bemmel G, Herrera-Rivera Z, Helsper HP, Marvin HJ, Weigel S, Tromp PC, Oomen AG, Rietveld AG, Bouwmeester H. Characterization of titanium dioxide nanoparticles in food products: analytical methods to define nanoparticles. Journal of Agricultural and Food Chemistry, 2014. 62(27). 6285-6293.

3. Yang Y, Doudrick K, Bi X, Hristovski K, Herckes P, Westerhoff P, Kaegi R. Characterization of food-grade titanium dioxide: the presence of nanosized particles. Environmental Science & Technology, 2014. 48(11). 6391-6400.

4. Warheit, D, Brown S, and Donner E. Acute and subchronic oral toxicity studies in rats with nanoscale and pigment grade titanium dioxide particles. Food and Chemical Toxicology, 2015. 84. 208-224.

5. Rompelberg C, Heringa MB, van Donkersgoed G, Drijvers J, Roos A, Westenbrink S, Peters R, van Bemmel G, Brand W, Oomen AG. Oral intake of added titanium dioxide and its nanofraction from food products, food supplements and toothpaste by the Dutch population. Nanotoxicology, 2016. 10(10). 1404-1414.

6. Keller, AA, McFerran S, Lazareva A., Suh S. Global life cycle releases of engineered nanomaterials. Journal of Nanoparticle Research, 2013. 15(6). 1692.

7. Keller AA, Lazareva A. Predicted Releases of Engineered Nanomaterials: From Global to Regional to Local. Environmental Science & Technology Letter, 2014. 1(1). 65–70.

8. Musee N. Simulated environmental risk estimation of engineered nanomaterials: A case of cosmetics in Johannesburg City. Human & Experimental Toxicology, 2011. 30(9). 1181–95.

9. Westerhoff P, Song G, Hristovski K, A. Kiser M. Occurrence and removal of titanium at full scale wastewater treatment plants: implications for TiO_2_ nanomaterials. Journal of Environmental Monitoring, 2011. 13(5). 1195–203.

10. Khosravi K, Hoque ME, Dimock B, Hintelmann H, Metcalfe CD. A novel approach for determining total titanium from titanium dioxide nanoparticles suspended in water and biosolids by digestion with ammonium persulfate. Analytica Chimica Acta, 2012. 713. 86–91.

11. Kiser MA, Westerhoff P, Benn T, Wang Y, Pérez-Rivera J, Hristovski K. Titanium Nanomaterial Removal and Release from Wastewater Treatment Plants. Environmental Science & Technology, 2009. 43(17). 6757–63.

12. Johnson AC, Bowes MJ, Crossley A, Jarvie HP, Jurkschat K, Jürgens MD, Lawlor AJ, Park B, Rowland P, Spurgeon D, Svendsen C, Thompson IP, Barnes RJ, Williams RJ, Xu N. An assessment of the fate, behaviour and environmental risk associated with sunscreen TiO2 nanoparticles in UK field scenarios. Science of The Total Environment, 2011. 409(13). 2503–10.

13. Tiede K, Westerhoff P, Hansen SF, Fern GJ, Hankin SM, Aitken RJ, Chaudhry Q, Boxall ABA. Review of the Risks Posed to Drinking Water by Man-Made Nanoparticels. 2012.

14. Boxall ABA, Chaudhry Q, Ardern-Jones A, Jefferson B, Watts CD, Boxall ABA, Sinclair C, Baxter-Jones AD, Aitken R, Watts C, Chaudrhry Q. Current and Future Predicted Environmental Exposure to Engineered Nanoparticles. Science of the Total Environment, 2007. 390. 396–409.
