# Supplementary material for: A citizen science approach estimating titanium dioxide released from personal care products
Source: PLoS One. 2020 Jul 29;15(7):e0235988. doi: 10.1371/journal.pone.0235988 (PMC7390335; doi:10.1371/journal.pone.0235988)
Supplement: S2 File — (PDF) [file pone.0235988.s002.pdf]

Supplemental Information 2

A citizen science approach estimating titanium dioxide released from personal care products

Fan Wu [a,b], *Matt Seib* [c], *Samantha Mauel* [a], *Sydney Klinzing* [a], *Andrea L. Hicks* [a*]

[a] School of Environment and Guangdong Key Laboratory of Environmental Pollution and Health, Jinan University, Guangzhou, China

[b] Department of Civil and Environmental Engineering, University of Wisconsin-Madison, Madison, WI, USA

[c] Madison Metropolitan Sewerage District, Madison, WI, USA

[*] Corresponding author. E-mail: *Hicks5@wisc.edu*

TiO_2 Containing Personal Care Product Inventory

(9 Tables, 87 Pages)

Table 1. Toothpaste

Brand	Product Name	Ingredients	Website URL
3M	ESPE Clinpro 5000	Clinpro 5000 toothpaste contains 1.1% sodium fluoride and an innovative tri-calcium phosphate ingredient which is sold exclusively through 3M ESPE. Each gram of Clinpro 5000 toothpaste contains 5mg of fluoride ion in a neutral pH base consisting of water, sorbitol, hydrated silica, glycerin, polyethylene-polypropylene glycol, flavor, polyethylene glycol, sodium lauryl sulfate, titanium dioxide, carboxymethyl cellulose, sodium saccharin and tri-calcium phosphate.	https://multimedia.3m.com/mws/media/544542O/clinprotm-5000-1-1-sodium-fluoride-anti-cavity-toothpaste.pdf
Aim	Mutli Benefit Tartar Control	Inactive Ingredients: Inactive IngredientsSorbitol, Water, Hydrated Silica, PEG-8, Sodium Lauryl Sulfate, SD Alcohol 38-B, Flavor, Cellulose Gum, Zinc Citrate Trihydrate, Sodium Saccharin, Blue 1, Mica, Titanium Dioxide.	https://www.walmart.com/ip/Aim-Multi-Benefit-Tartar-Control-Cool-Mint-Gel-5-5-Oz/55317030
Aim	Mega Bubble Berry	Active Ingredients Sodium Fluoride (0.24 percent). Inactive Ingredients Sorbital, Water, Hydrated, Silica, Peg-32, Sodium Lauryl Sulfate, Flavor, Cellulose Gum, Sodium Saccharin, Blue 1, Mica, Titanium Dioxide.	https://www.amazon.com/Bubble-Berry-Anticavity-Fluoride-Toothpaste/dp/B004ZUWMS4
Amway	Glister	Active Ingredients Sodium Fluoride (0.24 percent). Inactive Ingredients Sorbital, Water, Hydrated, Silica, Peg-32, Sodium Lauryl Sulfate, Flavor, Cellulose Gum, Sodium Saccharin, Blue 1, Mica, Titanium Dioxide.	http://www.amway.com/Shop/Product/Product.aspx/Glister-Multi-action-Fluoride-Toothpaste?itemno=E9530
Aquafresh	Cavity Protection	Active Ingredient Sodium Fluoride (0.25% w/v fluoride ion) Inactive Ingredients water, hydrated silica, sorbitol, glycerin, sodium lauryl sulfate, xanthan gum, flavor, titanium dioxide, PEG-8, sodium saccharin, carrageenan, polyethylene, red 30, blue 1 lake	https://www.aquafresh.com/products/everyday/cavity-protection-toothpaste/

Aquafresh	Everday	Active Ingredient Sodium fluoride 0.24% (0.15% w/v fluoride ion) Inactive Ingredients Water, hydrated silica, sorbitol, glycerin, pentasodium triphosphate, PEG-8, sodium lauryl sulfate, flavor, xantham gum, titanium, titanium dioxide, sodium hydroxide, sodium saccharin, sodium benzoate, blue 1 lake, red 30 lake	https://www.aquafresh.com/products/everyday/whitening-ultimate-white-toothpaste/
Aquafresh	Extreme Clean	Active Ingredient Sodium fluoride 0.25% (0.15% w/v fluoride ion) Inactive Ingredients Water, hydrated silica, sorbitol, glycerin, PEG-8, flavor, sodium lauryl sulfate, xanthan gum, titanium dioxide, cocamidopropyl betaine, sodium saccharin, iron oxide, red 30	https://www.aquafresh.com/products/extreme-clean/whitening-toothpaste/
Aquafresh	Maximum Strength Sensitive	Active Ingredients Potassium nitrate (5%) Sodium fluoride 0.24% (0.15% w/v fluoride ion) Inactive Ingredients Water, hydrated silica, sorbitol, glycerin, sodium lauryl sulfate, flavor, titanium dioxide, xantham gum sodium hydroxide, sodium saccharin, sodium benzoate, blue 1 lake, red 30 lake	https://www.aquafresh.com/products/everyday/toothpaste-for-sensitive-teeth/
Arm and Hammer	Advanced White Breath Freshening	Ingredients: Water, Sodium bicarbonate, Sorbitol, Hydrated silica, Glycerin, Tetrasodium pyrophosphate, Flavor, Sodium saccharin, Cellulose gum, Sodium lauroyl sarcosinate, Sodium lauryl sulfate, Titanium dioxide, Sodium fluoride	https://churchdwight.com/ingredient-disclosure/dental-care/40002536-arm-and-hammer-toothpaste-advance-white-breath-freshening.aspx
Colgate	Baking Soda and peroxied whitening-basic mint	Active Ingredient: Sodium Monofluorophosphate (0.76% (0.14% w/v Fluoride Ion)). Purpose: Anticavity. Inactive Ingredients: Glycerin, Hydrated Silica, Water, Sodium Bicarbonate, PEG-12, Sodium Lauryl Sulfate, Flavor, Sodium Hydroxide, Cellulose Gum, Carrageenan, Sodium Saccharin, Calcium Peroxide, Titanium Dioxide.	https://www.amazon.com/Colgate-Baking-Peroxide-Whitening-Toothpaste/dp/B00HXAIGUQ

Colgate	Max Clean	ACTIVE INGREDIENTS Sodium Fluoride (0.24% (0.14% w/v Fluoride Ion))Anticavity NON-ACTIVE INGREDIENTS Water, Sorbitol, Hydrated Silica, PEG-12, Poloxamer 407, Sodium Lauryl Sulfate, Flavor, Xanthan Gum, Cocamidopropyl Betaine, Tetrasodium Pyrophosphate, Sodium Saccharin, Methylcellulose, Mica, Titanium Dioxide, FD&C Blue No. 1	https://smartlabel.labelinsight.com/product/4404641/nonFoodIngredients
Colgate	Max Fresh with Whitening	Active Ingredient: Sodium Fluoride (0.24% (0.15% w/v Fluoride Ion)). Purpose: Anticavity. Inactive Ingredients: Sorbitol, Water, Hydrated Silica, PEG-12, Sodium Lauryl Sulfate, Flavor, Cellulose Gum, Tetrasodium Pyrophosphate, Cocamidopropyl Betaine, Sodium Saccharin, Methylcellulose, Titanium Dioxide, FD&C Blue No. 1, FD&C Yellow No. 5.	https://www.amazon.com/Colgate-Whitening-Toothpaste-Breath-Strips/dp/B00IAH18QU
Colgate	Prevident Sensitive	Active Ingredients Sodium fluoride 1.1% (w/w), Potassium nitrate 5% Inactive Ingredients water, hydrated silica, sorbitol, PEG-12, carrageenan, sodium lauryl sulfate, flavor, poloxamer 407, cocamidopropyl betaine, sodium saccharin, mica, sodium hydroxide, titanium dioxide, D&C yellow no. 10, FD&C blue no. 1	https://www.drugs.com/pro/prevident-5000-sensitive.html
Colgate	Prevident 5000	Active ingredient: Sodium fluoride 1.1% (w/w) Other Ingredients: Purified water, sorbitol, hydrated silica, PEG-12, tetrapotassium pyrophosphate, sodium lauryl sulfate, mint flavor (Spearmint flavor only), xanthan gum, sodium benzoate, fruit flavor (Fruitastic™* flavor only), sodium saccharin, titanium dioxide (Fruitastic™* flavor only), FD&C Blue #1 (Spearmint flavor only), D&C Red #33 (Fruitastic™* flavor only).	https://www.mountainside-medical.com/products/colgate-prevident-5000-plus-prescription-strength-toothpaste?variant=5650651588
Colgate	Sensitive Teeth Complete Protection	ACTIVE INGREDIENTS Potassium nitrate (5%)Antisensitivity, Sodium Fluoride (0.24% (0.14% w/v Fluoride Ion))Anticavity NON-ACTIVE INGREDIENTS Water, Hydrated Silica, Glycerin, Sorbitol, PEG-12, Tetrapotassium Pyrophosphate, PVM/MA Copolymer,	https://smartlabel.labelinsight.com/product/4404720/nonFoodIngredients

		Flavor, Sodium Lauryl Sulfate, Poloxamer 407, Sodium Hydroxide, Sodium Saccharin, Cellulose Gum, Xanthan Gum, Titanium Dioxide	
Colgate	Sensitive Whitening	ACTIVE INGREDIENTS Potassium nitrate (5%)Antisensitivity, Sodium Fluoride (0.24% (0.14% w/v Fluoride Ion))Anticavity NON-ACTIVE INGREDIENTS Water, Hydrated Silica, Glycerin, Sorbitol, PEG-12, PVM/MA Copolymer, Sodium Lauryl Sulfate, Flavor, Poloxamer 407, Trisodium Phosphate, Sodium Hydroxide, Cellulose Gum, Sodium Saccharin, Xanthan Gum, Titanium Dioxide, FD&C Blue No. 1	https://smartlabel.labelinsight.com/product/4404653/nonFoodIngredients
Colgate	Total Advanced Whitening	Active Ingredient: Sodium Fluoride (0.24% (0.16% w/v Fluoride Ion)). Purpose: Anticavity. Inactive Ingredients: Sorbitol, Water, Hydrated Silica, Glycerin, PEG-12, Pentasodium Triphosphate, Tetrasodium Pyrophosphate, Sodium Lauryl Sulfate, Flavor, Sodium Hydroxide, Sodium Saccharin, Cellulose Gum, Carrageenan, Titanium Dioxide.	https://www.amazon.com/Colgate-Total-Advanced-Whitening-Toothpaste/dp/B0033HK4DU
Colgate	Total clean mint	ACTIVE INGREDIENTS Sodium Fluoride (0.24% (0.14% w/v Fluoride Ion))Anticavity , Triclosan (0.30%)Antigingivitis NON-ACTIVE INGREDIENTS, Water, Hydrated Silica, Glycerin, Sorbitol, PVM/MA Copolymer, Sodium Lauryl Sulfate, Cellulose Gum, Flavor, Sodium Hydroxide, Carrageenan, Propylene Glycol, Sodium Saccharin, Titanium Dioxide	https://smartlabel.labelinsight.com/product/4051754/nonFoodIngredients
Colgate	Bubble Fruit	Active ingredients Sodium Fluoride (0.24%) (0.15% w/v Fluoride Ion). Inactive ingredients Sorbitol, Water, Hydrated Silica, Peg-12, Cellulose Gum, Sodium Lauryl Sulfate, Flavor, Sodium Saccharin, Mica, Titanium Dioxide, Fd&C Blue 1.	https://www.target.com/p/colgate-kids-cavity-protection-fluoride-toothpaste-bubble-fruit-4-6oz-2pk/-/A-15425277#

Colgate	Enamel Health	Active Ingredients: Potassium Nitrate (5%), Sodium Fluoride (0.24% (0.15% w/v Fluoride Ion)). Purpose: Antisensitivity, Anticavity. Inactive Ingredients: Water, Sorbitol, Hydrated Silica, Glycerin, PEG-12, Tetrasodium Pyrophosphate, Flavor, Sodium Lauryl Sulfate, Microcrystalline Cellulose, Zinc Phosphate, Cellulose Gum, Sodium Saccharin, Cocamidopropyl Betaine, Xanthan Gum, Mica, Titanium Dioxide, D&C Yellow No. 10, FD&C Blue No. 1	https://www.amazon.com/Colgate-Enamel-Health-Toothpaste-Multi-Protection/dp/B00TDNIWH8?th=1
Colgate	Kids	Ingredients: Active Ingredient - Purpose. Sodium Fluoride 0.24% (0.15% w/v Fluoride Ion) - Anticavity.Inactive Ingredients: Sorbitol, Water, Hydrated Silica, PEG-12, Cellulose Gum, Sodium Lauryl Sulfate, Flavor, Sodium Saccharin, Mica, Titanium Dioxide, FD&C Blue No. 1.	https://www.walmart.com/ip/Colgate-Kids-Cavity-Protection-Bubble-Fruit-Fluoride-Toothpaste-4-6-oz/35017670
Colgate	Maximum Cavity Protection	Active Ingredient - Purpose: Sodium Fluoride 0.24% (0.14% W/V Fluoride Ion) - Anticavity. Inactive Ingredients: Sorbitol, Water, Hydrated Silica, PEG-12, Cellulose Gum, Sodium Lauryl Sulfate, Flavor, Sodium Saccharin, Mica, Titanium Dioxide, D&C Yellow No. 10, FD&C Blue No. 1.	https://www.hy-vee.com/grocery/PD23591282/Colgate-Maximum-Cavity-Protection-Fluoride-Toothpaste-Mild-Bubble
Colgate	Tartar Control	Active Ingredient: Sodium Fluoride (0.24% (0.16% w/v Fluoride Ion)). Purpose: Anticavity. Inactive Ingredients: Sorbitol, Water, Hydrated Silica, Glycerin, PEG-12, Pentasodium Triphosphate, Tetrasodium Pyrophosphate, Sodium Lauryl Sulfate, Flavor, Sodium Hydroxide, Sodium Saccharin, Cellulose Gum, Carrageenan, Titanium Dioxide.	https://www.amazon.com/Colgate-Tartar-Protection-Toothpaste-Whitening/dp/B01BNF1USQ?th=1
Colgate	Total Deep Clean	Active Ingredients: Sodium Fluoride (0.24% (0.15% w/v Fluoride Ion)), Triclosan (0.30%). Purpose: Anticavity, Antigingivitis. Inactive Ingredients: Hydrated Silica, Water, Glycerin, Sorbitol, PVM/MA Copolymer, Sodium Lauryl	https://www.amazon.com/Colgate-Total-Advanced-Clean-Toothpaste/dp/

		Sulfate, Cellulose Gum, Flavor, Sodium Hydroxide, Carrageenan, Propylene Glycol, Sodium Saccharin, Titanium Dioxide.	B001H54U8K?th=1
Colgate	Total Daily Repair	Active Ingredients: Sodium Fluoride (0.24% (0.14% w/v Fluoride Ion)), Triclosan (0.30%). Purpose: Anticavity, Antigingivitis. Inactive Ingredients: Water, Hydrated Silica, Glycerin, Sorbitol, PVM/MA Copolymer, Sodium Lauryl Sulfate, Cellulose Gum, Flavor, Sodium Hydroxide, Propylene Glycol, Carrageenan, Sodium Saccharin, Titanium Dioxide.	https://www.amazon.com/Colgate-Total-Daily-Repair-Toothpaste/dp/B00ZANSGT4?th=1
Colgate	Total Pro Gum Health	Active Substances: Sodium Fluoride 0.32% w/w (1450ppmF¯), Triclosan 0.3%w/w, Glycerol, Silica Dental Type, Sorbitol Liquid (70%) Non-Crystalising, Purified Water, Poly (Methyl Vinyl Ether), Maleic Acid, Sodium Hydroxide (25% Solution), Sodium Laurilsulfate, Peppermint Flavour (contains Propylene Glycol), Carmellose Sodium, Titanium Dioxide (E171), Iota Carrageenan, Saccharin Sodium	https://www.ocado.com/webshop/product/Colgate-Total-Pro-Gum-Health-Whitening-Toothpaste/367517011
Colgate	Total Whitening	Active Ingredients: Sodium Fluoride (0.24% (0.14% w/v Fluoride Ion)), Triclosan (0.30%). Purpose: Anticavity, Antigingivitis. Inactive Ingredients: Water, Hydrated Silica, Glycerin, Sorbitol, PVM/MA Copolymer, Sodium Lauryl Sulfate, Cellulose Gum, Flavor, Sodium Hydroxide, Carrageenan, Propylene Glycol, Sodium Saccharin, Titanium Dioxide.	https://www.amazon.com/Colgate-Toothpaste-Total-Whitening-Triple/dp/B01LTHYW9W?th=1
Colgate	Ultrabrite	Sodium Fluoride (0.24%) (0.15% w/v Fluoride Ion), Sorbitol, Water, Hydrated Silica, PEG-12, Sodium Lauryl Sulfate, Flavor, Cellulose Gum, Tetrasodium Pyrophosphate, Cocamidopropyl Betaine, Sodium Saccharin, Titanium Dioxide.	https://www.amazon.com/Colgate-Advanced-Whitening-Fluoride-Toothpaste/dp/B01IKD5FW6
Colgate	Triple Action	Active ingredient - Purpose: Sodium fluoride 0.24% (0.15% W/V fluoride ion) - Anticavity. Inactive Ingredients: Sorbitol, Water, Hydrated Silica, Sodium Lauryl Sulfate, Flavor, Peg-12, Tetrasodium Pyrophosphate, Cocamidopropyl Betaine,	https://www.amazon.com/Colgate-Triple-Toothpaste-Original-6-4-

		Cellulose Gum, Sodium Saccharin, Xanthan Gum, Titanium Dioxide, FD&C Blue No.1, FD&C Yellow No. 5.	Ounce/dp/B003A4HSHG
Crest	3D White Arctic Fresh Icy Cool Mint	Sodium Fluoride, Water, Sorbitol, Hydrated Silica, Disodium Pyrophosphate, Sodium Lauryl Sulfate, Flavor, Cellulose Gum, Sodium Hydroxide, Sodium Saccharin, Carbomer, Xanthan Gum, Polyethylene, Mica, Titanium Dioxide, Blue 1 Lake	https://www.amazon.com/Crest-Arctic-Flavor-Whitening-Toothpaste/dp/B00HNNRG5E
Crest	3D White Radiant Mint	ACTIVE INGREDIENTS Sodium Fluoride 0.243% INACTIVE INGREDIENTS, Water, Sorbitol, Hydrated Silica, Disodium Pyrophosphate, Sodium Lauryl Sulfate, Flavor, Cellulose Gum, Sodium Hydroxide, Sodium Saccharin, Carbomer, Mica, Titanium Dioxide, Color	http://smartlabel.pg.com/00037000261513.html#ingredients
Crest	3D White Whitening Therapy Sensitivity Care	ACTIVE INGREDIENTS Sodium Fluoride 0.243%, Potassium Nitrate 5% INACTIVE INGREDIENTS Water, Hydrated Silica, Glycerin, Sorbitol, Trisodium Phosphate, Sodium Lauryl Sulfate, Flavor, Cellulose Gum, Alcohol (0.7%), Xanthan Gum, Sodium Saccharin, Polysorbate 80, Sodium Benzoate, Cetylpyridinium Chloride, Benzoic Acid, Titanium Dioxide, Color	http://smartlabel.pg.com/00037000733454.html#ingredients
Crest	Baking Soda and Peroxide	Active Ingredient: Sodium Fluoride 0.243% (0.15% W/V Fluoride Ion) Anticavity Toothpaste Inactive Ingredients: Sorbitol, Water, Hydrated Silica, Sodium Lauryl Sulfate, Trisodium Phosphate, Cellulose Gum, Flavor, Sodium Saccharin, Carbomer 956, Mica, Titanium Diozide, Blue 1	https://www.amazon.com/Crest-Peroxide-Whitening-Protection-Toothpaste/dp/B0046HHJ20
Crest	Cavity protection Regular	Active Ingredient: Sodium Fluoride 0.243% (0.15% W/V Fluoride Ion) Anticavity Toothpaste Inactive Ingredients: Sorbitol, Water, Hydrated Silica, Sodium Lauryl Sulfate, Trisodium Phosphate, Cellulose Gum, Flavor, Sodium Saccharin, Carbomer 956, Mica, Titanium Diozide, Blue 1	http://smartlabel.pg.com/00037000003403.html#ingredients

Crest	Complete Extra Whitening + Scope	ACTIVE INGREDIENTS Sodium Fluoride 0.243% INACTIVE INGREDIENTS Sorbitol, Water, Hydrated Silica, Sodium Lauryl Sulfate, Trisodium Phosphate, Flavor, Sodium Phosphate, Cellulose Gum, Carbomer, Sodium Saccharin, Titanium Dioxide, Color	https://www.costco.com/Crest-Complete-Extra-Whitening-%2B-Scope-Advanced-Toothpaste-8.2oz-(232g)%2C-5-pack-.product.100398747.html
Crest	Complete Multi-Benefit	Active Ingredients: Sodium Fluoride 0.243% (0.15% w/v Fluoride Ion). Inactive Ingredients: Sorbitol, Water, Hydrated Silica, Disodium Pyrophosphate, Flavor, Sodium Lauryl Sulfate, Sodium Hydroxide, Alcohol (0.7%), Xanthan Gum, Sodium Saccharin, Glycerin, Carbomer, Poloxamer 407, Polysorbate 80, Sodium Benzoate, Cetylpyridinium Chloride, Benzoic Acid, Mica, Titanium Dioxide, Blue 1, Yellow 5.	https://www.cvs.com/shop/crest-complete-multi-benefit-whitening-scope-toothpaste-prodid-1011341
Crest	Complete Whitening + Deep Clean	Active Ingredient: Sodium Fluoride 0.243% (0.15% W/V Fluoride Ion). Inactive Ingredients: Sorbitol, Water, Hydrated Silica, Peg-6, Sodium Lauryl Sulfate, Flavor, Zinc Citrate, Cellulose Gum, Carrageenan, Sodium Saccharin, Hydroxyethylcellulose, Sodium Citrate, Stannous Chloride, Mica, Titanium Dioxide, Blue 1.	https://www.walmart.com/ip/Crest-Complete-Whitening-Deep-Clean-Effervescent-Mint-Toothpaste-Choose-Count/17167870
Crest	Complete Whitening + Scope	Sorbitol, Water, Hydrated Silica, Disodium Pyrophosphate, Sodium Lauryl Sulfate, Flavor, Sodium Hydroxide, Alcohol (0.7%), Xanthan Gum, Sodium Saccharin, Glycerin, Carbomer, Cellulose Gum, Polysorbate 80, Sodium Benzoate, Cetylpyridinium Chloride, Benzoic Acid, Titanium Dioxide, Blue 1, Yellow 5	https://www.amazon.com/Crest-Complete-Whitening-Flavor-Toothpaste/dp/B005PLQIQ4?th=1
Crest	Gum Detoxify	Active Ingredients: Stannou8s Fluoride (0.454%) (0.14% w/v Fluoride Ion). Purposes: Anticavity, Antigingivitis, Antisensitivity Toothpaste. Inactive Ingredients: Water, Sorbitol, Hydrated	https://www.walmart.com/ip/Crest-Gum-Detoxify-Deep-Clean-

		Silica, Sodium Lauryl Sulfate, Carrageenan, Sodium Gluconate, Flavor, Xanthan Gum, Zinc Citrate, Stannous Chloride, Sodium Saccharin, Sodium Hydroxide, Sucralose, Titanium Dioxide.	Toothpaste-4-1-oz/510336166
Crest	Kids Sparkle Fun	Active ingredients Sodium Fluoride (0.243%) (0.15% w/v Fluoride Ion). Inactive ingredients Sorbitol, Water, Hydrated Silica, Sodium Lauryl Sulfate, Trisodium Phosphate, Sodium Phosphate, Cellulose Gum, Flavor, Sodium Saccharin, Carbomer, Mica, Titanium Dioxide, Blue 1.	https://www.target.com/p/crest-kids-cavity-protection-sparkle-fun-toothpaste-4-6-oz/-/A-13956414
Crest	Prohealth	ACTIVE INGREDIENTS Stannous Fluoride 0.454% INACTIVE INGREDIENTS Water, Sorbitol, Hydrated Silica, Sodium Lauryl Sulfate, Carrageenan, Sodium Gluconate, Flavor, Xanthan Gum, Zinc Citrate, Stannous Chloride, Sodium Hydroxide, Sodium Saccharin, Sucralose, Titanium Dioxide, Color & Aesthetics	http://smartlabel.pg.com/00037000995609.html#ingredients
Crest	Pro Health Advanced Extra Whitening Power	Active Ingredient: Stannous Fluoride 0.454% (0.16% W/V Fluoride Ion) Inactive Ingredients Glycerin, Hydrated Silica, Sodium Hexametaphosphate, Propylene Glycol, Peg-6, Water, Zinc Lactate, Trisodium Phosphate, Flavor, Sodium Lauryl Sulfate, Sodium Gluconate, Carrageenan, Sodium Saccharin, Xanthan Gum, Titanium Dioxide	https://www.amazon.com/Crest-Pro-Health-Whitening-Freshness-Toothpaste/dp/B019F0ENYE
Crest	Regular	ACTIVE INGREDIENTS Sodium Fluoride 0.243% INACTIVE INGREDIENTS Sorbitol, Water, Hydrated Silica, Sodium Lauryl Sulfate, Trisodium Phosphate, Flavor, Sodium Phosphate, Cellulose Gum, Carbomer, Sodium Saccharin, Titanium Dioxide, Color	http://smartlabel.pg.com/00037000003403.html#ingredients
Equate	Maximum Strenght Sensitive Extra whitening	Inactive Ingredients: Cocamidopropyl Betaine, Flavor, Glycerin, Hydrated Silica, PEG-8, Sodium Hydroxide, Sodium Methyl Cocoyl Taurate, Sodium Saccharin, Sodium Tripolyphosphate, Sorbitol, Titanium Dioxide, Water, Xanthan Gum	https://www.walmart.com/ip/Equate-Maximum-Strength-Sensitive-Extra-Whitening-Toothpaste-with-Fluoride-4-Oz/49943235

Equate	Anticavity Fluoride Radiant White	Inactive Ingredients: Water, Sorbitol, hydrated silica, disodium pyrophosphate, Sodium lauryl Sulfate, flavor, cellulose gum, sodium hydroxide, sodium saccharin, carbomer, mica titanium dioxide, blue 1.Active Ingredients: Sodium Fluoride	https://www.walmart.com/ip/Equate-Anticavity-Fluoride-Radiant-White-Tooth-Paste-Cool-Mint-4-8-Oz/912994761
Hello	Fluoride free kid's	Sorbitol (Humectant), Hydrated Silica (Polishes and Cleans Teeth), Purified Water, Vegetable Glycerin (Soothes and Moisturizes), Aloe Vera Gel (Soothes and Moisturizes), Xylitol (Sweetener), Erythritol (Sweetener), Natural Flavor (Yum), Xanthan Gum (Thickener), Lauryl Glucoside (Coconut/Palm and Corn Derived Cleanser), Calcium Glycerophosphate (Mineral), Titanium Dioxide (Mineral), Potassium Sorbate (maintains Stability) and Stevia Rebaudiana Leaf Extract (Sweetener).	https://www.amazon.com/Hello-Oral-Care-Toothpaste-Watermelon/dp/B01B4OB2FC?th=1
Hello	Activated Charcoal Whitening	sorbitol (humectant), hydrated silica (polishes and cleans teeth),vegetable glycerin (soothes and moisturizes), xylitol (sweetener), purified water, charcoal powder (whitens, polishes and cleans teeth/ freshens breath), flavor (freshens breath), xanthan gum (thickener), titanium dioxide (mineral), cocamidopropyl betaine (cleanser), sodium cocoyl glutamate (cleanser), coconut oil (soothes and moisturizes), potassium sorbate (maintains stability), stevia rebaudiana leaf extract* (sweetener).	https://www.hello-products.com/product/charcoal-whitening-toothpaste/
Hello	Extra Whitening Pure Mint	active ingredient: sodium fluoride 0.24% (0.15% w/v fluoride ion) inactive ingredients: sorbitol (humectant), hydrated silica (polishes and cleans teeth), glycerin (soothes and moisturizes), xylitol (sweetener), purified water, calcium carbonate (polishes and cleans teeth), flavor (freshens breath), xanthan gum (thickener), sodium lauryl sulfate (cleanser), titanium dioxide (appearance) and stevia rebaudiana leaf extract (sweetener).	https://www.hello-products.com/product/extra-whitening-fluoride-toothpaste/

Hello	Sensitive	Active: Potassium Nitrate 5% and Sodium Fluoride 0.24% Inactive: Sorbitol, Hydrated Silica, Purified Water, Xylitol, Aloe Vera Gel, Glycerin, Flavor, Coconut Oil, Xanthan Gum, Titanium Dioxide, Cocamidopropyl Betaine, Sodium Cocoyl Glutamate and Stevia Rebaudiana Leaf Extract.	https://www.amazon.com/Hello-Oral-Care-Sensitivity-Toothpaste/dp/B01N4IEXJK?th=1
Kirkland	Signature Pro Complete Plus Whitening	Active ingredient Sodium fluoride (0.15% w/v fluoride ion) Inactive ingredients water, hydrated silica, sorbitol, glycerin, pentasodium triphosphate, PEG-8; sodium lauryl sulfate, titanium dioxide, flavor, xanthan gum, sodium hydroxide, sodium saccharin, blue 1	https://www.drugs.com/otc/110026/kirkland-signature-pro-complete-plus-whitening.html
Luster Now!	Instant Whitening	Inactive Ingredients: Inactive IngredientsSorbitol, Silica, Water, Propylene Glycol, Sodium Lauryl Sulfate, Flavor, Disodium Phosphate, PVM/MA Copolymer, Cellulose Gum, Sodium Benzoate, Potassium Acesulfame, Xanthan Gum, Tetrasodium Pyrophosphate, Titanium Dioxide, FD&C Blue No. 1 (CI 42090). Ingredients: Water, Silica, Sorbitol, Glycerin, Pvm/Ma Copolymer, Propylene Glycol, Sodium Lauroyl Sarcosinate, Flavor, Acrylates/C10-C30 Alkyl Acrylate Crosspolymer, Acesulfame Potassium, Sodium Benzoate, Sodium Hydroxide, Tetrasodium Pyrophosphate, Ci 77891, Ci 42090, Ci 77019. Active Ingredients: Sodium Fluoride 0.24%	https://www.walmart.com/ip/Luster-Now-Instant-Whitening-Daily-Fluoride-Enamel-Safe-Effective-Professional-Teeth-Whitening-Toothpaste-Mint-4-oz/21695270
Marvis	Classic Strong Mint	Classic Strong Mint - Glycerin, Aluminum Hydroxide, Aqua (Water/Eau), Silica, Aroma (Flavor), Cellulose Gum, Titanium Dioxide, Sodium Lauryl Sulfate, Sodium Saccharin, Sodium Citrate, Citric Acid, Eugenol, Limonene, Benzyl Alcohol.	https://www.birchbox.com/product/5203
Pepsodent	Complete Care	Active Ingredients: Sodium Fluoride (0.24%) Inactive Ingredients: Sorbitol, Water, Hydrated Silica, PEG-8, Sodium Lauryl Sulfate, SD Alcohol 38-B, Flavor, Cellulose Gum, Sodium Saccharin, Titanium Dioxide	https://www.walgreens.com/store/c/pepsodent-complete-care-toothpaste/ID=prod6323978-product

Plus White	Whitening Protection	Active Ingredient: Sodium Monofluorophosphate (0.15% W/V Fluoride Ion). Inactive Ingredients: Hydrated Silica, Sorbitol, Water (Aqua), Dicalcium Phosphate, Glycerin, Sodium Lauryl Sulfate, Flavor, Cellulose Gum, Titanium Dioxide, Trisodium Phosphate, Pvm/Ma Copolymer, Sodium Saccharin, Blue 1.	https://www.amazon.com/Plus-White-Whitening-Protection-Toothpaste/dp/B000NSQK3U?th=1
Public Goods		Vegetable Glycerin (from vegetable oils), Sorbitol (from corn), Water, Silica (from sand), Xylitol (from corn), Cocoamidopropyl Betaine (from coconut), Titanium Dioxide (a natural mineral), Mentha Piperita (peppermint) Oil, Carboxymethyl Cellulose (a food grade thickener).	https://www.publicgoods.com/products/toothpaste
Sensodyne	Complete Protection Extra Fresh	Active ingredients STANNOUS FLOURIDE (0.15% W/V FLUORIDE ION) (0.454%) Inactive ingredients Glycerin, Peg-8, Hydrated Silica, Pentasodium Triphosphate, Sodium Lauryl Sulfate, Flavor, Titanium Dioxide, Polyacrylic Acid, Cocamidopropyl Betaine, Sodium Saccharin	https://www.target.com/p/sensodyne-complete-extra-fresh-toothpaste-3-4oz/-/A-16200657
Sensodyne	Extra Whitening	Inactive Ingredients: Water; Hydrated Silica; Sorbitol; Glycerin; Pentasodium; Triphosphate; PEG-8; Flavor; Titanium Dioxide; Sodium Methyl Cocoyl Taurate; Cocamidopropyl Betaine; Xanthan Gum; Sodium Hydroxide; Sodium Saccharin; Sucralose Ingredients: Active: Potassium Nitrate (5%)*--Antihypersensitivity, Sodium Fluoride (0.15% w/V Fluoride Ion)--Anticavity. Inactive: Cocoamidopropyl Betaine, Flavor, Glycerin, Hydrated Silica, PEG-8, Pentasodium Triphosphate, Sodium Hydroxide, Sodium Methyl Cocoyl Taurate, Sodium Saccharin, Sorbitol, Sucralose, Titanium Dioxide, Water, Xanthan Gum.	https://www.walmart.com/ip/Sensodyne-Sensitivity-Toothpaste-Extra-Whitening-for-Sensitive-Teeth-4-ounce/3392

Sensodyne	Pronamel Daily Fluoride	Active Ingredients: Potassium nitrate - 5 % (Antihypersensitivity), Sodium fluoride - 0.25 % 0.15% w/v fluoride ion (Anticavity) Inactive Ingredients: water, sorbitol, hydrated silica, glycerin, PEG-8, cocamidopropyl betaine, flavor, xanthan gum, sodium saccharin, titanium dioxide, sodium hydroxide	https://www.walgreens.com/store/c/sensodyne-pronamel-daily-fluoride-toothpaste-for-sensitive-teeth-mint-essence/ID=prod3194270-product
Sensodyne	Pronamel Whitening	Active Ingredients - Purpose.Potassium Nitrate 5% - Antihypersensitivity.Sodium Fluoride 0.15% w/v Fluoride Ion - Anticavity.Inactive Ingredients: Water, Sorbitol, Hydrated Silica, Glycerin, PEG-8, Cocamidopropyl Betaine, Flavor, Titanium Dioxide, Xanthan Gum, Sodium Saccharin, Sodium Hydroxide.	https://www.walmart.com/ip/Sensodyne-Pronamel-Gentle-Whitening-Fluoride-Toothpaste-to-Strengthen-and-Protect-Enamel-4-Ounce-Twinpack-2-tubes-of-4oz/17248395
Sensodyne	Repair and Protect	glycerin, PEG-8, hydrated silica, pentasodium triphosphate, sodium lauryl sulfate, flavor, titanium dioxide, polyacrylic acid, cocamidopropyl betaine, sodium saccharin	https://www.amazon.com/Sensodyne-Protect-Sensitivity-Toothpaste-Sensitive/dp/B00AWKQWI2
SF 5000 Plus		ACTIVE INGREDIENTS: Sodium Fluoride USP 1.1% (w/w) INACTIVE INGREDIENTS: Purified Water, Sorbitol, Hydrated Silica, Glycerin, Tetrapotassium Pyrophosphate, Flavor, PEG 12, Sodium Lauryl Sulfate, Cellulose Gum, Sodium Saccharin, Titanium Dioxide, FD&C Blue No. 1.	https://www.drugs.com/pro/sf-5000-plus.html
Smart Sense	Sensitive Enamel Guard	Active Ingredients: Potassium Nitrate (5%)(Purpose: Antihypersensitivity), Sodium Fluoride (0.15% W/V Fluoride Ion)(Purpose: Anticavity). Inactive Ingredients: Cocamidopropyl Betaine, Flavor, Glycerin, Hydrated Silica, PEG-8,	https://www.kmart.com/smart-sense-toothpaste-fluoride-daily-anti-cavity-

		Sodium Hydroxide, Sodium Saccharin, Sorbitol, Titanium Dioxide, Water, Xanthan Gum.	sensitive-enamel-guard-fresh/p-038W020545050001P
Spry	Antiplaque Tartar Control	Ingredients: Water (aqua), glycerin, silica, xylitol, erythritol, lauryl glucoside, sodium methyl cocoyl taurate, zinc citrate, vaccinium macrocarpon (cranberry) fruit extract, aloe barbadensis leaf juice, xanthan gum, cellulose gum, stevia rebaudiana leaf/stem powder, flavor (natural peppermint), sodium benzoate, titanium dioxide.	https://www.vitacost.com/xlear-spry-anti-plaque-tartar-control-toothpaste-fluoride-free
Systema	Gum Care Icy Cool Mint	Sorbitol, Water, Silica, PEG-8, Sodium Lauryl Sulfate, Cellulose Gum, Flavor, Titanium Dioxide, Sodium Saccharin, Sodium Fluoride, Methylparaben, Dipotassium Glycyrrhizate, O-Cymen-5-Ol, Butylparaben	https://www.fairprice.com.sg/product/systema-gum-care-toothpaste-icy-cool-mint-160g-13026014
Tom's of Maine	Rapid Relief Sensitive	Arginine bicarbonate, benzyl alcohol, calcium carbonate, hydrated silica, natural flavor, sodium bicarbonate, sodium lauryl sulfate, sorbitol, titanium dioxide, water, xanthan gum, xylitol	https://www.amazon.com/Toms-Maine-Sensitive-Natural-Toothpaste/dp/B016K8KF64?th=1
TopCare	Sensitive Maximum Strength W/Fluoride Extra Whitening	Active Ingredients: Potassium Nitrate (5%), Sodium Fluoride (0.24%) (0.15% w/v Fluoride Ion). Purpose: Antihypersensitivity; Anticavity. Inactive Ingredients: Cocamidopropyl Betaine, Flavor, Glycerin, Hydrated Silica, PEG-8, Sodium Hydroxide, Sodium Methyl Cocoyl Taurate, Sodium Saccharin, Sodium Tripolyphosphate, Sorbitol, Titanium Dioxide, Water, Xanthan Gum.	https://www.justsavefoods.com/shop?product_id=591013
Ultrabrite	Advcanced Whitening	Sodium Fluoride (0.24%) (0.15% w/v Fluoride Ion), Sorbitol, Water, Hydrated Silica, PEG-12, Sodium Lauryl Sulfate, Flavor, Cellulose Gum, Tetrasodium Pyrophosphate, Cocamidopropyl Betaine, Sodium Saccharin, Titanium Dioxide.	https://www.amazon.com/Ultra-brite-Advanced-Whitening-Toothpaste/dp/B0000530SD

Table 2. Shampoo

Brand	Product Name	Ingredients	URL
Aveeno	Nourish + Moisturize	Water, Ammonium Lauryl Sulfate, Dimethicone, Sodium Cumenesulfonate, Cocamide MEA, Cetyl Alcohol, Triticum Vulgare (Wheat) Gluten, Triticum Vulgare (Wheat) Germ Oil, Avena Sativa (Oat) Peptide, Mauritia Flexuosa Fruit Oil, Acrylates Copolymer, Cocamidopropyl Betaine, Caprylyl Glycol, Fragrance, Phenoxyethanol, Glycol Distearate, Tetrasodium EDTA, Guar Hydroxypropyltrimonium Chloride, Polyquaternium10, Glycerin, Mica, Titanium Dioxide. This product contains Wheat Protein. May also contain: Citric Acid, Sodium Hydroxide.	https://www.amazon.com/Aveeno-Nourish-Moisturize-Shampoo-Ounce/dp/B001QT3GOQ?th=1
Aveeno	Nourish + Shine	Water, Ammonium Lauryl Sulfate, Dimethicone, Sodium Cumenesulfionate, Cocamide MEA, Cetyl Alcohol, Acrylates Copolymer, Cocamidopropyl Betaine, Fragrance, Phenoxyethanol, Caprylyl Glycol, Glycol Distearate, Tetrasodium EDTA, Guar Hydroxypropyltrimonium Chloride Triticum Vulgare (Wheat) Germ Oil, Triticum Vulgare (Wheat) Gluten, Orbignya Speciosa Kernel Oil, Glycerin, Polyquaternium10, Astrocaryum Murumuru Seed Butter, Mauritia Flexuosa Fruit Oil, Mica, Titanium Dioxide. May Also Contai: Citric Acid, Sodium Hydroxide.	https://www.amazon.com/Aveeno-Nourish-Shine-Shampoo-Ounce/dp/B004ZJX572?th=1
Aveeno	Nourish + Strenghten	Water, ammonium lauryl sulfate, dimethicone, sodium cumenesulfonate, cocamide MEA, cetyl alcohol, triticum vulgare (wheat) gluten, triticum vulgare (wheat) germ oil, mauritia flexuosa fruit oil, acrylates copolymer, cocamidopropyl betaine, caprylyl glycol, fragrance, phenoxyethanol, glycol distearate, tetrasodium EDTA, guar hydroxypropyltrimonium chloride, glycerin, polyquaternium-10, mica, titanium dioxide.May also contain: citric	https://www.amazon.com/Aveeno-Nourish-Strengthen-Shampoo-Damaged/dp/B0067H6EGE

		acid, sodium hydroxide.This product contains wheat protein.	
Aveeno	Nourish + Volumize	Water, ammonium lauryl sulfate, dimethicone, sodium cumenesulfonate, cocamide MEA, cetyl alcohol, acrylates copolymer, cocamidopropyl betaine, fragrance, phenoxyethanol, caprylyl glycol, glycol distearate, tetrasodium EDTA, citric acid, guar hydroxypropyltrimonium chloride, glycerin, sodium hydroxide, triticum vulgare (wheat) germ oil, polyquaternium-10, mauritia flexuosa fruit oil, triticum vulgare (wheat) gluten, nymphaea coerulea flower extract, mica, titanium dioxide.This product contains wheat protein.	https://www.amazon.com/Aveeno-Nourish-Volumize-Lightweight-Shampoo/dp/B001QT8CVI?th=1
Aveeno	Pure Renewal	Water, Sodium Cocoyl Isethionate, Cocamidopropyl Betaine, Ammonium Lauroyl Sarcosinate, Acrylates Copolymer, Cocamide MEA, Dimethicone, Sodium Hydrolyzed Potato Starch Dodecenylsuccinate, Fragrance, Phenoxyethanol, Sodium Hydroxide, Citric Acid, Caprylyl Glycol, Propylene Glycol, Glycerin, Guar Hydroxypropyltrimonium Chloride, Laminaria Saccharina Extract, Polyquaternium7, Methylisothiazolinone, Titanium Dioxide, Mica.	https://www.amazon.com/Aveeno-Renewal-Shampoo-Moisturizing-Sulfate-Free/dp/B0067H6E9G
Axe	Gold Wash and Style	Water (Aqua), Sodium Laureth Sulfate, Cocamidopropyl Betaine, Sodium Chloride, Dimethiconol, Fragrance (Parfum), Carbomer, Citric Acid, Propylene Glycol, Guar Hydroxypropyltrimonium Chloride, TEA-Dodecylbenzenesulfonate, Tetrasodium EDTA, DMDM Hydantoin, PPG-9, Methylchloroisothiazolinone, Methylisothiazolinone, Mica (CI 77019), Titanium Dioxide (CI 77891), Blue 1 (CI 42090), Yellow 5 (CI 19140).	https://www.ulta.com/gold-shampoo-wash-style?productId=xlsImpprod17771687

Axe	Apollo 2 in 1	Water (Aqua), Sodium Laureth Sulfate, Cocamidopropyl Betaine, Sodium Chloride, Dimethiconol, Fragrance (Parfum), Carbomer, Citric Acid, Propylene Glycol, Guar Hydroxypropyltrimonium Chloride, TEA-Dodecylbenzenesulfonate, Tetrasodium EDTA, DMDM Hydantoin, PPG-9, Methylchloroisothiazolinone, Methylisothiazolinone, Mica (CI 77019), Titanium Dioxide (CI 77891), Blue 1 (CI 42090), Yellow 5 (CI 19140)	https://www.amazon.com/Axe-Shampoo-Conditioner-Apollo-12/dp/B00AE07B9O?th=1
Clear	24/7 Total Care	Water (Aqua), Sodium Laureth Sulfate, Cocamidopropyl Betaine, Sodium Chloride, Dimethiconol, Carbomer, Fragrance (Parfum), Dimethicone, Zinc Pyrithione, DMDM Hydantoin, Guar Hydroxypropyltrimonium Chloride, Citric Acid, Glycerin, Zinc Sulfate, TEA-Dodecylbenzenesulfonate, Poloxamer 407, Laureth-23, Laureth-4, Elaeis Guineensis (Palm) Oil, Helianthus Annuus (Sunflower) Seed Oil, PPG-9, Aloe Barbadensis Leaf Juice, Butylene Glycol, Propylene Glycol, Iodopropynyl Butylcarbamate, PEG-4, Xanthan Gum, Glycine Soja (Soybean) Oil, Methylchloroisothiazolinone, Methylisothiazolinone, Ascorbic Acid, Panthenol, Tocopheryl Acetate, Cocos Nucifera (Coconut) Fruit Extract, Biotin, Niacinamide, Mica (CI 77019), Titanium Dioxide (CI 77891), Red 33 (CI 17200)	https://www.amazon.com/Clear-Total-Omega-3-Coconut-Shampoo/dp/B007RTR898

Clear	Color and Heat Conqueror	Water (Aqua), Sodium Laureth Sulfate, Cocamidopropyl Betaine, Sodium Chloride, Dimethiconol, Carbomer, Fragrance (Parfum), Dimethicone, Zinc Pyrithione, DMDM Hydantoin, Guar Hydroxypropyltrimonium Chloride, Citric Acid, Glycerin, Zinc Sulfate, TEA-Dodecylbenzenesulfonate, Poloxamer 407, Laureth-23, Butylene Glycol, Laureth-4, Elaeis Guineensis (Palm) Oil, Helianthus Annuus (Sunflower) Seed Oil, PPG-9, Aloe Barbadensis Leaf Juice, Propylene Glycol, Iodopropynyl Butylcarbamate, PEG-4, Xanthan Gum, Glycine Soja (Soybean) Oil, Rubus Occidentalis Fruit Juice, Methylchloroisothiazolinone, Rubus Fruticosus (Blackberry) Fruit Extract, Methylisothiazolinone, Ascorbic Acid, Panthenol, Tocopheryl Acetate, Biotin, Niacinamide, Mica (CI 77019), Titanium Dioxide (CI 77891), Red 33 (CI 17200)	https://www.amazon.com/Clear-Shampoo-Color-Heat-Conqueror/dp/B007RTR89S?th=1
Clear	Extreme Damage Relief	Water (Aqua), Sodium Laureth Sulfate, Cocamidopropyl Betaine, Sodium Chloride, Dimethiconol, Carbomer, Fragrance (Parfum), Dimethicone, Zinc Pyrithione, DMDM Hydantoin, Guar Hydroxypropyltrimonium Chloride, Citric Acid, Glycerin, Zinc Sulfate, TEA-Dodecylbenzenesulfonate, Poloxamer 407, Laureth-23, Laureth-4, Elaeis Guineensis (Palm) Oil, Helianthus Annuus (Sunflower) Seed Oil, PPG-9, Aloe Barbadensis Leaf Juice, Butylene Glycol, Propylene Glycol, Iodopropynyl Butylcarbamate, PEG-4, Xanthan Gum, Hydrolyzed Keratin, Glycine Soja (Soybean) Oil, Methylchloroisothiazolinone, Methylisothiazolinone, Ceramide NG, Ascorbic Acid, Panthenol, Tocopheryl Acetate, Biotin, Niacinamide, Mica (CI 77019), Titanium Dioxide (CI 77891), Red 33 (CI 17200)	https://www.amazon.com/Clear-Shampoo-Extreme-Damage-Relief/dp/B00Q70QKBU

Clear	Hydration Fix	Water (Aqua), Sodium Laureth Sulfate, Cocamidopropyl Betaine, Dimethiconol, Sodium Chloride, Fragrance (Parfum), Carbomer, Dimethicone, Zinc Pyrithione, DMDM Hydantoin, Guar Hydroxypropyltrimonium Chloride, Citric Acid, Glycerin, Zinc Sulfate, TEA-Dodecylbenzenesulfonate, Poloxamer 407, Laureth-23, Butylene Glycol, Laureth-4, Elaeis Guineensis (Palm) Oil, Helianthus Annuus (Sunflower) Seed Oil, PPG -9, Aloe Barbadensis Leaf Juice, Iodopropynyl Butylcarbamate, PEG-4, Xanthan Gum, Glycine Soja (Soybean) Oil, Methylchloroisothiazolinone, Methylisothiazolinone, Ascorbic Acid, Panthenol, Tocopheryl Acetate, Spirulina Maxima Extract, Mentha Viridis (Spearmint) Leaf Extract, Nymphaea Alba Flower Extract, Biotin, Niacinamide, Mica (CI 77019), Titanium Dioxide (CI 77891).	https://www.walmart.com/ip/Clear-Hydration-Fix-Hydrating-Shampoo-12-9-fl-oz/35056188
Dove	Daily Moisture	Water (Aqua), Sodium Laureth Sulfate, Glycol Distearate, Cocamidopropyl Betaine, Sodium Chloride, Fragrance (Parfum), Glycerin, Dimethicone, Dimethiconol, Acrylates/Beheneth-25 Methacrylate Copolymer, Styrene/Acrylates Copolymer, Guar Hydroxypropyltrimonium Chloride, Citric Acid, Tetrasodium Edta, Amodimethicone, Dmdm Hydantoin, Peg-45M, Tea-Dodecylbenzenesulfonate, Cocamide Mea, Lysine Hcl, Arginine, Peg-9M, Cetrimonium Chloride, Ppg-9, Propylene Glycol, Methylchloroisothiazolinone, Methylisothiazolinone, Mica (Ci 77019), Titanium Dioxide (Ci 77891), Yellow 5 (Ci 19140), Red 33 (Ci 17200).	https://www.dove.com/us/en/hair-care/shampoo/daily-moisture-shampoo.html

Dove	Nourishing Rituals	Water (Aqua), Sodium Laureth Sulfate, Cocamidopropyl Betaine, Glycerin, Sodium Chloride, Dimethiconol, Fragrance (Parfum), Citric Acid, Sodium Benzoate, Glycol Distearate, Carbomer, Gluconolactone, PPG-9, Guar Hydroxypropyltrimonium Chloride, TEA-Dodecylbenzenesulfonate, Cocamide MEA, Sodium Sulfate, Trehalose, Disodium EDTA, PEG-45M, Curcuma Aromatica Root Oil, Cocos Nucifera (Coconut) Oil, Mica (CI 77019), Titanium Dioxide (CI 77891)	https://www.dove.com/us/en/hair-care/shampoo/repairing-ritual-shampoo.html
Dove	Nutritive Intensive Repair	Water (Aqua), Sodium Laureth Sulfate, Glycol Distearate, Cocamidopropyl Betaine, Sodium Chloride, Fragrance (Parfum), Glycerin, Dimethicone, Gluconolactone, Ppg-9, Dimethiconol, Acrylates/Beheneth-25 Methacrylate Copolymer, Styrene/Acrylates Copolymer, Citric Acid, Guar Hydroxypropyltrimonium Chloride, Sodium Sulfate, Adipic Acid, Trehalose, Tetrasodium Edta, Amodimethicone, Dmdm Hydantoin, Peg-45M, Tea-Dodecylbenzenesulfonate, Cocamide Mea, Peg-9M, Cetrimonium Chloride, Methylchloroisothiazolinone, Methylisothiazolinone, Mica (Ci 77019), Titanium Dioxide (Ci 77891).	https://www.dove.com/us/en/hair-care/shampoo/intensive-repair-shampoo.html
Equate	Smoothing Keratin	Ingredients: Water (aqua), sodium laureth sulfate, cocamidopropyl betaine, potassium chloride, glycerin, dimethiconol, fragrance (parfum), carbomer, styrene/acrylates copolymer, guar hydroxypropyltrimonium chloride, TEA-dodecylbenzenesulfonate, glycol distearate, citric acid, tetrasodium edta, dmdm hydantoin, hydrolyzed keratin, peg-45m, methylchlorosiothiazolinone, methylisothiazolinone, PPG-9, mica, titanium dioxide	https://www.walmart.com/ip/Equate-Beauty-Smoothing-Keratin-Shampoo-25-Fl-Oz/48693083

Pantene	Moisture Renewal	Water, Sodium Laureth Sulfate, Sodium Citrate, Cocamidopropyl Betaine, Sodium Xylenesulfonate, Stearyl Alcohol, Sodium Lauryl Sulfate, Sodium Chloride, Cetyl Alcohol, Fragrance, Citric Acid, Sodium Benzoate, Polyquaternium-6, Guar Hydroxypropyltrimonium Chloride, Tetrasodium Edta, Trisodium Ethylenediamine Disuccinate, Trihydroxystearin, Mica, Titanium Dioxide, Panthenol, Panthenyl Ethyl Ether, Silica, Iron Oxides, Methylchloroisothiazolinone, Methylisothiazolinone	https://pantene.com/en-us/product/daily-moisture-renewal-shampoo
Selsun Blue	Medicated Shampoo	Active ingredient Selenium sulfide 1% Inactive ingredients water, ammonium lauryl sulfate, distearyl phthalic acid amide, ammonium laureth sulfate, sodium chloride, cocamide DEA, dimethicone, aloe barbadensis leaf juice, hydroxypropyl methylcellulose, sodium isostearoyl lactylate, DMDM hydantoin, fragrance, citric acid, sodium citrate, titanium dioxide, blue 1 (283-134)	https://www.clockmedical.com/customer/docs/skudocs/2650_Ingredients.pdf
Suave	Almond and Shea Butter	Ingredients: Water (Aqua), Sodium Laureth Sulfate, Cocamidopropyl Betaine, Sodium Chloride, Glycerin, Prunus Amygdalus Dulcis (Sweet Almond) Oil, Butyrospermum Parkii (Shea Butter), Aloe Barbadensis Leaf Juice (Aloe Vera), Cymbopogon Schoenanthus Oil (Lemongrass), Rosmarinus Officinalis (Rosemary) Leaf Oil, Chamomilla Recutita (Matricaria) Flower/Leaf Extract, Lavandula Angustifolia (Lavender) Flower Extract, Simmondsia Chinensis (Jojoba) Seed Oil, Dimethiconol, Fragrance (Parfum), Carbomer, Guar Hydroxypropyltrimonium Chloride, TEA-Dodecylbenzenesulfonate, PPG-9, Tetrasodium EDTA, TEA-Sulfate, DMDM Hydantoin, Citric Acid, PEG-45M, Methylchloroisothiazolinone, Methylisothiazolinone, Mica (CI 77019) Titanium Dioxide (CI 77891).	https://www.walmart.com/ip/Suave-Professionals-Almond-Shea-Butter-Moisturizing-Shampoo-28-oz/13269721

Tigi	Dumb Blonde Toning	Water (Aqua/Eau), Sodium Cocoyl Isethionate, Sodium Lauryl Sulfoacetate, Cocamidopropyl Betaine, Glycol Distearate, Glycerin, Sodium Chloride, Phenoxyethanol, Fragrance (Parfum), Carbomer, Sodium Isethionate, Dimethiconol, Polyquaternium-10, PPG-9, Laureth-4, Sodium Hydroxide, Mica, Disodium EDTA, TEA-Dodecylbenzenesulfonate, Amodimethicone, Triethanolamine, Sodium Benzoate, Lactic Acid, Citric Acid, PEG-45M, Magnesium Nitrate, Isopropyl Alcohol, Sodium Acetate, Trideceth-12, Cetrimonium Chloride, Methylchloroisothiazolinone, Silica, Magnesium Chloride, Methylisothiazolinone, Nonfat Dry Milk (Sine Adipe Lac/Poudre de Lait Écrémé), PEG-40 Hydrogenated Castor Oil, Trideceth-9, Hydrolyzed Pearl, Oryza Sativa (Rice) Bran Extract, Oryza Sativa (Rice) Germ Oil, Xanthan Gum, 1,2-Hexanediol, Caprylyl Glycol, Potassium Sorbate, Butylphenyl Methylpropional, Hexyl Cinnamal, Linalool, Ext. Violet 2 (CI 60730), Titanium Dioxide (CI 77891).	https://www.ulta.com/dumb-blonde-violet-toning-shampoo?productId=xlsImpprod6481106
Tigi	URBAN ANTIDOTES LEVEL 1 RE-ENERGIZE	Water (Aqua/Eau), Sodium Laureth Sulfate, Cocamidopropyl Betaine, Glycol Distearate, Fragrance (Parfum), Sodium Chloride, Glycerin, Carbomer, Citric Acid, Polyquaternium-10, Tetrasodium EDTA, DMDM Hydantoin, Sodium Benzoate, PEG-45M, Magnesium Nitrate, Isopropyl Alcohol, Sodium Acetate, PPG-9, Magnesium Chloride, Silica, Methylchloroisothiazolinone, Methylisothiazolinone, Butylphenyl Methylpropional, Hexyl Cinnamal, Linalool, Mica (CI 77019), Titanium Dioxide (CI 77891).	https://www.tigi.com/bedhead/us/product/urban-antidotes-level-1-re-energize-shampoo/

Tigi	Colour Goddess	Water (Aqua/Eau), Sodium Laureth Sulfate, Cocamidopropyl Betaine, Glycerin, Potassium Chloride, Fragrance (Parfum), Glycol Distearate, Dimethiconol, Cocos Nucifera (Coconut) Oil, Linum Usitatissimum (Linseed) Seed Oil, Glycine Soja (Soybean) Oil, Phenoxyethanol, Carbomer, Sodium Chloride, Amodimethicone, Hydrolyzed Keratin, Prunus Amygdalus Dulcis (Sweet Almond) Oil, Butyrospermum Parkii (Shea) Butter, Guar Hydroxypropyltrimonium Chloride, TEA-Dodecylbenzenesulfonate, Laureth-23, Citric Acid, Trideceth-12, Disodium EDTA, Sodium Benzoate, PEG-45M, Magnesium Nitrate, Benzophenone-4, Magnesium Carbonate, Cetrimonium Chloride, Panthenol, PPG-9, Tocopheryl Acetate, Silica, Methylchloroisothiazolinone, Magnesium Chloride, Methylisothiazolinone, Mica (CI 77019), Titanium Dioxide (CI 77891), Iron Oxides (CI 77491), Blue 1 (CI 42090), Red 33 (CI 17200), Yellow 5 (CI 19140).	https://www.tigi.com/bedhead/us/product/colour-goddess-oil-infused-shampoo-for-coloured-hair/
Tigi	Urban Antidotes level 2 recovery	Water (Aqua/Eau), Sodium Laureth Sulfate, Cocamidopropyl Betaine, Glycol Distearate, Fragrance (Parfum), Sodium Chloride, Guar Hydroxypropyltrimonium Chloride, Dimethiconol, Glycerin, Carbomer, Citric Acid, TEA-Dodecylbenzenesulfonate, PPG-9, Laureth-23, Tetrasodium EDTA, DMDM Hydantoin, Sodium Benzoate, PEG-45M, Phenoxyethanol, Magnesium Nitrate, Magnesium Chloride, Silica, Methylchloroisothiazolinone, Methylisothiazolinone, Benzyl Benzoate, Geraniol, Limonene, Linalool, Mica (CI 77019), Titanium Dioxide (CI 77891), Blue 1 (CI 42090), Yellow 5 (CI 19140).	https://www.tigi.com/bedhead/us/product/urban-antidotes-level-2-recovery-shampoo/

Tigi	Urban Antidotes Level 3 Resurrection	Water (Aqua/Eau), Sodium Laureth Sulfate, Cocamidopropyl Betaine, Fragrance (Parfum), Dimethiconol, Glycol Distearate, Sodium Chloride, Glycerin, Carbomer, TEA-Dodecylbenzenesulfonate, Guar Hydroxypropyltrimonium Chloride, Amodimethicone, Laureth-23, Citric Acid, PPG-9, Tetrasodium EDTA, DMDM Hydantoin, Phenoxyethanol, Sodium Benzoate, PEG-45M, Magnesium Nitrate, Cetrimonium Chloride, Trideceth-12, Magnesium Chloride, Silica, Methylchloroisothiazolinone, Methylisothiazolinone, Limonene, Linalool, Mica (CI 77019), Titanium Dioxide (CI 77891).	https://www.tigi.com/bedhead/us/product/urban-antidotes-level-3-resurrection-shampoo/
Tigi	Elasticate	Water (Aqua/Eau), Sodium Laureth Sulfate, Cocamidopropyl Betaine, Dimethiconol, Glycol Distearate, Sodium Chloride, Fragrance (Parfum), Glycerin, Phenoxyethanol, Carbomer, Hydrolyzed Wheat Protein, Triticum Vulgare (Wheat) Bran Extract, Actinidia Chinensis (Kiwi) Fruit Extract, Hydrolyzed Elastin, PEG-40 Hydrogenated Castor Oil, Hydrolyzed Keratin, Hydrolyzed Soy Protein , TEA-Dodecylbenzenesulfonate, Guar Hydroxypropyltrimonium Chloride, Amodimethicone, Laureth-23, Citric Acid, PPG-9, Tetrasodium EDTA, Propylene Glycol, Sodium Benzoate, PEG-45M, Magnesium Nitrate, Cetrimonium Chloride, Trideceth-12, Magnesium Chloride, Silica, Methylchloroisothiazolinone, Ethylhexyl Hydroxystearate, Methylisothiazolinone, Ethoxydiglycol, Trideceth-9, Tocopherol, Butylphenyl Methylpropional, Hexyl Cinnamal, Limonene, Linalool, Blue 1 (CI 42090), Red 33 (CI 17200), Yellow 5 (CI 19140), Bismuth Oxychloride (CI 77163), Mica (CI 77019), Titanium Dioxide (CI 77891).	https://www.tigi.com/bedhead/us/product/elasticate-strengthening-shampoo/

Tigi	Recharge High Octane Shine	Water (Aqua/Eau), Sodium Laureth Sulfate, Cocamidopropyl Betaine, Acrylates Copolymer, Fragrance (Parfum), Sodium Chloride, Cyanocobalamin, Biotin, Niacinamide, Panthenol, Pyridoxine HCl, Thiamine HCl, Tocopheryl Acetate, Folic Acid, Capsicum Frutescens Fruit Extract, Carthamus Tinctorius (Safflower) Seed Oil, Menthol, Helianthus Annuus (Sunflower) Seed Oil, Lycium Barbarum Fruit Extract, Benzalkonium Chloride, Ascorbic Acid, Glycerin, Dimethiconol, Phenoxyethanol, TEA-Dodecylbenzenesulfonate, PPG-9, VP/DMAPA Acrylates Copolymer, Guar Hydroxypropyltrimonium Chloride, Citric Acid, Laureth-23, Disodium EDTA, Sodium Benzoate, PEG-45M, Magnesium Nitrate, Propylene Glycol, Silica, Methylchloroisothiazolinone, Retinyl Palmitate, Magnesium Chloride, Methylisothiazolinone, Amyl Cinnamal, Butylphenyl Methylpropional, Hexyl Cinnamal, Limonene, Linalool, Blue 1 (CI 42090), Red 33 (CI 17200), Mica (CI 77019), Titanium Dioxide (CI 77891).	https://www.tigi.com/bedhead/uk/product/recharge-high-octane-shine-shampoo/
Tresemme	Flawless Curls	Water (Aqua), Sodium Laureth Sulfate, Cocamidopropyl Betaine, Sodium Chloride, Dimethiconol, Fragrance (Parfum), Glycerin, Carbomer, Styrene/Acrylates Copolymer, Guar Hydroxypropyltrimonium Chloride, TEA-Dodecylbenzenesulfonate, Citric Acid, Glycol Distearate, Thiamine Hcl, Ascorbic Acid, Tocopheryl Acetate, Panthenol, Niacinamide, Biotin, Cocamide MEA, Tetrasodium EDTA, DMDM Hydantoin, PEG-45M, PPG-9, Polysorbate 20, Methylchloroisothiazolinone, Methylisothiazolinone, Mica (CI 77019), Titanium Dioxide (CI 77891)	http://smartlabel.tresemme.com/product/2745428/nonFoodIngredients?locale=en_us

Tresemme	Moisture Rich	Water (Aqua), Sodium Laureth Sulfate, Cocamidopropyl Betaine, Sodium Chloride, Dimethiconol, Fragrance (Parfum), Glycerin, Carbomer, Styrene/Acrylates Copolymer, Guar Hydroxypropyltrimonium Chloride, Tea-Dodecylbenzenesulfonate, Citric Acid, Glycol Distearate, Tocopheryl Acetate, Ascorbic Acid, Panthenol, Niacinamide, Biotin, Cocamide Mea, Tetrasodium Edta, Dmdm Hydantoin, Peg-45m, Ppg-9, Polysorbate 20, Methylchloroisothiazolinone, Methylisothiazolinone, Mica (Ci 77019), Titanium Dioxide (Ci 77891).	https://www.target.com/p/tresemme-174-moisture-rich-shampoo-28oz/-/A-13074980

Table 3. Conditioner

Brand	Product Name	Ingredients	URL
Axe	Apollo 2 in 1	Water (Aqua), Sodium Laureth Sulfate, Cocamidopropyl Betaine, Sodium Chloride, Dimethiconol, Fragrance (Parfum), Carbomer, Citric Acid, Propylene Glycol, Guar Hydroxypropyltrimonium Chloride, TEA-Dodecylbenzenesulfonate, Tetrasodium EDTA, DMDM Hydantoin, PPG-9, Methylchloroisothiazolinone, Methylisothiazolinone, Mica (CI 77019), Titanium Dioxide (CI 77891), Blue 1 (CI 42090), Yellow 5 (CI 19140)	https://www.amazon.com/Axe-Shampoo-Conditioner-Apollo-12/dp/B00AE07B9O?th=1
Axe	Pheonix 2 in 1	Water (Aqua), Sodium Laureth Sulfate, Cocamidopropyl Betaine, Sodium Chloride, Dimethiconol, Fragrance (Parfum), Carbomer,Propylene Glycol, Citric Acid, Guar Hydroxypropyltrimonium Chloride, TEA-Dodecylbenzenesulfonate, Tetrasodium EDTA, DMDM Hydantoin, PPG-9, Methylchloroisothiazolinone, Methylisothiazolinone, Mica (CI 77019), Titanium Dioxide (CI 77891), Blue 1(CI 42090)	https://www.amazon.com/AXE-Phoenix-Shampoo-Conditioner-Pack/dp/B00604MV0C
Axe	Dual 2 in 1	Water (Aqua), Sodium Laureth Sulfate, Cocamidopropyl Betaine, Sodium Chloride, Dimethiconol, Fragrance (Parfum), Carbomer, Guar Hydroxypropyltrimonium Chloride, Propylene Glycol, TEA-Dodecylbenzenesulfonate, Tetrasodium EDTA, DMDM Hydantoin, Citric Acid, PPG-9, Methylchloroisothiazolinone, Methylisothiazolinone, Mica (CI 77019), Titanium Dioxide (CI 77891), Iron Oxides (CI 77491), Yellow 5 (CI 19140), Red 33 (CI 17200)	https://www.amazon.com/AXE-Dual-Shampoo-Conditioner-Pack/dp/B001FSK8RQ?th=1

Lush	Big	Glyceryl Stearate & PEG-100 Stearate Fine Sea Salt Cetearyl Alcohol & Sodium Lauryl Sulfate Extra Virgin Coconut Oil (Cocos Nucifera) Cetearyl Alcohol Toothed Wrack Seaweed Infusion (Fucus Vesiculosus) Fresh Organic Lemon Juice (Citrus Limonum) Organic Jojoba Oil (Simmondsia Chinensis) Fresh Organic Lime Juice (Citrus Aurantifolia) Propylene Glycol Fragrance Glycerine Guar Gum (Guar Hydroxypropyltrimonium Chloride) Vanilla Absolute (Vanilla Planifolia) Vetivert Oil (Vetiveria Zizanoides) Jasmine Absolute (Jasminum Grandiflorum) Bergamot Oil (Citrus Aurantium Bergamia) Candelilla Wax (Euphorbia Cerifera) Titanium Dioxide Alpha-Isomethyl Ionone Geraniol *Linalool *Coumarin *Limonene	https://www.lushusa.com/hair/conditioners/big/05986.html
Neutrogena	Triple Moisture Daily Deep	Water, Cyclopentasiloxane, Behenyl Alcohol, Behentrimonium Chloride, Cetearyl Alcohol, Cetyl Alcohol, Cyclohexasiloxane, Dimethicone, Amodimethicone, Behentrimonium Methosulfate, Stearamidopropyl Dimethylamine, Cetrimonium Chloride, Panthenol, Sweet Almond (Prunus Amygdalus Dulcis) Oil, Olive (Olea Europaea) Fruit Oil, Meadowfoam (Limnanthes Alba) Seed Oil, Glycol Stearate, Ceteth-2, Hydroxyethylcellulose, Methylparaben, Propylparaben, Citric Acid, Titanium Dioxide, Iron Oxide, Mica, Fragrance.	https://www.neutrogena.com/haircare/conditioner-and-treatment/triple-moisture-daily-deep-conditioner/6802660.html

Table 4. Lotion/Skin Care

Brand	Product Name	Ingredients	URL
Aveeno	Positively Radiant Brightening Body Lotion	Water, mineral oil, glycerin, glycine soja (soybean) seed extract, petrolatum, stearic acid, dimethicone, stearyl alcohol, PEG-100 stearate, glyceryl stearate, phenoxyethanol, fragrance, hydroxyacetophenone, ammonium acryloyldimethyltaurate/VP copolymer, acrylates/C10-30 alkyl acrylate crosspolymer, sodium hydroxide, ethylhexylglycerin, disodium EDTA, silica, mica, titanium dioxide	https://www.aveeno.com/products/positively-radiant-brightening-body-lotion#ingredients
Dove	Cream Oil Intensive Body Lotion	Water, Glycerin, Stearic Acid, Caprylic/Capric Triglyceride, Dimethicone, Glycol Stearate, PDG-100 Stearate, Petrolatum, Cyclopentasiloxane, Acrylates/C10-30 Alkyl Acrylate Crosspolymer, Glyceryl Stearate, Cetyl Alcohol, Fragrance, Phenoxyethanol, Triethanolamine, Carbomer, Methylparaben, Propylparaben, Titanium Dioxide.	https://www.target.com/p/dove-cream-oil-intensive-body-lotion-13-5-oz/-/A-11080203?ref=tgt_adv_XS000000&AFID=google_pla_df&CPNG=PLA_Health+Beauty+Shopping&adgroup=SC_Health+Beauty&LID=700000001170770pgs&network=g&device=c&location=9018949&gclid=CjwKCAjwh9_bBRA_EiwApObaOD1DU-jqr7gFeNkwXmfw_hJnoUdQgtsCSFgImM7aAApyrufWMaAuHBoCef0QAvD_BwE&gclsrc=aw.ds

Dove	Cream Oil Shea Butter	Water, Glycerin, Stearic Acid, Caprylic/Capric Triglyceride, Dimethicone, Glycol Stearate, Peg-100 Stearate, Petrolatum, Butyrospermum Parkii (Shea Butter), Cyclopentasiloxane, Acrylates/C10-30 Alkyl Acrylate Crosspolymer, Glyceryl Stearate, Cetyl Alcohol, Fragrance, Stearamide Amp, Phenoxyethanol, Triethanolamine, Carbomer, Disodium Edta, Methylparaben, Propylparaben, Titanium Dioxide.	https://www.dove.com/us/en/skin-care/body-lotion/cream-oil-shea-butter-body-lotion.html
Dove	DermaSeries Replenishing	Water (Aqua), Glycerin, Dimethicone, Hydroxystearic Acid, Sodium Hydroxypropyl Starch Phosphate, Isopropyl Myristate, Petrolatum, Cetearyl Alcohol, Glyceryl Hydroxystearate, Phenoxyethanol, Caprylyl Glycol, Stearic Acid, Cetearyl Glucoside, Citrus Limon (Lemon) Peel Powder, Citrus Aurantifolia (Lime) Peel Powder, Disodium EDTA, Silk Amino Acids, Ascorbic Acid, Panthenol, Tocopheryl Acetate, Biotin, Niacinamide, Titanium Dioxide (CI 77891).	https://www.dove.com/us/en/skin-care/body-lotion/dermaseries-replenishing-body-lotion.html
Equate	Aloe Cool and Fresh	Water, Glycerin, Stearic Acid, Glycol Stearate, Isopropyl Palmitate, Petrolatum, Aloe Barbadensis Leaf Juice, Cucumis Sativus (Cucumber) Extract, Helianthus Annuus (Sunflower) Seed Oil Or Glycine Soja (Soybean) Oil, Glycine Soja (Soybean) Sterol, Sodium Stearoyl Lactylate, Tocopheryl Acetate (Vitamin E Acetate), Retinyl Palmitate (Vitamin A Palmitate), Panthenol (Provitamin B5), Sodium Acrylate/Sodium Acryloyldimethyl Taurate Copolymer, Dimethicone, Glyceryl Stearate, Cetyl Alcohol, Lecithin, Mineral Water, Sodium Pca, Potassium Lactate, Lactic Acid, Collagen Amino Acids, Urea, Fragrance, Isohexadecane, Polysorbate 80, Triethanolamine, Dmdm Hydantoin, Iodopropynyl Butylcarbamate, Disodium Edta, Titanium Dioxide.	https://www.walmart.com/ip/Equate-Aloe-Cool-Fresh-Lotion-24-5-oz/14520416
Estee Lauder	Resilience Lift	Avobenzone 3.00%; Octisalate 5.00%; Octocrylene 2.70%Ingredients:	https://www.esteelauder.com/pr

		Water\Aqua\Eau; Neopentyl Glycol Diheptanoate; Cetyl Alcohol; Pentaerythrityl Tetraethylhexanoate; Glycerin; Polyethylene; Butyloctyl Salicylate; Di-C12-15 Alkyl Fumarate; Petrolatum; Hydrogenated Lecithin; Butylene Glycol; Propanediol; Hydrogenated Polyisobutene; Octyldodecyl Myristate; Hexyldecyl Stearate; Dimethicone; Glyceryl Stearate; Laminaria Digitata Extract; Behenyl Alcohol; Algae Extract; Persea Gratissima (Avocado) Oil; Coleus Barbatus Extract; Cucumis Melo (Melon) Fruit Extract; Potato Starch Modified; Mimosa Tenuiflora Bark Extract; Yeast Extract\Faex\Extrait De Levure; Plankton Extract; Aminopropyl Ascorbyl Phosphate; Thermus Thermophillus Ferment; Saccharomyces Ferment Lysate Filtrate; Artemia Extract; C12-16 Alcohols; Acetyl Glucosamine; Linoleic Acid; Whey Protein\Lactis Protein\Proteine Du Petit-Lait; Peg-100 Stearate; Tocopheryl Acetate; Caffeine; Ergothioneine; Glycine Soja (Soybean) Sterols; Decarboxy Carnosine Hcl; Palmitic Acid; Creatine; Ethylhexylglycerin; Polymethyl Methacrylate; Trehalose; Glucose; Sodium Hyaluronate; Sorbitol; Zinc Pca; Cholesterol; Malachite; Acetyl Hexapeptide-8; Maltodextrin; Lecithin; Acrylamide/Sodium Acryloyldimethyltaurate Copolymer; Isohexadecane; Sodium Hydroxide; Polysorbate 80; Lactoperoxidase; Hexylene Glycol; Hydrogenated Starch Hydrolysate; Potassium Sulfate; Glucose Oxidase; Polysilicone-11; Propylene Glycol Dicaprylate; Xanthan Gum; Caprylyl Glycol; Citric Acid; Fragrance (Parfum); Bht; Disodium Edta; Phenoxyethanol; Potassium Sorbate; Mica; Titanium Dioxide (Ci 77891); Red 4 (Ci 14700); Yellow 5 (Ci 19140) <ILN39076>	oduct/681/17838/product-catalog/skincare/resilience-lift/firmingsculpting-face-and-neck-creme-spf-15

Hempz	Age Defying	Tocopheryl Acetate, Benzophenone 4, Algae Extract, Ascorbic Acid, Cannabis Sativa Seed Oil (Hemp), Dipalmitoyl Hydroxyproline, Palmitoyl Tetrapeptide 3, Phospholipids, Kola Nut Extract, Caffeine, Triethanolamine, Butylene Glycol, Polysorbate 20, Carbomer, Disodium EDTA, Methylparaben, Propylparaben, Diazolidinyl Urea, Titanium Dioxide, Fragrance (Parfum), Benzyl Benzoate, Coumarin, Hexylcinnamal, Hydroxycitronellal, Hydroxyisohexyl 3 Cyclohexene Carboxaldehyde, Alpha Isomethyl Ionone, Linalool,	https://www.amazon.com/Hempz-Defying-Herbal-Moisturizer-Fluid/dp/B000OYNCES?th=1
Hempz	Pomegranate	Water/Aqua/Eau, Isopropyl Palmitate, Glycerin, Stearic Acid, Sorbitan Palmitate, Butyrospermum Parkii (Shea Butter), Glyceryl Stearate, PEG-100 Stearate, Cetyl Alcohol, Cannabis Sativa Seed Oil, Punica Granatum Extract, Aloe Barbadensis Leaf Juice, Prunus Amygdalus Dulcis (Sweet Almond) Oil, Althaea Officinalis Root Extract, Calendula Officinalis Flower Extract,Symphytum Officinale Leaf Extract, Chamomilla Recutita (Matricaria) Flower Extract, Cucumis Sativus (Cucumber) Fruit Extract, Panax Ginseng Root Extract, Dimethicone, Phenoxyethanol, Nylon-12, Fragrance (Parfum), Polysorbate 40, Aminomethyl Propanol, Carbomer, Disodium EDTA, Titanium Dioxide (CI 77891), Limonene, Benzyl Benzoate, Butylphenyl Methylpropional, Hexyl Cinnamal, Tocopheryl Acetate, Linalool, Hydroxyisohexyl 3-Cyclohexene Carboxaldehyde, Methylisothiazolinone, Benzyl Salicylate, Citronellol, Ascorbic Acid, Retinyl Palmitate, Red 33 (CI 17200), Helianthus Annuus (Sunflower) Seed Oil, Propylene Glycol.	https://www.amazon.com/Hempz-Herbal-Moisturizer-Light-Pomegranate/dp/B0036QFZ46?th=1

Lubriderm	Advanced Therapy	Water, Mineral Oil, Glycerin, Cetyl Alcohol, Sorbitol, Caprylic/Capric Triglyceride, Cetearyl Alcohol, Dimethicone, Polysorbate 60, Stearic Acid, Phenoxyethanol, Lecithin, Carbomer, Sodium Hydroxide, Ceteareth-20, Diazolidinyl Urea, Sodium Citrate, Methylparaben, BHT, Retinyl Palmitate, Sodium Pyruvate, Propylparaben, Fragrance, Citric Acid, Ethylparaben, Panthenol, Tocopheryl Acetate, Titanium Dioxide.	https://www.lubriderm.com/products/advanced-therapy-lotion
Lubriderm	Daily Moisture Sensitive Skin	Water, Mineral Oil, Glycerin, Caprylic/Capric Triglyceride, Cetyl Alcohol, Cetearyl Alcohol, Stearic Acid, Dimethicone, Petrolatum, Ethylhexylglycerin, Carbomer, Ceteareth-20, DMDM Hydantoin, Sodium Hydroxide, Sodium Citrate, Panthenol, Citric Acid, Titanium Dioxide.	https://www.lubriderm.com/products/daily-moisture-lotion-sensitive-skin
Lubriderm	Intensive Skin Repair	Water, Glycerin, Mineral Oil, Caprylic/Capric Triglyceride, Petrolatum, Cetyl Alcohol, Isopropyl Palmitate, Magnesium Aspartate, Zinc Gluconate, Copper Gluconate, Butyrosperum Parkii (Shea Butter), Cetyl Alcohol, Phenoxyethanol, Stearic Acid, Ceteareth-20, Carbomer, Dimethicone, Sodium Hydroxide, Sodium Citrate, Polysorbate 60, Methylparaben, Tetrasodium EDTA, Fragrance, Propylparaben, Xanthan Gum, Citric Acid, Glyceryl Stearate, Ethylparaben, C12-15 Alkyl Benzoate, Polysorbate 80, Glycine Soja (Soybean) Sterols, Titanium Dioxide.	https://www.lubriderm.com/products/intense-skin-repair-lotion
Lubriderm	Men's 3-in-1 Fragrance-Free Lotion	Water, Mineral Oil, Glycerin, Caprylic/Capric Triglyceride, Cetyl Alcohol, Panthenol, Tocopheryl Acetate, Aloe Barbadensis Leaf Extract, Potassium Lactate, Copper Gluconate, Zinc Gluconate, Magnesium Aspartate, Sodium Citrate, Cetearyl Alcohol, Stearic Acid, Dimethicone, Petrolatum, Ethylhexlglycerin, Carbomer, Ceteareth-20, DMDM Hydantoin, Sodium	https://www.lubriderm.com/products/mens-3-1-fragrance-free-lotion

		Hydroxide, Citric Acid, Propylene Glycol, Titanium Dioxide	
Lush	Rump	Cupuacu Butter (Theobroma Grandiflorum) Rose Water (Rosa Damascena) Aloe Vera Gel (Aloe Barbadensis) Glycerine Avocado Butter (Persea Gratissima) Organic Jojoba Oil (Simmondsia Chinensis) Murumuru Butter (Astrocaryum Murumuru) Stearic Acid Triethanolamine Chamomile Blue Oil (Matricaria Chamomilla) Rose Oil (Rosa Damascena) Orange Flower Absolute (Citrus Aurantium Amara) Organic Agave Syrup (Agave Tequilana) Guarana Seed Powder (Paullinia Cupana) Tincture of Benzoin (Styrax Benzoin) Cetearyl Alcohol Butylphenyl Methylpropional Cinnamyl Alcohol *Citral *Citronellol *Eugenol *Geraniol Hydroxyisohexyl 3-Cyclohexene Carboxaldehyde *Limonene *Linalool Fragrance Calcium Sodium Borosilicate Titanium Dioxide Iron Oxides Silica Orange 4 Methylparaben Propylparaben	https://www.lushusa.com/body/body-lotions/rump/07455.html
Lush	Sleepy	Oat Milk (Avena Sativa) Almond Oil (Prunus Dulcis) Lavender Water (Lavandula Angustifolia) Lavender Flower Infusion (Lavandula Angustifolia) Organic Jojoba Oil (Simmondsia Chinensis) Glycerine Fair Trade Organic Cocoa Butter (Theobroma Cacao) Stearic Acid Illipe Butter (Shorea Stenoptera) Triethanolamine Lavender Oil (Lavandula Angustifolia) Benzoin Resinoid (Styrax Tonkinensis Pierre) Tonka Absolute (Dipteryx Odorata) Ylang Ylang Oil (Cananga Odorata) Cetearyl Alcohol *Benzyl Benzoate *Benzyl Cinnamate *Coumarin *Geraniol *Limonene *Linalool Fragrance Synthetic Fluorphlogopite Titanium Dioxide Tin Oxide Red 28 Blue 1	https://www.lushusa.com/body/body-lotions/sleepy/9999906823.html

Michael Kors	A Fabulous Body Lotion	Water, Fragrance, Butylene Glycol, Glycerin, Macadamia Ternifolia Seed Oil, Myristyl Myristate, Glyceryl Stearate, Polysorbate 60, Isocetyl Stearate, C12-15 Alkyl Benzoate, Cetearyl Alcohol, Grape Seed Oil, Avocado Oil, Tridecyl Stearate, Honey Extract, Nylon-12, Silica, Cyclomethicone, Dimethicone, Shea Butter Fruit, Tridecyl Trimelliate, Polyacrylamide, Saccharide, Dipemtaery-Thrityl, Hexacaprylate/Hexacaprate Sodium Hyaluronate, Aloe Vera Gel, Sweet Almond Protein, Hydrolyzed Silk, Hydrolyzed Oat Protein, Calendula Officinalis Flower Extract, Basil Extract, Cypress Leaf Extract, Mullein Extract, Sophora Japonica Flower Extract, Retinyl Palmitate, Tocopheryl Acetate, Linoleic Acid, Linolenic Acid, Arachidyl Propionate, Stearic Acid, Isopropyl Myristate, Ceteareth-20, Octyl Dodecanol, Sodium PCA, Allantoin, Lecithin, C13-14 Isoparaffin, Acrylates/C10-30 Alkyl Acrylate Crosspolymer, Triethanolamine, MIca, Titanium Dioxide, Laureth-7, BHT, Phenoxyethanol, Disodium EDTA, Methylparaben, Propylparaben, FD and C Yellow No. 5, Ext. D and C Violet No. 2, FD and C Red No. 4.	https://www.ulta.com/a-fabulous-body-lotion?productId=prod2068541
Neutrogena	Men Razor Defense	Water, Sodium Cocoyl Isethionate, Stearic Acid, Cetyl Alcohol, Glycerin, Glycol Distearate, Cocamidopropyl Betaine, Sodium Methyl Cocoyl Taurate, Linoleamidopropyl Pg Dimonium Chloride Phosphate, Polyethylene Tocopheryl Acetate, Soybean (Glycine Soja) Seed Extract, Aloe Barbadensis Leaf Extract, C1215 Alkyl Benzoate, Menthyl Lactate, Microcrystalline Wax, Acrylates/C1030 Alkyl Acrylate Copolymer, Xanthan Gum, Ferric Ferrocyanide, Sodium Hydroxide, Disodium Edta, Methylparaben, Ethylparaben, Propylparaben, Phenoxyethanol, Titanium Dioxide, Fragrance.	https://www.amazon.com/Neutrogena-Razor-Defense-Shave-Lotion/dp/B001E96O4C

Olay	Active Hydrating Cream Sensitive	Water, Glycerin, Cetyl Alcohol, Petrolatum, Cyclopentasiloxane, Stearyl Alcohol, Isopropyl Palmitate, Dimethicone, Carbomer, Sodium Hydroxide, PEG-100 Stearate, Fragrance, PEG/PPG-18/18 Dimethicone, Titanium Dioxide, Stearic Acid, Palmitic Acid, EDTA, DMDM Hydantoin, Iodopropynyl Butylcarbamate, Red 4	Water, Sodium Cocoyl Isethionate, Stearic Acid, Cetyl Alcohol, Glycerin, Glycol Distearate, Cocamidopropyl Betaine, Sodium Methyl Cocoyl Taurate, Linoleamidopropyl Pg Dimonium Chloride Phosphate, Polyethylene Tocopheryl Acetate, Soybean (Glycine Soja) Seed Extract, Aloe Barbadensis Leaf Extract, C1215 Alkyl Benzoate, Menthyl Lactate, Microcrystalline Wax, Acrylates/C1030 Alkyl Acrylate Copolymer, Xanthan Gum, Ferric Ferrocyanide, Sodium Hydroxide, Disodium Edta, Methylparaben, Ethylparaben, Propylparaben, Phenoxyethanol

			, Titanium Dioxide, Fragrance.
Olay	Active Hydrating Cream Face Moisturizer	Water, Glycerin, Cetyl Alcohol, Petrolatum, Cyclopentasiloxane, Stearyl Alcohol, Isopropyl Palmitate, Dimethicone, Carbomer, Sodium Hydroxide, PEG-100 Stearate, Fragrance, PEG/PPG-18/18 Dimethicone, Titanium Dioxide, Stearic Acid, Palmitic Acid, EDTA, DMDM Hydantoin, Iodopropynyl Butylcarbamate, Red 4	https://www.olay.com/en-us/skin-care-products/active-hydrating-facial-cream-original
Olay	Total Effects Anti Aging Night Firming Cream	Water, Glycerin, Niacinamide, Isohexadecane, Dimethicone, Isopropyl Isostearate, Stearyl Alcohol, Polyacrylamide, Tocopheryl Acetate, Camellia Sinensis Leaf Extract, Sodium Ascorbyl Phosphate, Panthenol, Cetyl Alcohol, C13-14 Isoparaffin, Behenyl Alcohol, Polymethylsilsesquioxane, Titanium Dioxide, Caprylyl Glycol, 1,2-Hexanediol, Phenoxyethanol, Dimethiconol, Fragrance, Laureth-7, Sodium Benzoate, Peg-100 Stearate, Cetearyl Glucoside, Cetearyl Alcohol, Disodium Edta, Stearic Acid, Palmitic Acid.	https://www.olay.com/en-us/skin-care-products/total-effects-night-firming-treatment

Origin	Matte Moisturizer with Willowherb	Water\Aqua\Eau , Dimethicone , Alcohol Denat. , Glycerin , Silica , Butylene Glycol , Citrus Aurantium Dulcis (Orange) Peel Oil*, Citrus Limon (Lemon) Peel Oil*, Pelargonium Graveolens Flower Oil*, Lavandula Angustifolia (Lavender) Oil*, Amyris Balsamifera Bark Oil*, Citrus Aurantium Bergamia (Bergamot) Fruit Oil*, Eugenia Caryophyllus (Clove) Leaf Oil*, Rosa Damascena Flower Oil*, Eucalyptus Globulus Leaf Oil*, Zingiber Officinale (Ginger) Root Oil*, Mentha Viridis (Spearmint) Leaf Oil*, Limonene, Linalool, Citronellol, Citral, Geraniol, Eugenol , Dimethicone Crosspolymer , Ammonium Acryloyldimethyltaurate/Vp Copolymer , Pyrus Malus (Apple) Fruit Extract , Cucumis Sativus (Cucumber) Fruit Extract , Epilobium Angustifolium Flower\Leaf\Stem Extract , Cistus Incanus Flower/Leaf/Stem Extract , Sucrose** , Bambusa Vulgaris (Bamboo) Extract , Pisum Sativum (Pea) Extract , Castanea Sativa (Chestnut) Seed Extract , Albizia Julibrissin Bark Extract , Salicylic Acid , Caffeine , Arginine , Sodium Hyaluronate , Scutellaria Baicalensis Root Extract , Laminaria Saccharina Extract , Laminaria Digitata Extract , Phospholipids , Hydrolyzed Jojoba Esters , Glucosamine Hcl , Lauryl Peg-9 Polydimethylsiloxyethyl Dimethicone , Dimethicone/Vinyl Dimethicone Crosspolymer , Beeswax\Cera Alba\Cire D'Abeille , Caprylyl Glycol , Acrylates/C10-30 Alkyl Acrylate Crosspolymer , Tocopheryl Acetate , Tromethamine , Silica Silylate , Hexylene Glycol , Sodium Polyacrylate , Disodium Edta , Phenoxyethanol , Mica , Titanium Dioxide (Ci 77891) , Iron Oxides (Ci 77491) * Essential Oil** Organic Sucrose (Brown Sugar) <ILNILN435	https://www.origins.com/product/15352/48069/skincare/moisturize/moisturizers/original-skin/matte-moisturizer-with-willowherb

Physicians Formula	Physicians Formula Super CC+ Color-Correction + Care CC+ Cream	Active Ingredients: Titanium Dioxide 5.76%, Zinc Oxide 2.94% Inactive Ingredients: Water, Cyclopentasiloxane, Caprylic/Capric Triglyceride, Butylene Glycol, Dimethicone, Neopentyl Glycol Diheptanoate, Bis-PEG/PPG-14/14 Dimethicone, Cetearyl Alcohol, C30-45 Alkyl Cetearyl Dimethicone Crosspolymer, PEG-10 Dimethicone, Sodium Chloride, Calcium Aluminum Borosilicate, Glycerin, Teprenone, Hydroxycapric Acid, Hydroxycaprylic Acid, Hydroxycinnamic Acid, Butyrospermum Parkii (Shea) Butter, Tetrahexyldecyl Ascorbate, Tocopheryl Acetate, Caprylyl Glycol , Castor Oil Phosphate, Disodium EDTA, Disteardimonium Hectorite, Ethylhexylglycerin, Glyceryl Stearate SE, Hexylene Glycol, Laureth-12, Mica, PEG/PPG-18/18 Dimethicone, PEG-4, Polysilicone-11, Polysorbate 60, Propylene Carbonate, Sodium Hydroxide, Synthetic Fluorphlogopite, Tin Oxide, Triethoxycaprylylsilane, Xanthan Gum, Phenoxyethanol. May Contain: Iron Oxides, Titanium Dioxide Titanium Dioxid	https://www.amazon.com/Physicians-Formula-Super-Color-Correction-Ounces/dp/B00HUA7UZG/ref=sr_1_1_a_it?ie=UTF8&qid=1538620846&sr=8-1&keywords=physicians%2Bformula%2Bsuper%2Bcc&th=1
Suave	Skin Solutions Body Lotion, Smoothing with Coco Butter & Shea	Water, Glycerin, Stearic Acid, Mineral Oil, Glycol Stearate, Theobroma Cacao (Cocoa) Butter, Butyrospermum Parkii (Shea Butters), Glyceryl Stearate, Cetyl Alcohol, Petrolatum, Fragrance, Dimethicone, Stearamide Amp, Magnesium Aluminum Silicate, Triethanolamine, Carbomer, Propylene Glycol. Methylparaben, Tetrasodium EDTA, DMDM Hydantoin, Caramel, Titanium Dioxide (Cl 77891), Yellow 5 (Cl19140), Yellow 6 (Cl15985).	https://www.amazon.com/Suave-Solutions-Lotion-Smoothing-Butter/dp/B003YNCIKK
Suave	Skin Solutions Body Lotion, Advanced Therapy	Water Glycerin Stearic Acid Glycol Stearate Glyceryl Stearate Triethanolamine Isopropyl Palmitate Cetyl Alcohol DimethiconePetrolatum Magnesium Aluminum Silicate Fragrance DMDM Hydantoin Carbomer Disodium EDTA Methylparaben Iodopropynyl Butylcarbamate Stearamide AMP	https://www.amazon.com/Suave-Solutions-Lotion-Advanced-Therapy/dp/B000PI66S2

		Tocopheryl Acetate Retinyl Palmitate Helianthus Annuus (Sunflower) Seed Oil	
Suave	Skin Solutions, Body Lotion, Advanced Therapy with Rich Hydrators	Water, Glycerin, Stearic Acid, Glycol Stearate, Retinyl Palmitate, Tocopheryl Acetate, Glyceryl Stearate, Cetyl Alcohol, Petrolatum, Fragrance, Dimethicone, Magnesium Aluminum Silicate, Isopropyl Palmitate, Triethanolamine, Carbomer, DMDM Hydantoin, Methylparaben, Disodium EDTA, Iodopropynyl Butylcarbamate, Titanium Dioxide.	http://smartlabel.suave.com/product/2746719/nonFoodIngredients?locale=en-US
Up & Up	Advanced Therapy Lotion	WATER, GLYCERIN, STEARIC ACID, GLYCOL STEARATE, GLYCERYL STEARATE, TRIETHANOLAMINE, ISOPROPYL PALMITATE, CETYL ALCOHOL, DIMETHICONE, PETROLATUM, MAGNESIUM ALUMINUM SILICATE, FRAGRANCE, DMDM HYDANTOIN, CARBOMER, DISODIUM EDTA, METHYLPARABEN, IODOPROPYNYL BUTYLCARBAMATE, STEARAMIDE AMP, TOCOPHERYL ACETATE, RETINYL PALMITATE, HELIANTHUS ANNUUS (SUNFLOWER) SEED OIL, TITANIUM DIOXIDE.	https://www.target.com/p/advanced-therapy-lotion-32oz-up-up-153-compare-to-suave-advanced-therapy-lotion/-/A-13970892
Vaseline	Intensive Care Body Lotion, Cocoa Radiant	Water, Petrolatum, Glycerin, Stearic Acid, Isopropyl Palmitate, Glycol Stearate, Dimethicone, Theobroma Cacao (Cocoa) Seed Butter, Butyrospermum Parkii (Shea Butter), Dihydroxypropyltrimonium Chloride, Hydroxyethyl Urea, Glyceryl Stearate, Cetyl Alcohol, Magnesium Aluminum Silicate, Carbomer, Fragrance, Stearamide Amp, Triethanolamine, Methylparaben, Dmdm Hydantoin, Disodium Edta, Caramel, Titanium Dioxide.	https://www.amazon.com/dp/B01HTJTV4U?aaxitk=f.wTQphFM4JK3scSkKa3TQ&pd_rd_i=B01HTJTV4U&pf_rd_m=ATVPDKIKX0DER&pf_rd_p=54dc821a-0937-4e6f-9da9-f8dd5443145d&pf_rd_s=desktop-sx-top-slot&pf_rd_t=301&pf_rd_i=vaseline%2Bcocoa%2Bbutter%2

			Blotion&hsa_cr_id=5552546170801&sb-ci-n=textLink&sb-ci-v=Vaseline%2BIntensive%2BCare%2BBody%2BLotion%2C%2BCocoa%2BRadiant%2C%2B20.3%2Boz%2C%2B3%2Bct&th=1
Vaseline	Intensive Care Body Lotion, Aloe Soothe	Water (Aqua), Glycerin, Stearic Acid, Isopropyl Myristate, Mineral Oil, Glyceryl Stearate, Glycol Stearate, Dimethicone, Peg-100 Stearate, Petrolatum, Cetyl Alcohol, Tapioca Starch, Phenoxyethanol, Magnesium Aluminum Silicate, Methylparaben, Acrylates/C10-30 Alkyl Acrylate Crosspolymer, Fragrance (Parfum), Propylparaben, Disodium Edta, Xanthan Gum, Stearamide Amp, Aloe Barbadensis Leaf Juice Powder, Titanium Dioxide (Ci 77891)	https://www.amazon.com/Vaseline-Intensive-Care-Lotion-Soothe/dp/B01HTJTV0E/ref=sr_1_3_a_it?ie=UTF8&qid=1538626529&sr=8-3&keywords=vaseline%2Baloe%2Bfresh%2Blotion&th=1
Vaseline	Intensive Care, Cocoa Radiant, Non-Greasy Lotion, With Pure Cocoa Butter	Water (Aqua) Petrolatum Glycerin Stearic Acid Isopropyl Palmitate Glycol Stearate Dimethicone Triethanolamine Glyceryl Stearate Theobroma Cacao (cocoa) Seed Butter Butyrospermum Parkii (Shea) Butter Cetyl Alcohol Fragrance (Parfum) Magnesium Aluminum Silicate Methylparaben DMDM HydantoinCarbomer Disodium EDTA Stearamide AMP Caramel Titanium Dioxide (CI 77891)	http://smartlabel.vaseline.us/product/2745528/nonFoodIngredients?locale=en-us

Vaseline	Intensive Care Essential Healing Lotion	Water (Aqua) Glycerin Stearic Acid Isopropyl Myristate Mineral Oil Glyceryl Stearate Glycol Stearate Dimethicone PEG-100 Stearate Petrolatum Cetyl Alcohol Tapioca Starch Phenoxyethanol Magnesium Aluminum Silicate Methylparaben Acrylates/C10-30 Alkyl Acrylate Crosspolymer Fragrance (Parfum) Propylparaben Disodium EDTA Xanthan Gum Stearamide AMP Avena Sativa (Oat) Straw Extract Titanium Dioxide (CI 77891)	https://www.vaseline.com/us/en/products/lotions-and-moisturizers/vaseline-intensive-care-essential-healing-lotion.html
Vaseline	Men's Body Lotion, Fast Absorbing	Ingredients: Water, glycerin, stearic acid, isopropyl myristate, mineral oil, glyceryl stearate, glycol stearate, dimethicone, PEG-100 stearate, petrolatum, cetyl alcohol, tapioca starch, phenoxyethanol, magnesium aluminum silicate, methylparaben, fragrance, acrylates/C10-30 alkyl acrylate crosspolymer, disodium EDTA, xanthan gum, stearamide AMP, hydroxyethyl urea, titanium dioxide, triethanolamine	https://www.amazon.com/Mountain-Falls-Moisture-Care-Absorbing/dp/B071GKQZJV/ref=sr_1_2_sspa?ie=UTF8&qid=1538627009&sr=8-2-spons&keywords=vaseline+men+fast+absorbing&psc=1

Table 5. Sunscreen

Brand	Product Name	Ingredients	Website URL
Alba Botan ica	Hawaiian Sunscreen	Homosalate 10.0% (sunscreen), octocrylene 10.0% (sunscreen), ethylhecyl methoxycinnamate 7.5% (sunscreen), ethylhexyl salicylate 5.0% (sunscreen), titanium dioxide 2.0% (sunscreen). Inactive ingredients Aqua (water), glycerin, cetearyl olivate, cetyl alcohol, glyceryl stearate SE, sorbitan olivate, dimethicone, VP/hexadecene copolymer, caprylic/capric triglyceride, aloe barbadensis leaf juice*, calendula officinalis flower extract*, camellia sinensis leaf extract*, chamomilla recutita (matricaria) flower extract*, ginkgo biloba leaf extract, lavandula angustifolia (lavender) flower/leaf/stem extract*, sodium stearoyl glutamate, tocopheryl acetate, xanthan gum, alcohol, benzyl alcohol, ethylhecyglycerin, phenoxyethanol, potassium sorbate, ssodium benzoate, citronellol, geraniol, hexyl cinnamal, limonene, linalool and fragrance.	https://www.amazon.com/Alba-Botanica-Hawaiian-Sunscreen-Green/dp/B00CQ7V1JO
Alba Botanica	Very Emollient Sunscreen SPF 30	Active Ingredients: Zinc Oxide 14.5%, Titanium Dioxide 2.0%. Inactive Ingredients: Aqua (Water), Aloe Barbadensis Leaf Juice(1), Caprylic / Capric Triglyceride, Dimethicone, Ethylhexyl Palmitate, Sorbitan Sesquioleate, Glycerin, Butyrospermum Parkii (Shea) Butter, Helianthus Annuus (Sunflower) Seed Oil(1), Simmondsia Chinensis (Jojoba) Seed Oil(1), Polyglyceryl-3 Ricinoleate, Vitis Vinifera (Grape) Seed Oil, Calendula Officinalis Flower Extract(1), Chamomilla Recutita (Matricaria) Flower Extract(1), Magnesium Sulfate, Silica, Sodium Chloride, Tocopheryl Acetate, Benzyl Alcohol, Phenoxyethanol, Polyhydroxystearic Acid. (1) Certified Organic Ingredient.	https://thrivemarket.com/p/alba-botanica-very-emollient-sunscreen-fragrance-free-30-spf?utm_source=google&utm_medium=pla&utm_medium=pla&utm_source=google&utm_campaign=Shopping_Health_Beauty&utm_content=724742003890&utm_term=google-pla_na&gclid=

			CjwKCAjwsdfZBRAkEiwAh2z65n971_98M3FvBgbhzDA5TAozR_T7zNWFaKPNTUZDWYRDefIBBMGgVxoC2eIQAvD_BwE
Avene	Antirougeurs Day Redness-Relief Soothing Cream SPF 25	Active: Octocrylene 4%, Octinoxate 7.5%, Titanium Dioxide 4.5%.	https://www.dermstore.com/product_Antirougeurs+Day+RednessRelief+Soothing+Cream+SPF+25_16753.htm
Avene	Complexion Correcting Shield SPF 50+	Active: Titanium Dioxide 13.2%, Zinc Oxide 15.6%. Inactive: Water, Caprylic / Capric Triglyceride, Hydrogenated Polyisobutene, Dimethicone, Butyloctyl Salicylate, Isodecyl Neopentanoate, Polyhydroxystearic Acid, C12-15 Alkyl Benzoate, Magnesium Aluminum Silicate, Polymethyl Methacrylate, Potassium Cetyl Phosphate, Vp/Eicosene Copolymer, Polysorbate 20, Soyethyl Morpholinium Ethosulfate, Iron Oxides, Trimethylsiloxysilicate, Cetearyl Alcohol, Xanthan Gum, Glycerin, Phenoxyethanol, Hydrogenated Lecithin, Stearic Acid, Peg/PPPG/Polybutylene Glycol-8/5/3 Glycerin, Carbomer, Ceramide Ap, Ceramide EOP, Ceramide NP, Phytosphingosine, Cholesterol, Sodium Lauroyl Lactylate, Tocopherol, Sodium Hyaluronate, Ethylhexylglycerin, Disodium Edta, Tocopheryl Acetate, Allantoin, Ascorbic Acid.	https://www.dermstore.com/product_Complexion+Correcting+Shield+SPF+50++Medium_70741.htm

Avene	MINERAL Light Mattifying Sunscreen Lotion SPF 50+ (Face)	ACTIVE INGREDIENTS:TITANIUM DIOXIDE (5.5%), ZINC OXIDE (8%).<br><br>INACTIVE INGREDIENTS: WATER, C12-15 ALKYL BENZOATE, BUTYLOCTYL SALICYLATE, GLYCERIN, SILICA, STYRENE ACRYLATES COPOLYMER, ISOSTEARYL ISOSTERATE, ISONONYL ISONONANOATE, POLYESTER-7, NEOPENTYL GLYCOL DIHEPTANOATE, DICAPRYLYL CARBONATE, STEARETH-100, PHENOXYETHANOL, SORBITAN ISOSTEARATE, POLYGLYCERYL-3 DISTEARATE, P-ANISIC ACID, GLYCERYL LAURATE, CAPRYLOL GLYCINE, STEARETH-2, POLYACTYLAMIDE, ARACHIDYL ALCOHOL, C13-14 ISOPARAFFIN, SORBITOL, BEHENYL ALCOHOL, ZINC GLUCONATE, TOCOPHERYL, ISOSTEATIC ACID, POLYHYDROXYSTEARIC ACID, ALUMINA, METHICONE, ETHYLHEXYLGLYCERIN, SARCOSINE, DISODIUM EDTA, ARACHIDYL GLUCOSIDE, MANNAN, XANTHAN GUM, CINNAMOMUM ZEYLANICUM BARK EXTRACT, LAURETH-7, LECITHIN, IRON OXIDE, OLEIC ACID.	https://www.aveneusa.com/catalog/product/view/id/39987/s/mineral-light-mattifying-sunscreen-lotion-spf-50/category/585/
Avene	Hydrance Optimale SPF 25	octocrylene (8%), Octinoxate (7.5%), Titanium Dioxide (3.2%), water (Aqua), Cyclomethicone, Glycerin, Ethylhexyl Palmitate, Stearyl Alcohol, Potassium Cetyl Phosphate, Glyceryl Stearate, Alumina, Benzoic Acid, Beta-sitosterol, Caprylic/Capric Triglyceride, Chlorphenesin, Disodium EDTA, Glycine Soja (Soybean) Seed Extract (Glycine Soja), Hydroxyethyl Acrylate/ Sodium Acryloyldimethyl Taurate Copolymer, Phenoxyethanol, Poloxamer 188,	https://www.dermstore.com/product_Hydrance+Optimale+SPF+25_16284.htm

		Polysorbate 60, Simethicone, Sodium Chondroitin Sulfate, Squalane, Tocopheryl Glucoside, Xanthan Gum.	
Aveeno	Active Naturals Natural Protection SPF 50 Lotion	Active Ingredients: Titanium Dioxide (6%), Zinc Oxide (3%). Purpose: Sunscreen. Inactive Ingredients: Water, C12-15 Alkyl Benzoate, Cetyl Dimethicone, Styrene/Acrylates Copolymer, PEG-8, Beeswax, Dimethylimidazolidinone Rice Starch, Polyamide-5, Butylene Glycol, Ethylhexylglycerin, Glyceryl Stearate, PEG-100 Stearate, Benzyl Alcohol, Dimethicone, Polyhydroxystearic Acid, Arachidyl Alcohol, Caprylyl Glycol, Trisiloxane, Dipropylene Glycol Dibenzoate, Glycerin, Pentylene Glycol, Behenyl Alcohol, Triethoxycaprylylsilane, Xanthan Gum, Citric Acid, Bisabolol, Acrylates/Dimethicone Copolymer, Arachidyl Glycoside, PPG-15 Stearyl Ether Benzoate, Chlorphenesin, Hydroxyethyl Acrylate/Sodium Acryloyldimethyl Taurate Copolymer, Squalane, Dipotassium Glycyrrhizate, Polyaminopropyl Biguanide, Sodium Citrate, Hydroxyphenyl Propamidobenzoic Acid, Polysorbate 60, Avena Sativa (Oat) Kernel Extract.	https://www.amazon.com/AVEENO-Active-Naturals-Natural-Protection/dp/B01774S9LI/ref=sr_1_1_sspa?ie=UTF8&qid=1537640787&sr=8-1-spons&keywords=aveeno+spf+50&psc=1&smid=ASEVS99O6FS73
Aveeno	Postively Radiant Daily Moisturizer Broad Spectrum SPF 15	Active Ingredients: Avobenzone 3%, octinoxate 7.5%, octisalate 2%. Inactive Ingredients: Water, C12-15 alkyl benzoate, cetearyl alcohol, dimethicone, glycine soja (soybean) seed extract, glycerin, bis-phenylpropyl dimethicone, arachidyl alcohol, phenoxyethanol, cetearyl glucoside, panthenol, benzyl alcohol, ethylene/acrylic acid copolymer, behenyl alcohol, steareth-2, fragrance, steareth-21, polyacrylamide, polymethyl methacrylate, arachidyl glucoside, disodium EDTA, methylparaben, C13-14 isoparaffin, ethylparaben, butylparaben, laureth-7, benzalkonium chloride, propylparaben, isobutylparaben, iodopropynyl butylcarbamate, BHT, titanium dioxide,	https://www.aveeno.com/products/positively-radiant-daily-moisturizer-spf-15#ingredients

		mica, silica.<br><br>Inactive Ingredients: Water, C12-15 alkyl benzoate, cetearyl alcohol, dimethicone, glycine soja (soybean) seed extract, glycerin, bis-phenylpropyl dimethicone, arachidyl alcohol, phenoxyethanol, cetearyl glucoside, panthenol, benzyl alcohol, ethylene/acrylic acid copolymer, behenyl alcohol, steareth-2, fragrance, steareth-21, polyacrylamide, polymethyl methacrylate, arachidyl glucoside, disodium EDTA, methylparaben, C13-14 isoparaffin, ethylparaben, butylparaben, laureth-7, benzalkonium chloride, propylparaben, isobutylparaben, iodopropynyl butylcarbamate, BHT, titanium dioxide, mica, silica.	
Aveeno	Baby Natural Protection Lotion Sunscreen, SPF 50	Active Ingredients: TITANIUM DIOXIDE 6.0%, ZINC OXIDE 3.0%; Inactive Ingredients: WATER, C12-15 ALKYL BENZOATE, CETYL DIMETHICONE, STYRENE/ACRYLATES COPOLYMER, PEG-8, BEESWAX, DIMETHYLIMIDAZOLIDINONE RICE STARCH, POLYAMIDE-5, BUTYLENE GLYCOL, ETHYLHEXYLGLYCERIN, GLYCERYL STEARATE, PEG-100 STEARATE, BENZYL ALCOHOL, DIMETHICONE, POLYHYDROXYSTEARIC ACID, ARACHIDYL ALCOHOL, CAPRYLYL GLYCOL, TRISILOXANE, DIPROPYLENE GLYCOL DIBENZOATE, GLYCERIN, PENTYLENE GLYCOL, BEHENYL ALCOHOL, TRIETHOXYCAPRYLYLSILANE, XANTHAN GUM, CITRIC ACID, BISABOLOL, ACRYLATES/DIMETHICONE COPOLYMER, ARACHIDYL GLUCOSIDE, PPG-15 STEARYL ETHER BENZOATE, CHLORPHENESIN, HYDROXYETHYL ACRYLATE/SODIUM	https://www.ewg.org/sunscreen/about-the-sunscreens/785109/Aveeno_Baby_Natural_Protection_Lotion_Sunscreen%2C_SPF_50/#.W2O6pNJKhPZ

		ACRYLOYLDIMETHYL TAURATE COPOLYMER, SQUALANE, DIPOTASSIUM GLYCYRRHIZATE, POLYAMINOPROPYL BIGUANIDE, SODIUM CITRATE, HYDROXYPHENYL PROPAMIDOBENZOIC ACID, POLYSORBATE 60, AVENA SATIVA (OAT) KERNEL EXTRACT.	
Babyganics	Baby Sunscreen Lotion, SPF 50	Octisalate - (5.0%), Titanium Dioxide - (3.0%), Zinc Oxide - (6.0%) Water, Butyloctyl Salicylate, Neopentyl Glycol Diethylhexanoate, Caprylic/Capric Triglyceride, Stearyl/Octyldodecyl Citrate Crosspolymer, Polyglyceryl-2 Stearate, Simmondsia Chinensis (Jojoba) Seed Oil*, Hydrogenated Dimer Dilinoleyl/Dimethylcarbonate Copolymer, Glyceryl Stearate, Cetyl Alcohol, Cocos Nucifera (Coconut) Oil*, Glycerin*, Solanum Lycopersicum (Tomato) Seed Oil, Helianthus Annuus (Sunflower) Seed Oil*, Vaccinium Macrocarpon (Cranberry) Seed Oil, Nigella Sativa (Black Cumin) Seed Oil*, Rubus Idaeus (Red Raspberry) Seed Oil, Butyrospermum Parkii (Shea) Butter*, Jojoba Esters, Stearyl Alcohol, Arachidyl Alcohol, Arachidyl Glucoside, Behenyl Alcohol, Polyhydroxystearic Acid, Xanthan Gum, Silica, Alumina, Ethylhexylglycerin, Trisodium Ethylenediamine Disuccinate, Phenoxyethanol *certified organic ingredient	https://www.amazon.com/Babyganics-Baby-Sunscreen-Lotion-Tube/dp/B00HYV2F7E/ref=sr_1_5?hvadid=177580100103&hvdev=c&hvlocphy=9018948&hvnetw=g&hvpos=1t2&hvqmt=b&hvrand=134579519780380476544&hvtargid=kwd-86608603749&keywords=babyganics%2Bsunscreen%2Bspf%2B50&qid=1550883561&s=gateway&sr=8-5&tag=googhydr-20&th=1
Banana Boat	Simply Protect Baby Lotion - SPF 50	Titanium Dioxide 4.5%, Zinc Oxide 6.5%. Inactive Ingredients: Water, Caprylic/Capric Triglyceride, Isohexadecane, Butyloctyl Salicylate, Octyldodecyl Citrate Crosspolymer, Cetyl PEG/PPG-10/1 Dimethicone, Lauryl PEG-8 Dimethicone, C30-38 Olefin/Isopropyl Maleate/MA Copolymer, Sodium Chloride, Ethylhexyl Methoxycrylene, Dimethicone, Phenoxyethanol, Caprylyl Glycol, PEG-8,	https://www.cvs.com/shop/banana-boat-simply-protect-baby-spf-50-lotion-6-oz-prodid-1780003?skuid=222735

		Alumina, Glycerin, Sodium Citrate, Tocopheryl Acetate	
Banana Boat	Kids Sport Sunscreen Lotion - SPF 50	Titanium Dioxide (4.5%) Zinc Oxide (6.5%), Water, Caprylic/Capric Triglyceride, Isohexadecane, Butyloctyl Salicylate, Octyldodecyl Citrate Crosspolymer, Cetyl Peg/Ppg-10/1 Dimethicone, Lauryl Peg-8 Dimethicone, C30-38 Olefin/Isopropyl Maleate/Ma Copolymer, Sodium Chloride, Ethylhexyl Methoxycrylene, Dimethicone, Phenoxyethanol, Caprylyl Glycol, Peg-8, Alumina, Glycerin, Sodium Citrate, Methyl Dihydroabietate, Phenylisopropyl Dimethicone, Polyglyceryl-3 Stearate/Isostearate/Dimer Dilinoleate Crosspolymer, Tocopheryl Acetate, Aloe Barbadensis Leaf Juice.	https://www.target.com/p/banana-boat-kids-sport-sunscreen-lotion-spf-50-6oz/-/A-51163796
Bare Republic	Mineral Sport Lotion - SPF50	Active Ingredients: Titanium Dioxide (5.6%), Zinc Oxide (3.7%). Inactive ingredients Alcohol,Aleurites Moluccana Seed Oil,Algae Extract,Allyl Heptanoate,Aloe Barbadensis Leaf,Aluminum Hydroxide,Beeswax,Butyloctyl Salicylate,Caprylic/Capric Triglyceride,Carica Papaya (Papaya) Fruit Extract,Carthamus Tinctorius (Safflower) Seed Oil,Cetyl Alcohol,Citric Acid,Cocos Nucifera (Coconut) Oil,Dicaprylyl Carbonate,Ethylhexylglycerin,Gamma Decalactone,Gamma-Nonalactone,Gamma Octalactone,Glycerin,Heliotropine,Hydrogen Dimethicone,Olea Europaea (Olive) Fruit Extract,Olea Europaea (Olive) Fruit Oil,Phenoxyethanol,Polyethylene,Propanediol,Raspberry Ketone,Steareth-2,Steareth-21,Triethoxycaprylylsilane,Triethyl Citrate,Trisodium Ethylenediamine Disuccinate,Vanillin,Water,Xanthan Gum.	https://www.target.com/p/bare-republic-mineral-body-lotion-spf50-3-4oz/-/A-16576941

Blue Lizard	Australian Sunscreen, Sensitive, SPF 30+ Fragrance Free	Active Ingredients: Titanium Dioxide (5%), Zinc Oxide (10%)<br><br>Inactive Ingredients: Water, Ethylhexyl Palmitate, C12 15 Alkyl Benzoate, Ethylhexyl Stearate, Polyglyceryl 4 Isostearate, Hexyl Laurate, Trimethylated Silica/Dimethicone, Octododecyl Neopentanoate, VP/Hexadecene Copolymer, Methyl Glucose Dioleate, PEG 7 Hydrogenated Castor Oil, Hydrogenated Castor Oil, Beeswax, Stearic Acid, Disodium EDTA, Tocopheryl Acetate, Caprylyl Glycol, Cetyl Dimethicone Copolymer, Chlorphenisen, Phenoxyethanol, Propandeiol, Sorbitan Oleate	https://www.walgreens.com/store/c/blue-lizard-australian-sunscreen-sensitive-spf-30%2B-fragrance-free/ID=prod6064741-product?ext=gooPLA_-_Beauty&ext=gooKBM_PLA_-_Personal_Care&pla&adtype=pla_with_promotion&kpid=sku6059499&sst=d7b66d27-d128-43ac-ae4f-975c4752f8cb
Burt's Bees	Day Lotion, with Royal Jelly, Broad Spectrum SPF 15	Active Ingredients: Titanium Dioxide (6.7%). Purpose: Sunscreen. Inactive Ingredients: Water, Isoamyl Laurate, Decyl Oleate, Isoamyl Cocoate, Glycerin, Caprylic/Capric Triglyceride, Polyglyceryl-2 Dipolyhydroxystearate, Stearic Acid, Polyglyceryl-4 Caprate, Royal Jelly, Lonicera Japonica (Honeysuckle) Flower Extract, Lonicera Caprifolium (Honeysuckle) Flower Extract, Calendula Officinalis Flower Extract, Hydrastis Canadensis (Goldenseal) Extract, Hamamelis Virginiana (Witch Hazel) Leaf Extract, Beeswax, Helianthus Annuus (Sunflower) Seed Oil, Cetyl Alcohol, Glyceryl Stearate Citrate, Sodium Carboxymethyl C10-16 Alkyl Glucoside, Sorbitan Stearate, Behenyl Alcohol, Stearyl Alcohol, Lauryl Glucoside, Tocopherol, Alumina, Magnesium Aluminum Silicate, Xanthan Gum, Isostearic Acid, Lecithin, Polyglyceryl-3 Polyricinoleate, Polyhydroxystearic Acid, Citric Acid, Glycine Soja (Soybean) Oil,	https://express.google.com/u/0/product/3257672611826265261_11482416689994880233_3748817?utm_source=google_shopping&utm_medium=tu_cu&utm_content=eid-lsjeuxoeqt,eid-4160052&gtim=CK3nkbO-8d2kmQEQqK_GvOKg29VuGPCCyggiA1VTRA&utm_campaign=3748817

		Sodium Phytate, Sorbic Acid, Alcohol, Fragrance (Natural Fragrance), Phenoxyethanol, Benzyl Salicylate, Citronellol, Limonene, Linalool.	
CeraVe	Face Sunscreen Lotion, Lightweight and Oil-Free, SPF 50	Active Ingredients: Titanium Dioxide (4.9%), Zinc Oxide (4.7%). Purpose: Sunscreen, Sunscreen. Inactive Ingredients: Water, Butyloctyl Salicylate, Cetyl Dimethicone, Dimethicone, Styrene/Acrylates Copolymer, Trimethylsiloxysilicate, Arachidyl Alcohol, Arachidyl Glucoside, Trisiloxane, Dimethicone, PEG-8 Laurate, Isohexadecane, Butylene Glycol, Polysorbate 60, Polyhydroxystearic Acid, Silica, Ceramide 3, Ceramide 6-II, Ceramide 1, Cholesterol, Niacinamide, Phytosphingosine, PEG-100 Stearate, Glyceryl Stearate, Ascorbic Acid, Avena Sativa (Oat) Kernel Extract, Beeswax, Behenyl Alcohol, Benzyl Alcohol, Stearic Acid, Bisabolol, Dipotassium Glycyrrhizate, Ethylhexylglycerin, Glycerin, Hydroxyethyl Acrylate/Sodium Acryloyldimethyl Taurate Copolymer, Pantothenic Acid/Yeast Polypeptide, PEG-8, Xanthan Gum, Polyaminopropyl Biguanide, Polymethyl Methacrylate, Potassium Sorbate, Retinyl Palmitate, Sodium Lauroyl Lactylate, Carbomer, Tocopheryl Acetate BHT, Disodium EDTA, Methicone, Methylisothiazolinone, Triethoxycaprylylsilane.	https://www.walmart.com/ip/CeraVe-Face-Sunscreen-Lotion-Lightweight-and-Oil-Free-SPF-50-3-5-oz/23623658?wmlspartner=wlpa&adid=22222222227017153544&wl0=&wl1=g&wl2=c&wl3=40842981872&wl4=pla-78656545352&wl5=9018949&wl6=&wl7=&wl8=&wl9=pla&wl10=8175035&wl11=online&wl12=23623658&wl13=&veh=sem
Coola	Mineral Face SPF 30 Matte Tint Moisturizer	Active Ingredients: Titanium Dioxide 3.2%, Zinc Oxide 1.8%	https://coolasuncare.com/collections/sunscreen-face/products/mineral-face-spf-30-unscented-matte-finish-tint-sunscreen-lotion

Equate	Kids Sunscreen Lotion, SPF 50	Ingredients: Active Ingredients: Homosalate 15%, Octinoxate 7.5%, Octisalate 5%, Titanium Dioxide 2.3%. Purpose: Sunscreen. Inactive Ingredients: Water, Isohexadecane, Ozokerite, Cetyl Peg/PPG-10/1 Dimethicone, Dibutyl Adipate, C30-38 Olefin/Isopropyl Maleate/Ma Copolymer, PEG-8, Glycerin, Sodium Citrate, Sodium Chloride, Aloe Barbadensis (Aloe Vera) Leaf Juice, Tocopheryl Acetate (Vitamin E), Sodium Ascorbyl Phosphate, Retinyl Palmitate, Dimethicone, Phenoxyethanol, Methylparaben, Butylparaben, Ethylparaben, Propylparaben, Isobutylparaben, Disodium EDTA, Alumina.	https://www.walmart.com/ip/Equate-Kids-Sunscreen-Lotion-SPF-50-8-Fl-Oz/14122654
Estee Lauder	Day Wear	Active: Avobenzone 3%, Homosalate 5%, Octisalate 5%, Octocrylene 2.7%, Oxybenz one 3%. Inactive: Water, Butyloctyl Salicylate, Methyl Trimethicone, Neopentyl Glycol Diheptanoate, Aleurites Moluccana (Kukui) Seed Oil, Lauryl PEG-9 Polydimethylsiloxyethyl Dimethicone, PEG-10 Stearate, Butylene Glycol, Glyceryl Stearate, Dipentaerythrityl Tri-Polyhydroxystearate, Potassium Cetyl Phosphate, Gentiana Lutea (Gentia) Root Extract, Hordeum Vulgare (Barley) Extract, Triticum Vulgare (Wheat) Germ Extract, Fumaria Officinalis Flower/Leaf/Stem Extract, Citrus Medica Limonum (Lemon) Fruit Extract, Hydrolyzed Rice Bran Extract, Hydrolyzed Rice Extract, Rosmarinus Officinalis (Rosemary) Leaf Extract, Vitis Vinifera (Grape) Seed Extract, Artemia Extract, Laminaria Ochroleuca Extract, Algae Extract, Hydrogenated Lecithin, Thermus Thermophillus Ferment, Squalane, Glyercin, Sorbitol, Linoleic Acid, Caffeine, Cholesterol, Ethyhexylglycerin, Lecithin, Ergothioneine, Sodium Hyaluronate, Polyquaternium-51, Cetyl Alcohol, Trehalose, Palmitoyl Hydroxypropyltrimonium	https://www.futurederm.com/estee-lauder-daywear-advanced-multi-protection-anti-oxidant-uv-defense-broad-spectrum-spf-50-spf-50/

		Amylopectin/Glycerin Crosspolymer, Ammonium Acryloyldimethyltaurate/VP Copolymer, VP/Eicosene Copolymer, Tetrahexyldecyl Ascorbate, Saccharide Isomerate, Caprylyl Glycol, Caprylic/Capric Triglyceride, Sodium PCA, Cyclodextrin, Urea, Dehydroxanthan Gum, Tocopheryl Acetate, Glycyrrhetinic Acid, Nordihydroguaiaretic Acid, Ascrobyl Tocopheryl Maleate, Citric Acid, Stearic Acid, Sodium Dehydroacetate, Fumaric Acid, Ethylbisiminomethylguaiacol Manganese Chloride, Silica, Phenoxyethanol, Disodium EDTA, BHT, Fragrance, Mica, Titanium Dioxide, Blue 1, Yellow 5.	
Eucerin	Daily Protection Broad Spectrum SPF 30 Sunscreen Moisturizing Face Lotion	Inactive Ingredients: Alumina;VP/Hexadecene Copolymer;Glyceryl Stearate SE;DMDM Hydantoin;Cylclomethicone;Phenoxyethanol;PEG-40 Castor Oil;Sodium Lactate;Simethicone;Hydrogenated Coco-Glycerides;Cetearyl Alcohol;Ceteareth-20;Sodium Hydroxide;C-12-15 Alkyl Benzoate;Xanthan Gum;Trisodium EDTA;Water;Glycerin;Sodium Cetearyl Sulfate;Cetyl Alcohol;Lactic Acid;Dimethicone<br><br>Ingredients: Active: Ensulizole 2.0%...Purpose: Sunscreen, Octinoxate 7.5%...Purpose: Sunscreen, Octisalate 4.5%...Purpose: Sunscreen, Titanium Dioxide 2.38%...Purpose: Sunscreen, Zinc Oxide 4.85%...Purpose: Sunscreen. Inactive: Water, Glycerin, C-12-15 Alkyl Benzoate, Dimethicone, Cylclomethicone, Cetyl Alcohol, Cetearyl Alcohol, Ceteareth-20, Glyceryl Stearate SE, Soduim Lactate, Lactic Acid, Hydrogenated Coco-Glycerides, VP/Hexadecene Copolymer, PEG-40 Castor Oil, Sodium Cetearyl Sulfate, Xanathan Gum, Trisodium EDTA, Sodium	https://www.walmart.com/ip/Eucerin-Daily-Protection-Broad-Spectrum-SPF-30-Sunscreen-Moisturizing-Face-Lotion-4-fl-oz/10810941

		Hydroxide, Alumina, Simethicone, Phenoxyethanol, DMDM Hydantoin.<br><br>Active Ingredients: Titanium Dioxide; Octisalate; Zinc Oxide; Ensulizole; Octinoxate	
Goddess Garden Organics	SPF 30 Sport Natural Mineral Sunscreen Continuous Spray for Sensitive Skin	Active Ingredients: Titanium Dioxide 6.4%, Zinc Oxide 6.0% Inactive Ingredients: Aloe Vera*, Camellia Sinensis (Green Tea), Caprylic/Capric Triglyceride (Coconut Oil), Caprylyl/Capryl Glucoside, Glycerin*, Glyceryl Caprylate, Glyceryl Stearate Citrate, Glyceryl Undecylenate, Helianthus Annus (Sunflower) Oil*, Lecithin (Sunflower), Polyhydroxystearic Acid, Radish Root Ferment Filtrate, Rubus Idaeus (Red Raspberry) Seed Oil, Stearic Acid, Tocopherol (Vitamin E), Xanthan Gum (*CERTIFIED ORGANIC)	https://www.amazon.com/Goddess-Garden-Sunscreen-Continuous-Cruelty-Free/dp/B00JHN8AZ8?th=1
Goddess Garden Organics	Goddess Garden Organics Sunny Kids SPF 30 Natural Sunscreen SPF	Active Ingredients: Titanium Dioxide (6.4%), Zinc Oxide (6.0%). Purpose: Sunscreen. Inactive Ingredients: Aloe Vera (Certified Organic), Butyrospermum Parkii (Shea Butter) (Certified Organic), Camellia Sinensis (Green Tea), Caprylic/Capric Triglyceride (Coconut Oil), Caprylyl/Capryl Glucoside, Glycerin (Certified Organic), Glyceryl Caprylate, Glyceryl Stearate Citrate, Glyceryl Undecylenate, Helianthus Annus (Sunflower) Oil (Certified Organic), Lavandula Angustifolia (Lavender) Oil, Lecithin (Sunflower), Polyhydroxystearic Acid, Radish Root Ferment Filtrate, Rubus Idaeus (Red Raspberry) Seed Oil, Stearic Acid, Tocopherol (Vitamin E), Xanthan Gum.	https://www.walmart.com/ip/Goddess-Garden-Organics-Sunny-Kids-SPF-30-Natural-Sunscreen-SPF-6-Oz/37569951?adid=22222222254418684945&wmlspartner=wmtlabs&wl0=b&wl1=g&wl2=c&wl3=291938340609&wl4=dsa-504748227745&wl5=9018944&wl6=&wl7=&wl8=&veh=sem&gclid=CjwKCAjw0JfdBRACEiwAiDTALt-VANhfu58SwdngWu_GeO8sb

			5fByzh5xC4lLeymveBlDB62xTgo9RoCncAQAvD_BwE
Goddess Garden Organics	SPF 30 Everyday Natural Mineral Sunscreen Lotion for Sensitive Skin	Active Ingredients: Titanium Dioxide 6.4%, Zinc Oxide 6.0% Inactive Ingredients: Aloe Vera, Butyrospermum Parkii (Shea Butter), Caprylic/Capric Triglyceride (Coconut Oil), Glycerin, Glyceryl Caprylate, Glyceryl Stearate, Glyceryl Stearate Citrate, Glyceryl Undecylenate, Glycine Soja (Soybean) Oil, Helianthus Annus (Sunflower) Oil, Iron Oxide, Lavendula Augustifolia (Lavender) Oil, Polyhydroxystearic Acid, Radish Root Ferment Filtrate, Stearic Acid, Tocopherol (Vitamin E), Xanthan Gum Certified Organic	https://www.amazon.com/Goddess-Garden-Natural-Sunscreen-Cruelty-Free/dp/B002BQ351M?th=1
Hawaiian Tropic	Sunscreen Silk Hydration	Water,Isohexadecane,Diisopropyl Adipate,Ethylhexyl Benzoate,Butylene Glycol,Hydroxyethyl Acrylates/Sodium Acryloyldimethyl Taurate Copolymer,Cetearyl Alcohol,Acrylates/C12-22 Alkyl Methacrylate Copolymer,Phenoxyethanol,Caprylyl Glycol,Fragrance,Methylparaben,Ceteth-10 Phosphate,Dicetyl Phosphate,Propylparaben,Polysorbate 60,Disodium EDTA,Xanthan Gum,Aminomethyl Propanol,Mica,Tocopheryl Acetate,Titanium Dioxide,Panthenol,Aloe Barbadensis Leaf Juice,Silk Amino Acids,Sodium Ascorbyl Phosphate,Carica Papaya (Papaya) Fruit Extract,Colocasia Antiquorum Root Extract,Mangifera Indica (Mango) Fruit Extract,Passiflora Incarnata Fruit Extract,Plumeria Acutifolia Flower Extract,Psidium Guajava Fruit Extract,Iron Oxides.	https://www.target.com/p/hawaiian-tropic-174-silk-hydration-lotion-sunscreen-spf-15-5-1-fl-oz/-/A-21402667?ref=tgt_adv_XS000000&AFID=google_pla_df&CPNG=PLA_Health+Beauty+Shopping&adgroup=SC_Health+Beauty&LID=700000001170770pgs&network=g&device=c&location=9018948&gclid=CjwKCAjwoMPcBRAWEiwAiAqZh_pQWZE7mqxdQ_qnet5ZazsedDOlNqPhydrSY2Wf35lFd8cS1FI

			LDxoCMqoQAvD_BwE&gclsrc=aw.ds
Kiehl's	Super Fluid UV Mineral Defense Broad Spectrum SPF 50+	Active Ingredients: Titanium Dioxide-15.00%	https://www.kiehls.com/skincare/sunscreen/super-fluid-uv-mineral-defense-broad-spectrum-spf-50/KHL416.html
La Roche-Posay	Anthelios 50-Day-Light Protection Sun Screen SPF 50	Active Ingredients: Titanium Dioxide (11%). Inactive Ingredients: Water, Isododecane, C12-15 Alkyl Benzoate, Dimethicone, Undecane, Triethylhexanoin, Isohexadecane, Styrene/Acrylates Copolymer, Nylon-12, Caprylyl Methicone, Butyloctyl Salicylate, Phenethyl Benzoate, Silica, Tridecane, Dicaprylyl Carbonate, Dicaprylyl Ether, Talc, Dimethicone/PEG-10/15 Crosspolymer, Aluminum Stearate, Pentylene Glycol, C9-15 Fluoroalcohol Phosphate, Tocopherol, Phenoxyethanol, Stearic Acid, Diethylhexyl Syringylidenemalonate, PEG-9 Polydimethylsiloxyethyl Dimethicone, Magnesium Sulfate, PEG-8 Laurate, PEG-9, Polyhydroxystearic Acid, Maltodextrin, Benzoic Acid, Iron Oxides, Propylene Glycol, Propylene Carbonate, Caprylyl Glycol, Disteardimonium Hectorite, Cassia Alata Leaf Extract, Alumina, Aluminum Hydroxide.	https://express.google.com/u/0/product/4342059515879451216_1613583068203151816_6696654?utm_source=google_shopping&utm_medium=tu_cu&utm_content=eid-lsjeuxoeqt,eid-4160052&gtim=CO3c4c7gzbq8KBCnxvOdzbqJq7UBGODW_A8iA1VTRCiQqdDdBQ&utm_campaign=6696654
Lotus Herbals	Safe Sun 3-in-1 Matte Look Daily Sunblock SPF 40	Key ingredients: Octyl salicylate, Benzophenone-3, Avobenzone, Micronised Titanium Dioxide, Extract of Birch, Mallow and hops.	https://www.amazon.com/Lotus-Herbals-Matte-Daily-Sunblock/dp/B006LXDD0O

Neogen Dermology	Day-Light Protection Sun Screen SPF 50	Water (Aqua), Glycerin, Sodium Lauroyl Isethionate, Stearic Acid, Sodium Stearate, Cocamidopropyl Betaine, Sodium Laureth Sulfate, Tallow Acid or Palmitic Acid, Lauric Acid, Sodium Isethionate, Cetearyl Alcohol, Sodium Chloride, Fragrance (Parfum), DMDM Hydantoin, Sodium Cocoate, Tetrasodium EDTA, PEG- 14M, Iodopropynyl Butylcarbamate, Etidronic Acid, Methylisothiazolinone, Titanium Dioxide (CI 77891).	https://www.sephora.com/product/day-light-protection-sun-screen-spf-50-pa-P410761?skuId=1860592&om_mmc=ppc-GG_1165716902_56760231047_aud-299050440478:pla-421224763097_1860592_257731959569_9018944_c&country_switch=us&lang=en&gclid=CjwKCAjw85zdBRB6EiwAov3RirjGFE-p_Nku8x7dsoJFAi9V2OyW3OWf1WjNuCPSqIApfjv5_26SwhoCIDkQAvD_BwE&gclsrc=aw.ds
Neutrogena	Pure & Free® Baby Sunscreen Stick Broad Spectrum SPF 60+	Active Ingredients: Titanium Dioxide 8%, Zinc Oxide 6.8%<br>Inactive Ingredients: Beeswax, BHT, C12-15 Alkyl Benzoate, Dimethicone, Dipropylene Glycol Dibenzoate, Euphorbia Cerifera (Candelilla) Wax, Octyldodecyl Neopentanoate, Ozokerite, Paraffin, Phenyl Trimethicone, Polyethylene, Polyhydroxystearic Acid, Ppg-15 Stearyl Ether Benzoate, Triethoxycaprylylsilane	https://www.neutrogena.com/sun/sun-baby/sun-baby-sticks/pure-and-free-baby-sunscreen-stick-broad-spectrum-spf-60/6886003.html?cgid=sun-baby-sticks#start=1

Origins	Vitazing SPF 15 Energy Boosting Moisturizer	Ingredients: Avobenzone 3.0% , Octisalate 3.0% , Octocrylene 2.7%Ingredients: Water\Aqua\Eau , Ethyl Macadamiate , Butylene Glycol , Methyl Trimethicone , Hdi/Trimethylol Hexyllactone Crosspolymer , Lauryl Peg-9 Polydimethylsiloxyethyl Dimethicone , Peg-100 Stearate , Butyloctyl Salicylate , Jojoba Esters , Glyceryl Stearate , Barosma Betulina Leaf Oil*, Citrus Paradisi (Grapefruit) Peel Oil*, Eucalyptus Globulus (Eucalyptus) Leaf Oil*, Abies Sibirica (Pine) Oil*, Prunus Amygdalus Amara (Bitter Almond) Kernel Oil*, Mentha Arvensis (Field Mint) Leaf Oil*, Rosa Damascena (Rose) Oil*, Limonene , Panax Ginseng (Ginseng) Root Extract , Cordyceps Sinensis Extract , Garcinia Mangostana Peel Extract , Castanea Sativa (Chestnut) Seed Extract , Hordeum Vulgare (Barley) Extract\Extrait D'Orge , Triticum Vulgare (Wheat) Germ Extract , Laminaria Saccharina Extract , Mangifera Indica (Mango) Seed Butter , Butyrospermum Parkii (Shea Butter) , Caffeine , Citrus Aurantium Amara (Bitter Orange) Flower Water , Anthemis Nobilis (Chamomile) Flower Extract , Potassium Cetyl Phosphate , Glycerin , Cetyl Alcohol , Isopropyl Myristate , Trehalose , Ethylhexylglycerin , Linoleic Acid , Squalane , Polymethyl Methacrylate , Dehydroxanthan Gum , Ascorbyl Tocopheryl Maleate , Glyceryl Acrylate/Acrylic Acid Copolymer , Sodium Hyaluronate , Caprylyl Glycol , Ammonium Acryloyldimethyltaurate/Vp Copolymer , Citric Acid , Stearic Acid , Silica , Sodium Dehydroacetate , Phenoxyethanol , Mica , Iron Oxides (Ci 77491, Ci 77492, Ci 77499) , Titanium Dioxide (Ci 77891) * Essential Oil <ILNILN44030>	https://www.origins.com/product/15352/13004/skincare/moisturize/moisturizers/vitazing/spf-15-energy-boosting-moisturizer#pr-reviewdisplay

Origins	Plantscription SPF 15 Anti-aging Foundation	Ingredients: Octinoxate 3.00% , Titanium Dioxide 1.40%Ingredients: Water\Aqua\Eau , Cyclopentasiloxane , Phenyl Trimethicone , Peg-10 Dimethicone , Butylene Glycol , Dimethicone , Isocetyl Alcohol , Caprylic/Capric Triglyceride , Isododecane , Wheat Germ Glycerides , Magnesium Sulfate , Tribehenin , Anogeissus Leiocarpus Bark Extract , Rosa Damascena Flower Oil*, Lavandula Angustifolia (Lavender) Oil*, Pelargonium Graveolens Flower Oil*, Illicium Verum (Anise) Oil*, Citrus Aurantium Bergamia (Bergamot) Fruit Oil*, Carthamus Tinctorius (Safflower) Seed Oil*, Myristica Fragrans (Nutmeg) Kernel Oil*, Citrus Aurantium Dulcis (Orange) Oil*, Citrus Nobilis (Mandarin Orange) Peel Oil*, Citrus Medica Limonum (Lemon) Peel Oil*, Litsea Cubeba Fruit Oil*, Hibiscus Abelmoschus Extract, Geraniol, Linalool, Citronellol, Limonene , Mimosa Tenuiflora Bark Extract , Salvia Sclarea (Clary) Extract , Scutellaria Baicalensis Root Extract , Sigesbeckia Orientalis (St. Paul'S Wort) Extract , Foeniculum Vulgare (Fennel) Seed Extract , Disteardimonium Hectorite , Hordeum Vulgare (Barley) Extract\Extrait D'Orge , Triticum Vulgare (Wheat) Germ Extract , Castanea Sativa (Chestnut) Seed Extract , Centaurium Erythraea (Centaury) Extract , Anthemis Nobilis (Chamomile) Flower Extract , Simmondsia Chinensis (Jojoba) Seed Oil , Peg/Ppg-18/18 Dimethicone , Glycerin , Squalane , Polysilicone-11 , Ethylhexyl Palmitate , Cholesterol , Linoleic Acid , Ethylhexylglycerin , Methicone , Polymethylsilsesquioxane , Palmitoyl Oligopeptide , Caffeine , Tocopheryl Acetate , Sodium Hyaluronate , Sorbitan Sesquioleate , Sorbitan Stearate , Aminopropyl Ascorbyl Phosphate , Trehalose , Triethyl Citrate , Hydrogenated Lecithin , Propylene Carbonate , Sorbitan	https://www.origins.com/product/15370/22762/makeup/face-makeup/foundation/plantscription-spf-15/anti-aging-foundation

		Isostearate , Laureth-7 , Maltodextrin , Ascorbyl Palmitate , Xanthan Gum , Alumina , Chloroxylenol , Phenoxyethanol , [+/- Titanium Dioxide (Ci 77891) , Iron Oxides (Ci 77491, Ci 77492, Ci 77499)] * Essential Oil <ILNILN38729>	
SkinCeuticals	Physical Fusion UV Defense Tinted Sunscreen SPF 50	Active ingredients: Zinc Oxide 5%, Titanium Dioxide 6%. Inactive ingredients: Water, Dimethicone, Isododecane, C12-15 Alkyl Benzoate, Undecane, Triethylhexanoin, Isohexadecane, Nylon-12, Caprylyl Methicone, Butyloctyl Salicylate, Phenethyl Benzoate, Styrene/Acrylates Copolymer, Silica, Tridecane, Dicaprylyl Carbonate, Dicaprylyl Ether, Talc, Dimethicone/PEG-10/15 Crosspolymer, Aluminum Stearate, Pentylene Glycol, PEG-9 Polydimethylsiloxyethyl Dimethicone, Alumina, Polyhydroxystearic Acid, Phenoxyethanol, Magnesium Sulfate, Caprylyl Glycol, Iron Oxides, PEG-8 Laurate, Disteardimonium Hectorite, Triethoxycaprylylsilane, Tocopherol, Propylene Carbonate, Artemia Extract, Benzoic Acid, PEG-9, Disodium Stearoyl Glutamate, Aluminum Hydroxide.	https://www.lovelyskin.com/o/skinceuticals-physical-fusion-uv-defense-spf-50?ref=gbase&select=12838&gclid=CjwKCAjw85zdBRB6EiwAov3Rit1XTHOdAOLE-wS6s1DfdUJ0uZrms1pQk0CjkD_J_PRHEvXCEEjJURoCwJgQAvD_BwE

Table 6. Deodorant

Brand	Product Name	Ingredients	Website URL
Old Spice	Classic	Active Ingredient - Sodium Fluoride 0.243% - Purpose Anticavity Toothpaste.<br><br>Inactive Ingredients: Sorbitol, Water, Hydrated Silica, Disodium Pyrophosphate, Sodium Hydroxide, Sodium Lauryl Sulfate, Flavor, Xanthan Gum, Sodium Saccharin, Carbomer, Cellulose Gum, Titanium Dioxide.	https://www.pharmapacks.com/products.php?product=Old-Spice-Classic-Deodorant-Stick-Original-Scent-2.25-oz&gclid=EAIaIQobChMIwq2Qx4Xu3AIVg0NpCh1GaAd6EAQYASABEgKVwfD_BwE

Table 7. Shaving Cream

Brand	Product Name	Ingredients	Website URL
Cremo	Lavender Bliss Moisturizing Concentrated Shave Cream	Water,sodium cocoyl isethionate,stearic acid,cetearyl alcohol,glycol distearate,macadamia ternifolia seed oil,fragrance,citrus limon (lemon) fruit extract,carica papaya (papaya) fruit extract,olea europaea (olive) leaf extract,calendula officinalis flower extract,aloe barbadensis leaf juice,lavandula angustifolia (lavender) oil,titanium dioxide,hydroxypropyl guar hydroxypropyltrimonium chloride,citric acid,polyether-1,tocopheryl acetate,phenoxyethanol,ethylhexylglycerin, perfluorodecalin,allantoin,xylitol.	https://www.target.com/p/cremo-153-lavender-bliss-moisturizing-concentrated-shave-cream-6-oz/-/A-51087673?sid=2765S&ref=tgt_adv_XS000000&AFID=google_pla_df&CPNG=PLA_Health+Beauty+Shopping_Local&adgroup=SC_Health+Beauty&LID=700000001170770pgs&network=g&device=c&location=9018948&gclid=CjwKCAjw2_LcBRBYEiwA_XVBU1ER-nRKgfZavpLz9DwwIJu5P-JyxDez5Z4AB6vPAS2wvVUkoFhD1RoC-yQQAvD_BwE&gclsrc=aw.ds
Cremo	Cooling Shave Cream	Water (Aqua), Sodium Cocoyl Isethionate, Stearic Acid, Glycol Distearate, Cetearyl Alcohol, Macadamia Ternifolia Seed Oil, Citrus Medica Limonum (Lemon) Fruit Ex-Tract, Carica Papaya (Papaya) Fruit Extract, Olea Europaea (Olive) Leaf Extract, Calendula Officinalis Flower Extract, Aloe Barbadensis Leaf Juice, Tea Tree Oil, Eucalyptus Globulus Leaf Oil, Peppermint Oil, L-Menthol, Polyether-1,	https://www.target.com/p/cremo-cooling-shave-cream-6-fl-oz/-/A-18782616

		Hydroxypropyl Guar Hydroxypropyltrimonium Chloride, Allantoin, Xylitol, Phenoxyethanol, Ethylhexyl-Glycerin, Titanium Dioxide, Disodium Edta, Citric Acid, 2-Amino-2-Metyl-1-Propanol, Bht, Perflurodecalin.	
Cremo	Coconut Mango Moisturizing Shave Cream	Water, Sodium Cocoyl Isethionate, Stearic Acid, Glycol Distearate, Cetearyl Alcohol, Macadamia Ternifolia Seed Oil, Polyether-1, Hydroxypropyl Guar Hydroxypropyltrimonium Chloride, Allantoin, Aloe Barbadensis Leaf Juice, Calendula Officinalis Flower Extract, Citrus Medica Limonum (Lemon) Fruit Extract, Carica Papaya (Papaya) Fruit Extract, Olea Europaea (Olive) Leaf Extract, Xylitol, Perfluoromethylcyclopentane, Phenoxyethanol, Ethylhexylglycerin, Titanium Dioxide, Citric Acid, Fragrance, Benzyl Benzoate, Benzyl Salicylate, Butylphenyl Methylpropional (Lillal), Coumarin, Limonene.	https://www.target.com/p/cremo-153-coconut-mango-moisturizing-shave-cream-6-oz/-/A-51391068
Cremo	Original Shave Cream	Water, Sodium Cocoyl Isethionate, Stearic Acid, Glycol Distearate, Cetearyl Alcohol, Macadamia Ternifolia Seed Oil, Polyether-1, Hydroxypropyl Guar Hydroxypropyltrimonium Chloride, Allantoin, Aloe Barbedensis Leaf Juice, Calendula Officinalis Flower extract, Xylitol, Citrus Medica Limonum (Lemon) Fruit and Carica Papaya Fruit Extract, Olea Europaea (Olive) Leaf Extract, Linalool, Limonene, BHT, Perfluoromethylcyclopentane, Phenoxyethanol, Ethylhexylglycerin, Titanium Dioxide, Citric Acid, Fragrance (Parfum)	https://cremocompany.com/mens-shave/mens-original-shave-cream
Dove	Men+Care Hydrate+Pro-Moisture Shave Cream	Water (Aqua), Glycerin, Sodium Lauroyl Isethionate, Stearic Acid, Sodium Stearate, Cocamidopropyl Betaine, Sodium Laureth Sulfate, Tallow Acid or Palmitic Acid, Lauric Acid, Sodium Isethionate, Cetearyl Alcohol, Sodium Chloride, Fragrance (Parfum), DMDM Hydantoin, Sodium Cocoate, Tetrasodium EDTA, PEG- 14M,	https://www.dove.com/us/en/men-care/shaving/men-care-hydrate-pro-moisture-shave-cream.html

		Iodopropynyl Butylcarbamate, Etidronic Acid, Methylisothiazolinone, Titanium Dioxide (CI 77891).	
eos	Shave Cream Pomegranate Raspberry	Aqua, Cetyl Alcohol, Propylene Glycol, Cocoglycerides, Stearyl Alcohol, Glycerin, Laureth-23, Titanium Dioxide (CI 77891), Colloidal Oatmeal, Petrolatum, Glyceryl Stearate SE, Butyrospermum Pakii (Shea) Butter, Aloe Barbadensis Leaf Juice, Ascorbyl Palmitate, Tocopheryl Acetate, Fragrance (Parfum), Magnesium Aluminum Silicate, Dimethicone, Carbomer, Disodium EDTA, Phenoxyethanol, Caprylyl Glycol, Hexylene Glycol, Potassium Sorbate, Sodium Benzoate, Sodium Hydroxide, Citric Acid, Yellow 6 (CI 15985), Red 40 (CI 16035)	https://www.walmart.com/ip/eos-Shave-Cream-Pomegranate-Raspberry-2-5-FL-OZ/55456041
eos	Ultra Moisturizing Shave Cream Vanilla Bliss	Water (Aqua), Cetyl Alcohol, Propylene Glycol, Cocoglycerides, Stearyl Alcohol, Glycerin, Laureth-23, CI77891 (Titanium Dioxide), Colloidal Oatmeal, Petrolatum, Glyceryl Stearate SE, Butyrospermum Parkii (Shea) Butter, Aloe Barbadensis Leaf Juice, Ascorbyl Palmitate, Tocopheryl Acetate, Fragrance (Parfum), Magnesium Aluminum Silicate, Dimethicone, Carbomer, Disodium EDTA, Phenoxyethanol, Caprylyl Glycol, Hexylene Glycol, Potassium Sorbate, Sodium Benzoate, Sodium Hydroxide, Citric Acid, CI 15985 (Yellow 6), CI 16035 (Red 40), CI 42090 (Blue 1), CI 19140 (Yellow 5), Limolene, Linalool.	https://evolutionofsmooth.com/shave-cream/ultra-moisturizing-shave-cream.html
Skintimate	Skin Therapy Lotionized Shave Gel for Dry Skin	Water, Palmitic Acid, Triethanolamine, Sunflower Seed Oil Glyceride, Isopentane, Sorbitol, Stearic Acid, Isobutane, Lanolin Alcohol, PVP, Tocopheryl Acetate, PEG-90m, Hydrogenated Vegetable Oil, Olea Europaea (Olive) Fruit Oil, Fragrance, Titanium Dioxide, Red 40.	https://www.amazon.com/Skintimate-Therapy-Lotionized-Shave-Women/dp/B000URD53C?th=1

Up & Up	Women's Shave Gel for Dry Skin Relief	Water, Palmitic Acid, Triethanolamine, Isopentane, Sunflower Seed Oil Glyceride, Glycerin, Propylene Glycol, Stearic Acid, Sorbitol, Isobutane, Acrylic Acid/VP Crosspolymer, Lanolin Alcohol, Fragrance, Tocopheryl Acetate, Hydroxypropyl Methylcellulose, PEG-90M, Hydrogenated Olive Oil, Olea Europaea (Olive) Fruit Oil, Hydroxyethylcellulose, Titanium Dioxide, Red 40.	https://www.target.com/p/women-s-shave-gel-for-dry-skin-relief-7oz-up-up-153-compare-to-skintimate-dry-skin/-/A-14667360

Table 8. Soap/Bodywash

Brand	Product Name	Ingredients	Website URL
Ambi	Skincare Bars Complexion Cleansing Bar	Sodium Tallowate, Sodium Cocoate and/or Sodium Palm Kernelate, Fragrance, Water, Titanium Dioxide, Diethylhexyl Sodium Sulfosuccinate, Sodium Chloride, Pentasodium Pentetate, Simethicone, Xanthan Gum, Glutaral, Tetrasodium Etidronate, Chromium Hydroxide Green (CI 77289), Iron Oxides	https://www.amazon.com/gp/product/B0056GIKI2/ref=s9_acsd_hps_bw_c_x_7_w
Caress	Daily Silk Body Wash	Water/Agua, Sodium Laureth Sulfate, Sodium Chloride, Acrylates Copolymer, Cocamide Mea, Fragrance/Fragancia, Tetrasodium Edta, Propylene Glycol, Glycine Soja (Soybean) Oil, Helianthus Annuus (Sunflower) Seed Oil, Glycerin, Hydrolyzed Silk, Ppg-9, Methylchloroisothiazolinone, Prunus Persica (Peach) Fruit Extract, Methylisothiazolinone, Citrus Aurantium Dulcis (Orange) Flower Oil, Mica (Ci 77019), Titanium Dioxide (Ci 77891), Yellow 5 (Ci 19140), Red 33 (Ci 17200).	https://www.target.com/p/caress-daily-silk-body-wash-18oz/-/A-11223405
Caress	Exfoliating Body Wash, Evenly Gorgeous	Water/Agua Sodium Laureth Sulfate Hydrated Silica Sodium Chloride Acrylates Copolymer Cocamide MEA Fragrance/Fragancia Propylene Glycol Glycerin Mica (CI 77019) Citric Acid Helianthus Annuus (Sunflower) Seed Oil Tetrasodium EDTA Glycine Soja (Soybean) Oil Gelatin Acacia Senegal Gum PPG-9 Butyrospermum Parkii (Shea) Butter Methylchloroisothiazolinone Caramel Molasses Maltodextrin Methylisothiazolinone Iron Oxides (CI 77491) Titanium Dioxide (CI 77891) Yellow 6 (CI 15985) Red 33 (CI 17200) Blue 1 (CI 42090)	https://www.amazon.com/Caress-Exfoliating-Body-Evenly-Gorgeous/dp/B00ULKRA6G

Clean & Clear	Morning Burst Facial Cleanser	Water Sodium Laureth Sulfate Glycerin Acrylates Copolymer Cocamidopropyl Betaine Fragrance Phenoxyethanol Cellulose Menthyl Lactate Manntiol Disodium EDTA Methylparaben Menthol Propylparaben Ethylparaben Tocopheryl Acetate Sodium Ascorbyl Phosphate Retinyl Palmitate Hydroxypropyl Methylcellulose Acrylates/Ammonium Methacrylate Copolymer Talc Triethyl Citrate Cucumis Sativus (Cucumber) Fruit Extract Citrus Grandis (Grapefruit) Fruit Extract Panax Ginseng Root Extract Zingiber Officinale (Ginger) Root Extract Iron Oxides Titanium Dioxide Mica, May also contain Sodium Hydroxide	https://www.cleanandclear.com/facial-cleansers/morning-burst-facial-cleanser?utm_source=google&utm_medium=cpc&utm_campaign=GO-USA-ENG-PS-Clean%26Clear-BP-BM-RN-Cleansers&utm_content=Brand-Cleansers-Morning+Burst-Facial+Cleanser&utm_term=%2Bclean+%2B%26+%2Bclear+%2Bmorning+%2Bburst+%2Bface+%2Bwash&&gclid=Cj0KCQiAnNXiBRCoARIsAJe_1cr9spOJEY1NSfOcH9wUX7s6xHhKj_osoHlr-ZUi00fkXeQHB8J49yAaApm_EALw_wcB&gclsrc=aw.ds
Dove	Sensitive Skin Beauty Bar	Sodium Lauroyl Isethionate, Stearic Acid, Sodium Tallowate Or Sodium Palmitate, Lauric Acid, Sodium Isethionate, Water, Sodium Stearate, Cocamidopropyl Betaine, Sodium Cocoate Or Sodium Palm Kernelate, Sodium Chloride, Tetrasodium Edta, Tetrasodium Etidronate, Maltol, Titanium Dioxide (Ci 77891)	https://www.dove.com/us/en/washing-and-bathing/beauty-bar/sensitive-skin-beauty-bar.html

Dove	White Beauty Bar	Sodium Lauroyl Isethionate, Stearic Acid, Sodium Tallowate Or Sodium Palmitate, Lauric Acid, Sodium Isethionate, Water, Sodium Stearate, Cocamidopropyl Betaine, Sodium Cocoate Or Sodium Palm Kernelate, Fragrance, Sodium Chloride, Tetrasodium Edta, Tetrasodium Etidronate, Titanium Dioxide (Ci 77891)	https://www.dove.com/us/en/washing-and-bathing/beauty-bar/white-beauty-bar.html
Dove	Pink Beauty Bar	Sodium Lauroyl Isethionate Stearic Acid , Sodium Tallowate and/or Sodium Palmate, Sodium Isethionate, Lauric Acid, Water, Sodium Stearate, Cocamidopropyl Betaine, Sodium Cocoate or Sodium Palm Kernelate, Fragrance, Sodium Chloride, Propylene Glycol, Tetrasodium EDTA, Tetrasodium Etidronate, Red 4, Red 33, Titanium Dioxide	https://www.dove.com/us/en/washing-and-bathing/beauty-bar/pink-beauty-bar.html
Dove	Men+Care Body+Face Bar	Sodium Lauroyl Isethionate, Stearic Acid, Sodium Tallowate Or Sodium Palmitate, Sodium Stearate, Water (Aqua), Sodium Isethionate, Lauric Acid, Sodium C14-16 Olefin Sulfonate, Sodium Cocoate Or Sodium Palm Kernelate, Fragrance (Parfum), Dipropylene Glycol, Sodium Chloride, Propylene Glycol, Tetrasodium EDTA, Tetrasodium Etidronate, Green 5 (CI 61570), Orange 4 (CI 15510), Titanium Dioxide (CI 77891)	https://www.boxed.com/product/4941/dove-mencare-bodyface-bar-10-x-4-oz.-extra-fresh?utm_source=google&utm_medium=cpc&utm_campaign=dsa-low&utm_purpose=all&utm_experiment=none&utm_ad_id=321073152935&utm_term=&gclid=Cj0KCQiAnNXiBRCoARIsAJe_1co5aoGPWrXePTW0zgSJvpQ8t4R2A6FxC0GToNPiXVFjZbcbOkr4uT8aApwvEALw_wcB

Equate	Beauty AM Refreshing Facial Cleanser	Water, Sodium Cocoyl Sarcosinate, Sodium Laureth Sulfate, Acrylates Copolymer, Cocamidopropyl Betaine, Glycerin, Polysorbate 20, Phenoxyethanol, Methylparaben, Ethylparaben, Butylparaben, Propylparaben, Isobutylparaben, Fragrance, Sodium Hydroxide, Metnhol, Methyl Lactate, Disodium Edta, Panax Ginseng Root Extract, Zingiber Officinal (Ginger) Root Extract, Mannitol, Cellulose, Hydroxypropyl Methylcellulose, Tocopheryl Acetate, Retinyl Palmitate, Titanium Dioxide, Iron Oxides, Triethyl Citrate, Mica, Acrylates/Ammonium Methacrylate Copolymer, Talc, Cucumis Sativus (Cucumber) Fruit Extract, Citrus Grandis (Grapefruit) Fruit Extract, Sodium Ascorbyl Phosphate.	https://www.walmart.com/ip/Equate-Beauty-AM-Refreshing-Facial-Cleanser-8-Oz/21312122
e·ra organics	Tea Tree Oil Face Cleanser	Organic Aloe Barbadensis Gel (Aloe Vera), Cocamidopropyl Betaine (Coconut Oil), Propanediol, Hydroxyethyl Cellulose, Melaleuca Alternifolia (Tea Tree) Oil, Salicylic Acid, Xanthan Gum, Zinc Oxide, Mica, Titanium Oxide, Organic St. John's Wort, Organic Pomegranate Seed Oil, Organic Burdock, Organic Dandelion Root, Organic Goldenseal, Phenoxyethanol	https://www.amazon.com/Tea-Tree-Oil-Face-Cleanser/dp/B073X4GYRV/ref=sr_1_1_a_it?ie=UTF8&qid=1549128779&sr=8-1-spons&keywords=trader+joe%27s+tea+tree+face+wash&psc=1
Korres	Greek Yoghurt Foaming Cream Cleanser	Aqua/Water/Eau, Sodium Cocoyl Isethionate, Coco-Betaine, Cetearyl Alcohol, Disodium Lauryl Sulfosuccinate, Zea Mays (Corn) Starch, Glycerin, Cocamidopropyl Betaine, Sodium Methyl Cocoyl Taurate, Magnesium Aluminum Silicate, Glyceryl Laurate, Sclerotium Gum, Amaranthus Caudatus Seed Extract, Fragrance (Parfum), Hydrogenated Castor Oil, Hydrolyzed Rice Protein, Lactose, Lonicera Caprifolium (Honeysuckle) Flower Extract,Lonicera Japonica (Honeysuckle) Flower Extract, Sodium Cocoyl Alaninate, Sodium Cocoyl	https://www.skincarerx.com/korres-greek-yoghurt-foaming-cream-cleanser/11288471.html

		Hydrolyzed Amaranth Protein, Sodium Phytate, Titanium Dioxide, Whey Protein, Yogurt Powder.	
Olay	Olay Moisture Outlast Sensitive Beauty Bar	Sodium Lauroyl Isethionate, Paraffin, Sodium Cocoglyceryl Ether Sulfonate, Glycerin, Water/Eau, Talc, Magnesium Stearate, Stearic Acid, Sodium Isethionate, Magnesium Cocoate, Sodium Stearate, Coconut Acid, Sodium Chloride, Sodium Cocoate, Magnesium Laurate, Lauric Acid, Titanium Dioxide, Sodium Laurate, Fragrance/Parfum, Tetrasodium Edta, Etidronic Acid, PEG-90M	https://www.olay.com/en-us/skin-care-products/sensitive-beauty-bar
Old Spice	Fresh Collection Fiji Scent Bar Soap	Sodium Tallowate And/or Et/Ou Sodium Palmate, Sodium Palm Kernelate And/or Et/Ou Sodium Cocoate, Water/Eau, Fragrance/Parfum, Sodium Chloride, Glycerin, Titanium Dioxide, Coconut Acid*, Tallow Acid*, Palm Acid*, Palm Kernel Acid*, Citric Acid*, Sodium Citrate*, Tetrasodium EDTA *Contains One Or More Of These Ingredients/Contient Un Ou Plusieurs De Ces Ingredients	https://www.amazon.com/Old-Spice-Fresh-Collection-Scent/dp/B00AFCOUVI/ref=sr_1_3_a_it?ie=UTF8&qid=1549127082&sr=8-3&keywords=old+spice+soap+bars
Shea Moisture	Indian Hemp & Shea Butter Inflammation Therapy Bar Soap	Active Ingredients: Calendula Officinalis Flower Extract 1X (Calendula) Inactive Ingredients: Sodium Palmate, Sodium Palm Kernalate, Water (Aqua), Glycerin, Fragrance (Parfum) Coconut Acid, Palmific Acid, Sodium Chloride, Butyrospermum Parkii (Shea Butter)*, Titanium Dioxide (CI 77891), Aspalathus Linearis Leaf, Cannabis Sativa Seed Oil, Yellow 10 (CI 47005), Tetrasodium EDTA, Tetrasodium Etidronate, Iron Oxides, (CI 77499), Nelia Azadirachta Seed OilRed 4 (CI 14700), Green 3 (CI 42053).	https://www.sheamoisture.com/indian-hemp-shea-butter-inflammation-therapy-bar-soap.html

St. Ives	Fresh Skin Apricot Scrub	Water (Aqua, Eau), Juglans Regia (Walnut) Shell Powder, Glyceryl Stearate, Glycerin, Sodium Lauryl Sulfoacetate, Zea Mays (Corn) Kernel Meal, Cocamidopropyl Betaine, Cetearyl Alcohol, Cetyl Alcohol, PEG-100 Stearate, Cetyl Acetate, Titanium Dioxide (CI 77891), Polysorbate 60, Ceteareth-20, Acetylated Lanolin Alcohol, Triethanolamine, Carbomer, Fragrance (Parfum), PPG-2 Methyl Ether, Phenethyl Alcohol, Limonene, Linalool, Methylisothiazolinone, Prunus Armeniaca (Apricot) Fruit Extract.	https://www.stives.com/products/fresh-skin-apricot-scrub?gclid=Cj0KCQiAnNXiBRCoARIsAJe_1cpIJPF4LS4kPwvNKsTg-uyqlP37Q711G-b_8mlhoR-psm0IpIT8pNkaAhe6EALw_wcB&gclsrc=aw.ds
Suave	Skin Solutions Body Lotion, Smoothing with Cocoa Butter & Shea	Water; Glycerin; Stearic Acid; Mineral Oil; Glycol Stearate; Theobroma Cacao (Cocoa) Butter; Butyrospermum Parkii (Shea Butter); Glyceryl Stearate; Cetyl Alcohol; Petrolatum; Fragrance; Dimethicone; Stearamide AMP; Magnesium Aluminum Silicate; Triethanolamine; Carbomer; Propylene Glycol; Methylparaben; Tetrasodium EDTA; DMDM Hydantoin; Caramel; Titanium Dioxide (Cl 77891); Yellow 5 (Cl 19140); Yellow 6 (Cl 15985)	https://www.walmart.com/ip/2-Pack-Suave-Skin-Solutions-Body-Lotion-Smoothing-with-Cocoa-Butter-Shea-18-0-oz/903962868
Up & Up	Apricot Blemish Scrub	Water, Juglans Regia (Walnut) Shell Powder, Glyceryl Stearate Se, Propylene Glycol, Sodium Laureth Sulfate, Zea Mays (Corn) Kernel Meal, Cocamidopropyl Betaine, Cetearyl Alcohol, Triethanolamine, Cetyl Alcohol, Peg-100 Stearate, Glyceryl Stearate, Cetyl Acetate, Ceteareth-20, Polysorbate 60, Carbomer, Acetylated Lanolin Alcohol, Fragrance (Parfum), Phenethyl Alcohol, Ppg-2-Methyl Ether, Limonene, Linalool, Methylisothiazolinone, Glycerin, Prunus Armeniaca (Apricot) Fruit Extract, Titanium Dioxide (Ci 1778911)	https://www.target.com/p/apricot-blemish-scrub-6oz-up-up-153-compare-to-st-ives-apricot-scrub/-/A-11574171

Zest Soap Bar	Moisture Rich Soap Bar	Ingredients: Sodium Tallowate And/Or Sodium Palmate, Sodium Cocoate And/Or Sodium Palm Kernelate, Water, Glycerin, Coconut Acid*, Palm Acid*, Palm Kernel Acid*, Tallow Acid*, Fragrance, Sodium Chloride, Titanium Dioxide, Pentasodium Pentetate, Tetrasodium Etidronate, Propylene Glycol, Butyrospermum Parkii (Shea) Butter, Theobroma Cacao (Cocoa) Seed Butter, Yellow 5, Red, 5, Blue 1. *Contains One Or More of These Ingredients.	https://www.walmart.com/ip/High-Ridge-Brands-Zest-Moisture-Rich-Bars-8-ea/34788363

Table 9. Others

Brand	Product Name	Image	Website URL
Aveeno	Positively Radiant Daily Moisturizer	Active Ingredients: Avobenzone 3%, octinoxate 7.5%, octisalate 2%. Inactive Ingredients: Water, C12-15 alkyl benzoate, cetearyl alcohol, dimethicone, glycine soja (soybean) seed extract, glycerin, bis-phenylpropyl dimethicone, arachidyl alcohol, phenoxyethanol, cetearyl glucoside, panthenol, benzyl alcohol, ethylene/acrylic acid copolymer, behenyl alcohol, steareth-2, fragrance, steareth-21, polyacrylamide, polymethyl methacrylate, arachidyl glucoside, disodium EDTA, methylparaben, C13-14 isoparaffin, ethylparaben, butylparaben, laureth-7, benzalkonium chloride, propylparaben, isobutylparaben, iodopropynyl butylcarbamate, BHT, titanium dioxide, mica, silica.	https://www.amazon.com/Aveeno-Positively-Moisturizer-Spectrum-Sunscreen/dp/B001IM5VT4
Bare Minerals	Complexion Rescue Tinted Moisturizer	ACTIVE INGREDIENTS: Titanium dioxide 6.2% INACTIVE INGREDIENTS: water, coconut alkanes, propanediol, squalane, trehalose, isostearic acid, glycerin, silica, agar, caprylic/capric triglyceride, globularia cordifolia callus culture extract, salicornia herbacea extract, melilotus officinalis extract, coco-caprylate/caprate, butylene glycol, lauroyl lysine, sodium hyaluronate, succinoglycan, polysorbate 60, cellulose gum, polyglyceryl-4 laurate/succinate, sorbitan sesquiisostearate, magnesium stearate, magnesium hydroxide, magnesium chloride, potassium chloride, calcium chloride, potassium sorbate, phenoxyethanol. may contain: titanium dioxide, iron oxides.	https://www.bareminerals.com/makeup/face/foundation/complexion-rescue-tinted-hydrating-gel-cream-broad-spectrum-spf-30/USmastercomplexionrescue.html

BeautyCounter	Tint Skin Foundation (Sand)	Ingredients: Aqua/Water/Eau, Coco-Caprylate, Isododecane, Trimethylsiloxysilicate, Caprylic/Capric Triglyceride, Dimethicone/Vinyl Dimethicone Crosspolymer, Isostearic Acid, Polyglyceryl-4 Diisostearate/Polyhydroxystearate/Sebacate, Isoeicosane, Propanediol, Jojoba Esters, Glycerin, Magnesium Sulfate, Cera Alba/Beeswax/Cire d'abeille, Dimethicone, Gluconolactone, Sodium Hyaluronate, Sodium Chloride, Sodium Benzoate, Hydrogenated Castor Oil, Polyhydroxystearic Acid, Lecithin, Ethylhexyl Palmitate, Isopropyl Myristate, Polyglyceryl-3 Polyricinoleate, Prunus Amygdalus Dulcis (Sweet Almond) Oil, Citrus Aurantium Bergamia (Bergamot) Fruit Oil, Citrus Aurantium Dulcis (Orange) Peel Oil, Rosa Damascena (Rose) Flower Oil, Salvia Sclarea (Clary) Oil, Citrus Aurantium Dulcis (Orange) Flower Oil, Vanilla Planifolia (Vanilla) Fruit , Triethoxycaprylylsilane, Titanium Dioxide (CI 77891), Iron Oxides (CI 77491, CI 77492, CI 77499)	https://www.beautycounter.com/product/tint-skin-complexion-coverage
Bliss	Ex-glow-sion Facial Moisturizer	Water (Aqua) (Eau), Cyclopentasiloxane, Caprylic/Capric Triglyceride, Glycerin, Butyrospermum Parkii (Shea) Butter, Cetearyl Alcohol, Squalane, Xylitylglucoside, Hydroxyethyl Acrylate/Sodium Acryloyldimethyl Taurate Copolymer, Mica, Anhydroxylitol, Cetearyl Glucoside, Caprylyl Methicone, Propanediol, Panthenol, Tocopherol, Tetrahexyldecyl Ascorbate, Pyrus Communis (Pear) Fruit Extract, Cucumis Sativus (Cucumber) Fruit Extract, Lavandula Angustifolia (Lavender) Flower Extract, Rosmarinus Officinalis (Rosemary) Leaf Extract, Camellia Sinensis Leaf Extract, Helianthus Annus (Sunflower) Seed Oil, Glycine Soja (Soybean) Oil, Lavandula Angustifolia (Lavender) Oil, Sodium Phytate, Dimethicone/Vinyl Dimethicone	https://www.blissworld.com/ex-glow-sion/

		Crosspolymer, Xylitol, Polysorbate 60, Glyceryl Stearate, Sorbitan Isostearate, Cetyl Alcohol, Stearyl Alcohol, Cetearyl Olivate, Sorbitan Olivate, PEG-100 Stearate, Polyglyceryl-4 Laurate Succinate, Triethoxycaprylylsilane, Isohexadecane, Phenoxyethanol, Ethylhexylglycerin, Citric Acid, Linalool, Titanium Dioxide (CI 77891)	
Blk Opl	True Color Pore Perfecting Liquid Foundation	Water (Aqua), Butylene Glycol, Glycerin, Propylene Glycol Stearate, PEG-8, Oleic Acid, Cetyl Esters, Kaolin, Lecithin, Magnesium Aluminum Silicate, Tocopherol, Ascorbyl Palmitate, Simethicone, Panthenol, Glyceryl Laurate, Cetyl Alcohol, Cellulose Gum, Stearyl Alcohol, Polypropylene, Polymethyl Methacrylate, Tromethamine, Polysorbate 20, Sodium Dehydroacetate, Hexylene Glycol, Caprylyl Glycol, Phenoxyethanol, Disodium EDTA. May Contain: Mica, Titanium Dioxide (CI 77891), Iron Oxides (CI 77491, CI 77492, CI 77499), Ultramarines (CI 77007), Chromium Hydroxide Green (CI 77289), Chromium Oxide Greens (CI 77288).	https://blackopalbeauty.com/products/true-color-pore-perfecting-liquid-foundation

e.l.f	Perfect Blend Concealer	Apricot Beige: Water (Aqua), Glycerin, Paraffinum Liquidum (Mineral Oil), Isononyl Isononanoate, Dimethicone, Cyclopentasiloxane, Cetyl PEG/PPG-10/1 Dimethicone, Cyclohexasiloxane, PEG/PPG-15/15 Dimethicone, Butylene Glycol Dicaprylate/Dicaprate, Sorbitan Sesquioleate, Magnesium Sulfate, Phenoxyethanol, Polyglyceryl-3 Diisostearate, Disteardimonium Hectorite, Fragrance (Parfum), Triethoxycaprylylsilane, Lecithin, Polyhydroxystearic Acid, Ethylhexylglycerin, Disodium EDTA, Isopropyl Myristate, Ethylhexyl Palmitate, Isostearic Acid, Polyglyceryl-3 Polyricinoleate, Xanthan Gum May Contain: Titanium Dioxide (CI 77891), Iron Oxides (CI 77491, CI 77492, CI 77499) - Light Beige: Water (Aqua), Glycerin, Paraffinum Liquidum (Mineral Oil), Isononyl Isononanoate, Dimethicone, Cyclopentasiloxane, Cetyl PEG/PPG-10/1 Dimethicone, Cyclohexasiloxane, PEG/PPG-15/15 Dimethicone, Butylene Glycol Dicaprylate/Dicaprate, Sorbitan Sesquioleate, Magnesium Sulfate, Phenoxyethanol, Polyglyceryl-3 Diisostearate, Disteardimonium Hectorite, Fragrance (Parfum), Triethoxycaprylylsilane, Lecithin, Polyhydroxystearic Acid, Ethylhexylglycerin, Disodium EDTA, Isopropyl Myristate, Ethylhexyl Palmitate, Isostearic Acid, Polyglyceryl-3 Polyricinoleate, Xanthan Gum May Contain: Titanium Dioxide (CI 77891), Iron Oxides (CI 77491, CI 77492, CI 77499)	https://www.elfcosmetics.com/perfect-blend-concealer/200119.html

EVER	Daylight Tinted Moisturizer	Active Ingredients: Titanium Dioxide 4.5%. Zinc Oxide 6%, Inactive Ingredients: Water, Cyclopentasiloxane, C12-15 Alkyl Benzoate, Dimethicone, Polyglyceryl-3 Polydimethylsiloxyethyl Dimethicone, Dimethicone/Peg-10/15 Crosspolymer Stearic Acid, Aluminum Hydroxide, Sodium Chloride, Glycerin, Magnolia Officinalis Bark Extract, Ectoin, Ergothioneine, Sodium Hyaluronate, Vitis Vinifera (Grape) Seed Extract, Citrus Limon (Lemon) Peel Oil, Citrus Aurantium Dulcis (Orange) Peel Oil, Citrus Aurantium Bergamia (Bergamot) Fruit Oil, Pelargonium Graveolens Flower Oil, Cymbopogon Martini Oil, Citrus Aurantifolia (Lime) Oil, Juniperus Mexicana Oil, Citral, Anisaldehyde, Tagetes Minuta Flower Oil, Michelia Campaca Flower Oil, Tocopheryl Acetate, Tocopherol, Alcohol, Phenethyl Alcohol, Benzyl Acetate, Hexyl Acetate, Hexenyl Acetate, Gamma-Undecalactone, Allyl Caproate, Caprylyl Glycol, Teprenone, Tetrahexyldecyl Ascorbate, Maltodextrin, Lecithin, Ethylhexyl Palmitate, Caprylic/Capric Triglyceride, Peg/Ppg-18/18 Dimethicone, Polyglycerin-3 Crosspolymer, Hexyl Laurate, Cyclohexasiloxane, Triethoxysilylethyl Polydimethylsiloxyethyl Hexyl Dimethicone, Dimethicone/Vinyl Dimethicone Crosspolymer, Polyglyceryl-4 Isosterate, Cetyl Peg/Ppg-10/1 Dimethicone, Silica Dimethyl Silylate, Triethoxycaprylylsilane, Phenoxyehtanol, Ethylhexylglycerin, Butylene Glycol, Hexylene Glycol, Mica, Silica May Contain: Iron Oxides (Ci77491, Ci77492, Ci77499), Titanium Dioxide (Ci77891)	https://www.everskin.com/products/377-daylight?style=753

Freeman	Avocado and Oatmeal Clay Mask	Water/Aqua/Eau, Kaolin, Bentonite, Titanium Dioxide, Propylene Glycol, Magnesium Aluminum Silicate, Colloidal Oatmeal, Tocopherol, Persea Gratissima (Avocado) Oil, Caprylic/Capric Triglyceride, Montmorillonite, Disodium Edta, Sodium Polyacrylate (Veegum Ultra), Diazolidinyl Urea, Methylparaben, Propylparaben, Methylchloroisothiazolinone, Methylisothiazolinone, Fragrance (Parfum), Yellow 5 (CI 19140), Blue 1 (CI 42090).	https://www.ulta.com/avocado-oatmeal-facial-clay-mask?productId=27872
Freeman	Peeloff Clay Mask	Polyvinyl Alcohol, SD Alcohol 40-B, Peg -8, Propylene Glycol, Glycerin, Silica Dimethyl Silylate, Bentonite, Chamomilla Recutita (Matricaria) Flower Oil, Glycyrrhiza Glabra (Licorice) Root Extract, Camellia Sinensis Leaf Extract, Rosa Canina Fruit Extract, Citrus Medica Limonum (Lemon) Extract, Melaleuca Alternifolia (Tea Tree) Leaf Oil, Azadirachta Indica (Neem) Seed Oil, Salix Alba (Willow) Bark Extract, Musa Sapientum (Banana) Fruit Extract, Cocos Nucifera (Coconut)Extract, Zingiber Officinale (Ginger) Root Extract, Psidium Guajava Fruit Extract, Cymbopogon Schoenanthus Extract, Carica Papaya (Papaya) Fruit Extract, Maranta Arundinacea Root Extract, Rubus Idaeus (Raspberry) Fruit Extract, Santalum Album (Sandalwood) Extract, Macrocystis Pyrifera Extract, Nasturtium Officinale Extract, Aleurites Moluccana Extract, Titanium Dioxide, Polysorbate 20, Methylchloroisothiazolinone, Methylisothiazolinone, Fragrance(Parfum), Iron Oxides, PEG-12 Dimethicone.	https://www.ulta.com/sweet-tea-lemon-peel-away-clay-mask?productId=xlsImpprod12041935

Garnier	5 in 1 Skin Perfector BB cream	Active Ingredients: Octinoxate 3%, Titanium Dioxide 4.7%. Inactive Ingredients: Water, Isononyl Isononanoate, Isohexadecane, Glycerin, Alcohol Denat., Peg-20 Methyl Glucose Sesquistearate, Methyl Glucose Sesquistearate, Cetyl Palmitate, Nylon-12, Cyclohexasiloxane, Propylene Glycol, Hydrogenated Polyisobutene, Stearyl Alcohol, Magnesium Aluminum Silicate, Phenoxyethanol, Caprylyl Glycol, Lithium Magnesium Sodium Silicate, Disodium EDTA, Linalool, Benzyl Salicylate, Limonene, Caffeine, Ascorbyl Glucoside, Benzyl Alcohol, Geraniol, Cellulose Acetate Butyrate, Polyphosphorylcholine Glycol Acrylate, Citral, Ammonium Polyacryloyldimethyl Taurate, Polyvinyl Alcohol, Sodium Chloride, Butylene Glycol, Sodium Hyaluronate, Fragrance; May Contain: Titanium Dioxide, Iron Oxides.	https://www.garnierusa.com/about-our-brands/skinactive/bb-cream/5-in-1-miracle-skin-perfector-bb-cream-oil-free-medium-deep
Mario Badescu	Drying Lotion	Isopropyl Alcohol, Aqua (Water, Eau), Calamine, Zinc Oxide, Titanium Dioxide, Sulfur, Camphor, Talc, Glycerin, Salicylic Acid,	https://www.mariobadescu.com/product/drying-lotion#?tab=ingredients
Maybelline	Dream Fresh BB Cream	TITANIUM DIOXIDE, WATER, ETHYLHEXYL PALMITATE, GLYCERIN, OCTYLDODECYL XYLOSIDE, PHENOXYETHANOL, SODIUM ACRYLATE/SODIUM ACRYLOYLDIMETHYL, TAURATE COPOLYMER, HYDROGENATED LECITHIN, ISOHEXADECANE, HYDROXYETHYL, ACRYLATE/SODIUM ACRYLOYLDIMETHYL TAURATE, COPOLYMER, PEG-30 DIPOLYHYDROXYSTEARATE, SODIUM DEHYDROACETATE, PENTAERYTHRITYL, TETRA-DI-T-BUTYL HYDROXYHYDROCINNAMATE, CAPRYLYL GLYCOL, DISODIUM EDTA, CITRIC ACID, POLYSORBATE	https://www.maybelline.com/face-makeup/bb-cream/dream-fresh-bb-cream

		80, POTASSIUM SORBATE, PROPYLENE GLYCOL, CHAMOMILLA RECUTITA, (MATRICARIA) FLOWER EXTRACT, ALOE, BARBADENSIS LEAF EXTRACT; MAY CONTAIN: IRON OXIDES, TITANIUM DIOXIDE	
Maybelline	Great Lash Mascara	Water/Aqua|Beeswax/Cera Alba|Ozokerite|Shellac|Glyceryl Stearate|Triethanolamine|Propylene Glycol|Stearic Acid|Sorbitan Sesquioleate|Methylparaben|Quaternium-15|Quaternium-22|Simethicone|Butylparaben|May Contain: Iron Oxides (CI 77491, CI 77492, CI 77499)|Titanium Dioxide (CI 77891)|Ultramarines (CI 77007)|Mica	https://www.maybelline.com/eye-makeup/mascara/great-lash-washable-mascara
No 7	Stay Perfect Foundation with spf	OCTINOXATE 5%, TITANIUM DIOXIDE (1.62%), Aqua (Water), Caprylyl Methicone, Cyclopentasiloxane, Peg-9 Dimethicone, Boron Nitride, Talc, Mica, Butylene Glycol, Bis-Isobutyl Peg/Ppg-10/7 Dimethicone Copolymer, Disteardimonium Hectorite, Trimethylsiloxysilicate, Magnesium Sulfate, Phenoxyethanol, Polypropylsilsesquioxane, Propylene Carbonate, Panthenol, Methylparaben, Tocopheryl Acetate, Aluminum Hydroxide, Stearic Acid, Ethylparaben, Triethoxycaprylylsilane, Peg/Ppg Dimethylallylether, Sodium Hyaluronate, Tetrasodium Edta, Isopropyl Palmitate, Lecithin, Tocopherol, Bht, [+/- (May Contain) Ci 77891 (Titanium Dioxide), Ci 77492 (Iron Oxides), Ci 77491 (Iron Oxides), Ci 77499 (Iron Oxides)].	https://www.target.com/p/no7-174-stay-perfect-foundation-spf-15-fair-shades-1oz/-/A-53160706

Organic Wear	Tinted Moisturizer	ACTIVE INGREDIENT: TITANIUM DIOXIDE. INACTIVE INGREDIENTS: CITRUS AURANTIUM DULCIS (ORANGE) FRUIT WATER*, ALCOHOL SD (DENATURED WITH CERTIFIED ORGANIC LAVENDER OIL)*, SIMMONDSIA CHINENSIS (JOJOBA) SEED OIL*, HELIANTHUS ANNUUS (SUNFLOWER) SEED OIL*, PERSEA GRATISSIMA (AVOCADO) OIL*, LECITHIN (SOYBEAN)*, CANNABIS SATIVA SEED OIL*, TALC, TITANIUM DIOXIDE, BUTYROSPERMUM PARKII (SHEA BUTTER)*, OLEA EURPOAEA (OLIVE) FRUIT UNSAPONIFIABLES, GARCINIA INDICA SEED BUTTER, ALGINIC ACID, ALUMINUM HYDROXIDE, CETEARYL ALCOHOL, CETEARYL WHEAT BRAN GLUCOSIDES, GLYCERYL STEARATE SE, HYDRATED SILICA, IRON OXIDE, XANTHAN GUM.	https://www.physiciansformula.com/organic-wear-natural-origin-tinted-moisturizer-spf-15.html

Philosophy	Ultimate Miracle Worker Facial Moisturizer	Active Ingredients: Avobenzone (2.7%), Octinoxate (6.99%), Octocrylene (2%). Inactive Ingredients: Water, C12-15 Alkyl Benzoate, Carthamus Tinctorius (Safflower) Oleosomes, Glycerin, Cyclopentasiloxane, Butylene Glycol, Methyl Gluceth-20, Gluconolactone, Polyacrylamide, Hydrogenated Lecithin, Cyclohexasiloxane, Cetyl Alcohol, Bifida Ferment Lysate, Phenoxyethanol, Ammonium Acryloyldimethyltaurate/Vp Copolymer, C13-14 Isoparaffin, Polysorbate 80, Acrylates/C10-30 Alkyl Acrylate Crosspolymer, Sodium Benzoate, Sodium Hydroxide, Laureth-7, Glycine Soja (Soybean) Oil, Fragrance, Iris Pallida Root Extract, Ruby Powder, Disodium Edta, Gluconic Acid, Alcohol, Mica, Lecithin, Adenosine, Cellulose Gum, Retinol, Peg-8, Polysorbate 20, BHT, Chitosan, Ethylhexylglycerin, Glycolic Acid, Sorbitan Oleate, Calcium Gluconate, Tocopherol, Behentrimonium Chloride, Sodium Bicarbonate, Ascorbyl Palmitate, Beta-Glucan, Potassium Phosphate, Caprylyl Glycol, 1,2-Hexanediol, Camellia Sinensis Leaf Extract, Coffea Arabica (Coffee) Seed Extract, Pongamia Pinnata Seed Extract, Isopropyl Alcohol, Acetic Acid, Angelica Archangelica Root Extract, Benzoic Acid, Citrus Aurantium Amara (Bitter Orange) Peel Extract, Maltodextrin, Citric Acid, Magnesium Aluminum Silicate, Xanthan Gum, Lactic Acid, Ascorbic Acid, Sclerotium Gum, BHA, Sorbic Acid, Titanium Dioxide (Ci 77891).	https://www.ulta.com/ultimate-miracle-worker-spf-30?productId=xlsImpprod1266 1073

Revlon	Age Defying CC Cream Color Corrector	Ingredients: Active: Octinoxate 7.5%, Octisalate 5.0%, Titanium Dioxide 5.1%...Purpose: Sunscreen. Inactive: Aqua (Water), Dimethicone, Neopentyl Glycol Diheptanoate, Butylene Glycol, Glycerin, Pentaerythrityl Tetraisostearate, Phenyl Trimethicone, Glyceryl Stearate, Tridecyl Trimellitate, Isotridecyl Isononanoate, Isostearic Acid, Boron Nitride, Alumina, Palmitoyl Tripepetide-5, Acetyl Hexapepetide-8, Glycyrrhiza Glabra (Licorice) Root Extract, Vitis Vinifera (Grape) Fruit Extract, Saxifrage Sarmentosa (Strawberry Saxifrage) Extract, Sodium Hyaluronate, Hydrolyzed Glycosaminoglycans, Scutellaria Baicalensis (Baikal Skullcap) Root Extract, Morus Bombycis (Chinese Mulberry) Root Extract, Ascorbyl Glucoside, Niacinamide, Dipotassium Glycyrrhizate, C13-14 Isoparaffin, Xanthan Gum, Simethicone, Laureth-7, Lecithin, Sorbitan Stearate, Polysorbate 20, Propylene Glycol Laurate, Propylene Glycol Stearate, Methicone, Stearic Acid, Steareth-2, Tromethamine, Polyacrylamide, Polymethyl Methacrylate, Steareth-21, Phenoxyethanol, Methylparaben, Ethylparaben. May Contain: Mica, Titanium Dioxide (Ci 77891), Iron Oxides (Ci 77491, 77492, 77499).	https://www.walmart.com/ip/Revlon-Age-Defying-CC-Cream-Color-Corrector-020-Light-Medium-1-Fl-Oz/35019153

Smashbox	Camera Ready Under Eye BB Cream	Octinoxate 7.50%, Octisalate 4.00%, Oxybenzone 2.50%, Titanium Dioxide 1.10%, Water, Dimethicone, Butylene Glycol, Phenyl Trimethicone, Pentylene Glycol, Glyceryl Stearate, Behenyl Alcohol, Trioctyldodecyl Citrate, Polymethylsilsesquioxane, Octyldodecyl Stearoyl Stearate, Peg-40 Stearate, Polyglyceryl-10 Pentastearate, Ammonium Acryloyldimethyltaurate/Vp Copolymer, Triticum Vulgare (Wheat) Germ Extract, Hordeum Vulgare (Barley) Extract/Extrait D'Orge, Cucumis Sativus (Cucumber) Fruit Extract, Laminaria Saccharina Extract, Pyrus Malus (Apple) Fruit Extract, Scutellaria Baicalensis Root Extract, Polyquaternium-51, Acetyl Hexapeptide-8, Trehalose, Oryzanol, Octyldodecyl Neopentanoate, Urea, Polyglyceryl-6 Polyricinoleate, Propylene Glycol Laurate, Glycyrrhetinic Acid, Tocopheryl Acetate, Sucrose, Glycerin, Sodium Stearoyl Lactylate, Caffeine, Linoleic Acid, Cholesterol, Lecithin, Squalane, Sodium Pca, Isopropyl Titanium Triisostearate, Propylene Glycol Stearate, Stearic Acid, Polysorbate 20, Sorbitan Laurate, Xanthan Gum, Aluminum Hydroxide, Sodium Hyaluronate, Silica, Disodium Edta, Pentaerythrityl Tetra-Di-T-Butyl Hydroxyhydrocinnamate, Sodium Dehydroacetate, Chlorphenesin, Phenoxyethanol, [+/- Titanium Dioxide (Ci 77891), Zinc Oxide (Ci 77947), Iron Oxides (Ci 77491, Ci 77492, Ci 77499), Mica]	https://www.sephora.com/product/camera-ready-bb-cream-spf-35-P300339
Tarte	BB Tinted Primer	Titanium Dioxide, Zinc Oxide, Cyclopentasiloxane, Isododecane, Mica, Polysilicone-11, Polymethylsilsesquioxane, Hexyl Laurate, PEG-10 Dimethicone, Polyglyceryl-4 Isostearate, Stearic Acid, Cetyl PEG/PPG-10/1 Dimethicone, Alumina, Triethoxycaprylylsilane, Dipalmitoyl Hydroxyproline, Diamond Powder, Iron Oxides.	https://www.sephora.com/product/bb-tinted-treatment-12-hour-primer-broad-spectrum-spf-30-sunscreen-P377542

Tarteguard	Mineral Powder Makeup	Titanium Dioxide 8.6%, Zinc Oxide 10.0%: Physical sunscreen. Vanilla: A natural fragrance with antioxidant and anti-aging properties. Polymethylsilsesquioxane, Silica, Jojoba Esters, Caprylyl Glycol, Alumina, Sodium Dehydroacetate, Phenoxyethanol, Vanillin, Caprylic/Capric Triglyceride, Hexylene Glycol, Vanilla Planifolia Fruit Extract, Aluminum Dimyristate, Triethoxycaprylylsilane, Disodium Stearoyl Glutamate, Iron Oxides, Mica.	https://www.sephora.com/product/tarteguard-mineral-powder-sunscreen-broad-spectrum-spf-30-P430951
Yardley of London	Moisturizing Bars	Sodium Tallowate, Sodium Cocoate, Water, Fragrance, Coconut Acid, Petrolatum, Glycerin, Sodium Chloride, Tetrasodium Etidronate, Pentasodium Pentetate, Tetrasodium EDTA, Iron Oxides, Titanium Dioxide	https://www.walgreens.com/store/c/yardley-of-london-moisturizing-bars-english-lavender/ID=prod6143589-product
Yes To Cucumbers	Mud Mask, Cooling, for Sensitive Skin	Aloe Barbadensis Leaf Juice, Water (Aqua), Kaolin, Bentonite, Propanediol, Hamamelis Virginiana (Witch Hazel) Water, Cetearyl Alcohol, Magnesium Aluminum Silicate, Titanium Dioxide (CI 77891), Glyceryl Stearate, Hydrogenated Vegetable Glycerides, Polyglyceryl-10 Oleate, Cucumis Sativus (Cucumber) Seed Extract, Cucumis Sativus (Cucumber) Fruit Water, Cucumis Sativus (Cucumber) Seed Oil, Curcuma Longa (Turmeric) Root Extract, Ocimum Basilicum (Basil) Flower/Leaf Extract, Ocimum Sanctum Leaf Extract, Brassica Oleracea Italica (Broccoli) Extract, Chamomilla Recutita (Matricaria) Flower Extract, Peucedanum Graveolens (Dill) Extract, Spinacia Oleracea (Spinach) Leaf Extract, Montmorillonite, Aloe Barbadensis Flower Extract, Solanum Melongena (Eggplant) Fruit Extract, Butyrospermum Parkii (Shea) Butter, Vitis Vinifera (Grape) Seed Oil, Melia Azadirachta Leaf Extract, Melia Azadirachta Flower Extract, Coccinia Indica Fruit Extract, Corallina O cinalis	https://www.ulta.com/cucumbers-calming-mud-mask?productId=xlsImpprod16411053

		Extract, Hydrolyzed Jojoba Esters, Ricinus Communis (Castor) Seed Oil, Glycerin, Dipotassium Glycyrrhizate, Illite, Xanthan Gum, Glyceryl Caprylate, Tetrasodium Glutamate Diacetate, Ethylhexylglycerin, Citric Acid, Phenoxyethanol, Iron Oxides (CI 77492), Fragrance (Parfum).	
